# Early IGF-1 receptor inhibition in mice mimics preterm human brain disorders and reveals a therapeutic target

**DOI:** 10.1126/sciadv.adk8123

**Published:** 2024-03-01

**Authors:** Alberto Potenzieri, Sara Uccella, Deborah Preiti, Matteo Pisoni, Silvia Rosati, Chiara Lavarello, Martina Bartolucci, Doriana Debellis, Federico Catalano, Andrea Petretto, Lino Nobili, Tommaso Fellin, Valter Tucci, Luca A. Ramenghi, Annalisa Savardi, Laura Cancedda

**Affiliations:** ^1^ Brain Development and Disease Laboratory, Istituto Italiano di Tecnologia, via Morego, 30, 16163 Genoa, Italy.; ^2^ Università degli Studi di Genova, via Balbi, 5, 16126 Genoa, Italy.; ^3^ Department of Neurosciences, Rehabilitation, Ophthalmology, Genetics, Maternal and Child Health (DINOGMI), University of Genoa, 16132 Genoa, Italy.; ^4^ Child Neuropsychiatry Unit, IRCCS Istituto Giannina Gaslini, Genoa, Italy.; ^5^ Patologia Neonatale, IRCCS Istituto Giannina Gaslini, Genoa, Italy.; ^6^ Optical Approaches to Brain Function Laboratory, Istituto Italiano di Tecnologia, via Morego, 30, 16163 Genoa, Italy.; ^7^ Core Facilities - Clinical Proteomics and Metabolomics, IRCCS Istituto Giannina Gaslini, via Gerolamo Gaslini 5, 16147 Genoa, Italy.; ^8^ Electron Microscopy Facility, Istituto Italiano di Tecnologia, via Morego, 30, 16163 Genoa, Italy.; ^9^ Genetics and Epigenetics of Behavior (GEB) Laboratory, Istituto Italiano di Tecnologia, via Morego, 30, 16163 Genoa, Italy.

## Abstract

Besides recent advances in neonatal care, preterm newborns still develop sex-biased behavioral alterations. Preterms fail to receive placental insulin-like growth factor-1 (IGF-1), a major fetal growth hormone in utero, and low IGF-1 serum levels correlate with preterm poor neurodevelopmental outcomes. Here, we mimicked IGF-1 deficiency of preterm newborns in mice by perinatal administration of an IGF-1 receptor antagonist. This resulted in sex-biased brain microstructural, functional, and behavioral alterations, resembling those of ex-preterm children, which we characterized performing parallel mouse/human behavioral tests. Pharmacological enhancement of GABAergic tonic inhibition by the U.S. Food and Drug Administration–approved drug ganaxolone rescued functional/behavioral alterations in mice. Establishing an unprecedented mouse model of prematurity, our work dissects the mechanisms at the core of abnormal behaviors and identifies a readily translatable therapeutic strategy for preterm brain disorders.

## Teaser

A mouse model mimics clinical features of preterm children and reveals tonic GABAergic current as a viable therapeutic target.

## INTRODUCTION

Recent advances in neonatal care units in the last 30 years have sharply increased the survival of premature newborns and drastically decreased the occurrence of severe brain lesions and associated neurological (motor and cognitive) deficits in this population (*1*). Nevertheless, nowadays, preterm newborns are still at high risk of developing more subtle neuropsychological deficits [impairments in visuospatial skills and cognitive performances, and alterations to sensory stimuli (*2*)] and important behavioral issues (social defects and maladaptive behaviors), which have been partially related to aberrant brain functional connectivity and delayed myelination (*3*, *4*). Decades of research in the past have investigated health issues related to prematurity due to birth complications such as hypoxia and cerebral blood flow instability as a primary cause of severe brain lesions associated to very serious neurological sequelae, with inflammation and oxidative stress introduced as more recent explanations for milder psychological/behavioral deficits of preterm babies (*3*). However, these lines of research did not focus on the intrinsic biology of prematurity and were originally designed based on the health needs of preterm babies who were born 30 years ago and who mostly presented with severe brain lesions (*1*). Although neonatal infection/inflammation, hypoxia, and fetal growth restriction are associated with severely impaired neurodevelopment (*5*), even minor or no brain lesions were linked to negative outcomes (*6*–*9*) within the context of an ongoing debate about impaired/delayed maturation of white matter in the preterm brain (*3*). Brain lesions such as intraventricular and cerebellar hemorrhage continue to be associated with brain disorders of prematurity in some preterms also nowadays and are thus yet an active field of investigation (*7*). On the other hand, we currently largely miss studies investigating further mechanisms underlying the brain microstructural abnormalities associated to still important behavioral deficits characterizing the other part of preterm babies, who are currently being born at earlier and earlier fetal stages nowadays, have no severe preterm birth complications, and mostly survive without brain lesions. These possible further mechanisms—intrinsic to the biology of prematurity—include loss of fundamental placental factors important for fetal development during the last trimester of pregnancy. As a result, there are still no approved pharmacological therapies to treat the cognitive/behavioral deficits of preterm babies.

Insulin-like growth factor type 1 (IGF-1) is a 70–amino acid peptide hormone with mitogenic, antiapoptotic, and metabolic functions. IGF-1 thus plays a fundamental role in promoting the growth and differentiation of several cell types, including brain cells (*10*). IGF-1 exerts its primary effect upon binding to the IGF-1 receptor (IGF-1R) (*10*), a typical tyrosine kinase receptor expressed in many cell types. During prenatal life, human placenta promotes the increase in IGF-1 levels to support fetal growth [both directly and indirectly (*11*)], particularly during mid-late gestation (*12*). In preterm babies, the IGF-1–promoting effect of placenta during the third trimester of pregnancy is obviously lost. Accordingly, premature newborns have dramatically lower serum levels of IGF-1 than age-matched fetuses when still in utero (*12*–*14*). Low postnatal levels of IGF-1 are associated with several complications of prematurity, including retinopathy of prematurity (ROP) (*15*), bronchopulmonary dysplasia (*16*), and poor neurodevelopmental outcome at 2 years of age (*17*).

Here, we showed that transient IGF-1 signaling inhibition during the mouse counterpart of the human third trimester of pregnancy resulted in brain microstructural, functional, and behavioral alterations in mice resembling brain disorders typical of children born preterm nowadays. In particular, we paralleled our mouse studies to the behavioral assessment (social, visuospatial, and learning abilities at 5 years of age) of a small pilot cohort of ex-premature children born in the current decade and free of brain lesions. Moreover, histochemistry and electrophysiological analyses of our mouse model of preterm brain disorders uncovered increased inhibitory-neuron cell death, decreased GABAergic tonic inhibition, and increased neuronal excitability in adolescent male mice, which were more affected than females. Treatment with the U.S. Food and Drug Administration (FDA)–approved GABA_A_ receptor (GABA_A_R) positive allosteric modulator (PAM) ganaxolone (GNX) normalized neuronal excitability and rescued cognitive impairment and social/repetitive behaviors in male mice and anxiety in female mice.

Our data indicate that IGF-1 signaling during the time window of prematurity is an essential neurotrophic factor and suggest GABA_A_R PAMs as a potential strategy to address preterm brain disorders later in life. Finally, we present here a mouse model of prematurity and a rapid and noninvasive strategy to evaluate behavioral alterations in ex-premature children that parallel mouse studies, which are both valid assets for future interdisciplinary research in prematurity and drug-discovery programs.

## RESULTS

### Early IGF-1R inhibition in mouse pups leads to acute phospho-proteomic changes associated with neuropsychiatric disorders

**Fig. 1.**
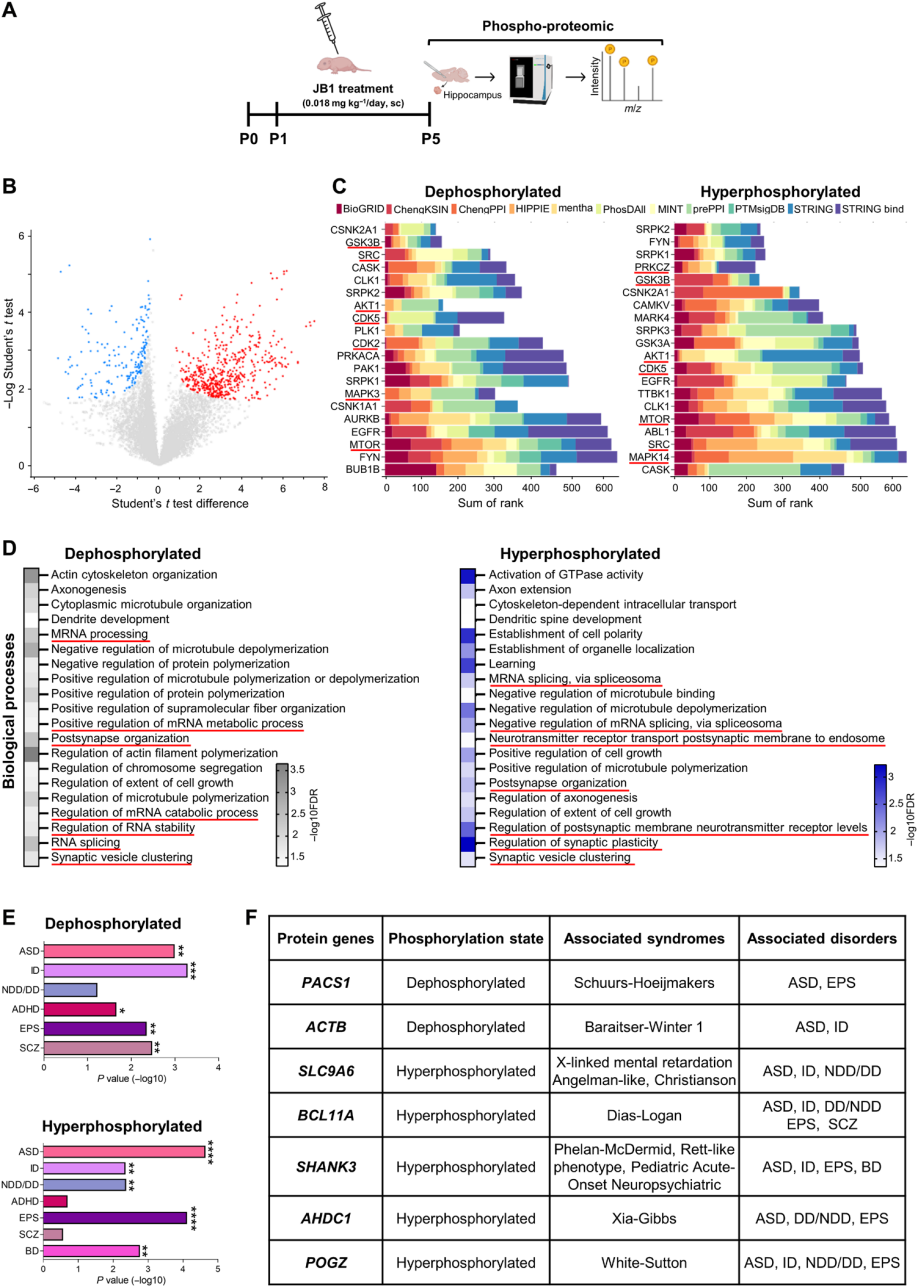
Systemic IGF-1R inhibition in mouse pups leads to acute phospho-proteomic changes associated with neuropsychiatric disorders. (**A**) Experimental protocol with pharmacological treatment and timing of the phospho-proteomic experiment utilizing mass spectrometry. (**B**) Volcano plot showing the phosphorylated/dephosphorylated phosphorylation sites differentially expressed between JB1-treated (*N* = 10 animals) and control (vehicle-treated; *N* = 8 animals) pup littermates euthanized 1 hour after the last treatment at P5. Blue and red dots represent significantly dephosphorylated and hyperphosphorylated phosphorylation sites, respectively. s0 = 0.1 and FDR = 0.05. (**C**) Kinase Enrichment Analysis of significantly dephosphorylated (left) and hyperphosphorylated (right) phospho-proteins, corresponding to differentially expressed phosphorylation sites shown in (B). Bars represent the mean rank of the top 20 phospho-proteins based on multiple library databases (color-coded above). The kinases related to IGF-1 signaling are underlined in red. (**D**) Gene Ontology (GO) analysis for the differentially dephosphorylated (left) or hyperphosphorylated (right) significantly expressed proteins corresponding to differentially expressed phosphorylation sites shown in (B). The color bar on the right indicates −log10FDR for the statistically significant (FDR < 0.05) top 20 (hierarchy for fold enrichment) enriched biological processes. The terms related to synapses or RNA processes are underlined in red. (**E**) Enrichment for neuropsychiatric disorder risk genes (identified with SFARI gene archive) in the dephosphorylated (top) and hyperphosphorylated (bottom) protein datasets shown in (B). Hypergeometric test, *P < 0.05, **P < 0.01, ***P < 0.001, ****P < 0.0001. (**F**) Table showing high confidence, syndromic neuropsychiatric disease genes (category 1S of the SFARI gene archive) present in significantly dephosphorylated and hyperphosprylated proteins in (B). ADHD, attention-deficit/hyperactivity disorder; ASD, autism spectrum disorder; BD, bipolar disorder; EPS, epilepsy; ID, intellectual disability; NDD/DD, neurodevelopmental disorder/developmental disorder; SCZ, schizophrenia. Schematic cartoons by BioRender.com.

To mimic systemic IGF-1 reduction in severe and moderate preterm newborns [i.e., 23–32 weeks of human gestational age (*18*)], we administered JB1 [an IGF-1 peptide mimetic and IGF-1R–specific antagonist; 0.018 mg kg^−1^, subcutaneously, once daily (*19*)] during the corresponding mouse developmental stage [i.e., from postnatal day (P)1 to P5 (*18*); Fig. 1A]. We chose to inhibit IGF-1R by treatment with JB1 versus standard small-molecule inhibitors due to the substantial similarity between JB1 and IGF-1 [in terms of their peptide nature and large dimensions versus chemical structures and small dimensions of classical inhibitors (*20*)]. JB1-treated pups did not show any mortality or significant alteration in body weight during the treatment period (P1 to P5) compared to their vehicle (saline)–treated control littermates (fig. S1, A and B). Conversely, JB1-treated males (but not females) showed a significant body weight reduction compared to controls later in life (P28, early adolescence; fig. S1C). As control, we also measured plasma and hippocampal levels of IGF-1 [by enzyme-linked immunosorbent assay (ELISA)] and plasma levels of IGF binding proteins 2, 3, and 4 (IGFB2, IGFB3, and IGFB4, respectively), IGF acid-labile subunit (IGFALS), and IGF-2 (by liquid chromatography-mass spectrometry), and we found no statistically significant difference between JB1- and saline-treated littermates (table S1).

IGF-1R signaling activation triggers an intracellular cascade of phosphorylations at downstream proteins (*21*). Thus, we first evaluated the acute effect of JB1 treatment on neonatal hippocampus (a brain area fundamental for learning and memory) by phospho-proteomic analysis of JB1-treated animals versus their saline-treated littermates 1 hour after the last injection of the treatment at P5 (Fig. 1A). We found 310 (significant) differentially dephosphorylated and 551 (significant) hyperphosphorylated phosphorylation sites between the two experimental groups (Fig. 1B and fig. S2, A and B), corresponding to 137 (significant) differentially dephosphorylated and 186 (significant) hyperphosphorylated proteins. We then performed a Kinase Enrichment Analysis 3 [KEA3, (*22*)] to reveal upstream kinases involved in the downstream phospho-proteomic changes that we observed. As expected, by KEA3 analysis, we found several kinases downstream of the IGF-1R signaling pathway (e.g., AKT, MAPK, and GSK3β) in both the dephosphorylated and hyperphosphorylated protein datasets (Fig. 1C). Next, to assess what biological processes, cellular compartments, and molecular functions were linked to the dysregulations of the phospho-proteome in JB1-treated pups, we performed a Gene Ontology (GO) enrichment analyses (*23*) of the significantly dephosphorylated and hyperphosphorylated proteins. GO biological process enrichment analysis showed that both dephosphorylated and hyperphosphorylated proteins were enriched for terms related to neuronal synapses and RNA processes (Fig. 1D) in JB1-treated pups, suggesting that JB1 treatment mainly interfered with neuron development and that its effect could have long-term consequences. Synapses, dendrites, and axons were also enriched terms for GO cellular compartment analysis, whereas in the analysis for GO molecular functions, we found enrichment in terms related to actin dynamics, GTPase (guanosine triphosphatase) process, and kinase binding (fig. S2C) in JB1-treated pups. We also performed a Reactome pathway analysis (*24*) to find molecular pathways possibly involved in the effect induced by JB1 treatment. We found a dysregulation of pathways related to apoptosis, GTPase cycles, and mRNA splicing (fig. S2C). Finally, to start to investigate a possible relationship between the deregulation of the phospho-proteome by an early IGF-1R inhibition and gene signatures of neuropsychiatric disorders, we assessed the presence of neuropsychiatric risk genes in our dephosphorylated or hyperphosphorylated protein dataset using the Simons Foundation Autism Research Initiative (SFARI) tool. We found that a significant number of risk genes associated to neuropsychiatric disorder by the SFARI database corresponded to the presence of their related proteins among our dephosphorylated and hyperphosphorylated protein datasets (Fig. 1E). Among the neuropsychiatric disorders associated to combinations of some risk genes that corresponded to our phosphoprotein database, the SFARI tool indicated that the most significant were intellectual disability (ID) for our dephosphorylated dataset, and autism spectrum disorder (ASD) for our hyperphosphorylated dataset. Furthermore, in our datasets, we also found proteins corresponding with high confidence level (category 1S of SFARI) to specific single genes well-known to be strongly associated to neuropsychiatric and ASD-related syndromes (e.g., *BCL11A* and *SHANK3*; Fig. 1F).

These results indicate that acute JB1 treatment in mouse pups leads to phospho-proteomic changes—downstream of IGF-1 kinase signaling pathway—that mainly associate with neuronal and RNA processes. These changes are enriched for proteins associated with genes involved in neuropsychiatric disorders characterized by deficits in cognitive and social domains, such as ID and ASD.

### Early IGF-1R inhibition leads to behavioral alterations in postnatal and adolescent mice

**Fig. 2.**
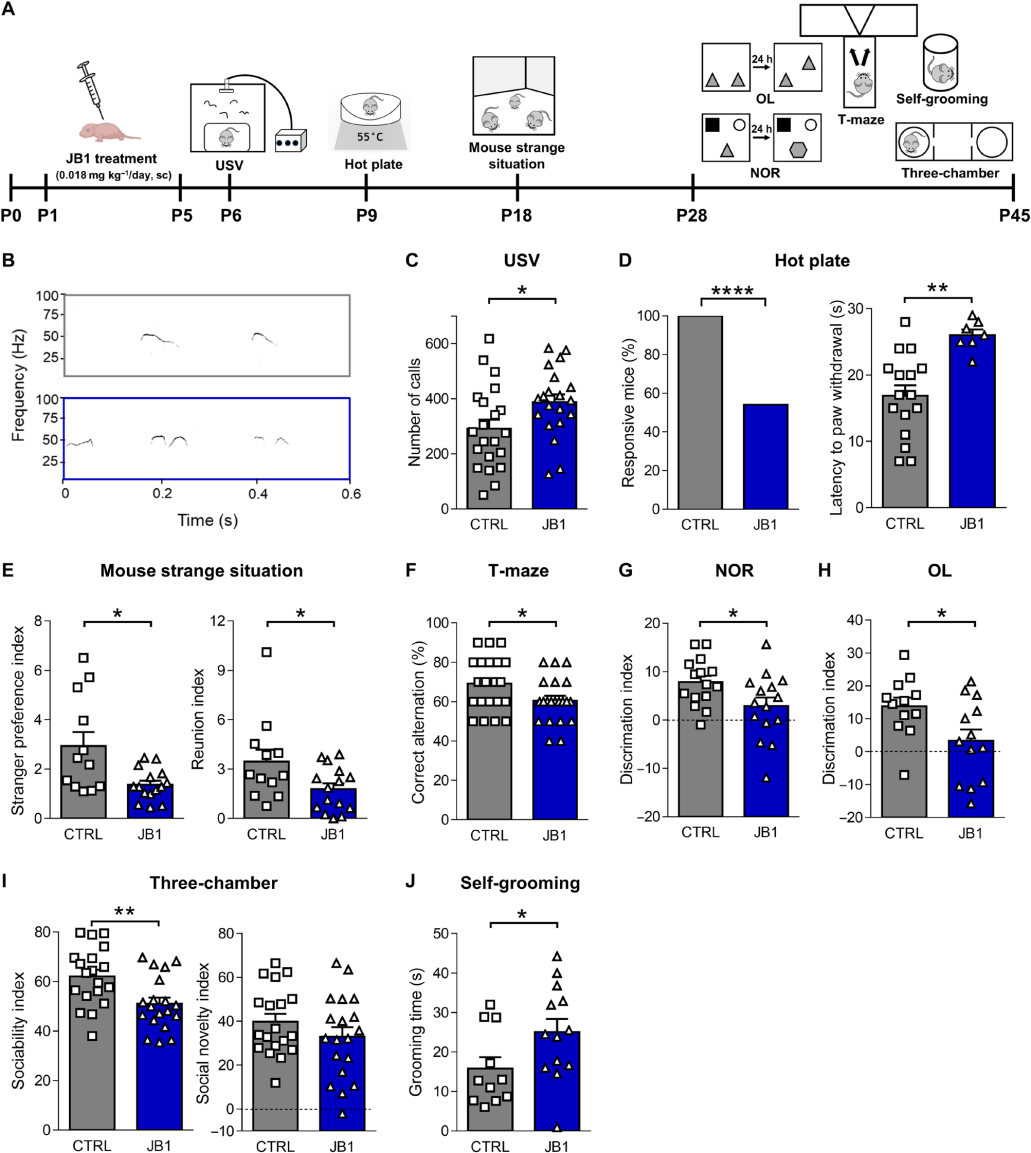
Early postnatal IGF-1R inhibition leads to behavioral alterations. (**A**) Experimental protocol. (**B**) Example of a fraction of the recordings of ultrasonic vocalizations (USVs) emitted by P6 pups. (**C**) Quantification of the mean ± SEM (bars) and single animal cases (symbols) of the number of USV calls in the experiments as in (B). Two-tailed Student’s *t* test, *t* = 2.221, *P < 0.05. (**D**) Quantification of the mean ± SEM and single animal cases of the number of P9 responsive pups and their latency to first paw withdrawal after placement on a hot plate. Left, Fisher’s exact test, ****P < 0.0001. Right, two-tailed Student’s *t* test, *t* = 3.713, **P < 0.01. (**E**) Quantification of the mean ± SEM and single animal cases of the stranger preference index and reunion index during the mouse strange situation test. Mann-Whitney test, *U* = 41.0 (left), *U* = 48.0 (right) *P < 0.05. (**F**) Quantification of the mean ± SEM and single animal cases of the correct alternations. Two-tailed Student’s *t* test, *t* = 2.309, *P < 0.05. (**G**) Quantification of the mean ± SEM and single animal cases of the discrimination index. Two-tailed Student’s *t* test, *t* = 2.285, *P < 0.05. (**H**) Quantification of the mean ± SEM and single animal cases of the discrimination index. Two-tailed Student’s *t* test, *t* = 2.398, *P < 0.05. (**I**) Quantification of the mean ± SEM and single animal cases of the sociability index and social novelty index. Left, two-tailed Student’s *t* test, *t* = 3.006, **P < 0.01. (**J**) Quantification of the mean ± SEM and single animal cases of the grooming time. Mann-Whitney test, *U* = 36.0, *P < 0.05. For all experiments, the analyzed animals for each experimental group were derived from three to eight independent litters. Schematic cartoons by BioRender.com.

Because premature birth leads to long-term behavioral consequences, we examined whether IGF-1R blockade during the critical period of prematurity led to similar behavioral alterations in mice by performing a battery of tests from P6 to P45 (Fig. 2A). We began our assessment by addressing social communication abilities, somatosensory perception, and attachment behavior in the early postnatal life (P6 to P18) of JB1-treated pups versus their vehicle-treated control littermates (Fig. 2A). JB1-treated pups displayed altered social communication, with an increased number of ultrasonic vocalization (USV) calls and an increased probability of emitting chevron calls compared to saline-treated littermates when individually separated from their dam for 5 min (Fig. 2, B and C, and table S2). This is in agreement with mouse models of ASD (*25*). Moreover, JB1-treated pups showed altered thermal sensitivity in the hot plate test, with ~45% of them showing no reaction and ~55% showing a longer latency in their response when compared to their control littermates (Fig. 2D). Finally, JB1-treated pups showed insecure attachment bonding with their mother compared to control littermates, which we assessed by analyzing the exploration of a stranger mouse in the presence of the mother (expressed as the stranger preference index, Fig. 2E) and comfort-seeking behavior during reunion with the mother after exposure to the stranger mouse alone (reunion index, Fig. 2E) (*26*). JB1-treated pups also showed a nonsignificant reduction in both the exploration of the stranger mouse when exposed to it alone (stranger effect index) and in maternal preference during reunion with the mother (maternal preference index) compared to control littermates (fig. S1D).

Next, we assessed whether early IGF-1R inhibition could induce long-term behavioral alterations during adolescence, which is a period of great behavioral vulnerability in people who were born preterm (*4*). To this aim, we tested memory performances (short-term memory and long-term explicit memory), the presence of social deficits, repetitive behaviors, and anxiety in postweaning JB1-treated mice (P28 to P45). We found a significant alteration in short-term memory, as assessed by the T-maze task, when we analyzed the correct choices of the previously unexplored arm (Fig. 2F). Moreover, JB1-treated mice showed poor novel discrimination ability in the novel object recognition (NOR) test (Fig. 2G) and poor spatial memory in the object location (OL) test (Fig. 2H). Furthermore, JB1-treated mice showed poor social interaction when exposed to a never-met intruder versus an object (sociability index) and no nonsignificant effect when exposed to a novel mouse versus an already-met mouse (social novelty index) in the three-chamber test (Fig. 2I). JB1-treated mice also showed a significant increase in repetitive behaviors in the self-grooming test (Fig. 2J). When we tested for anxiety behavior by the elevated plus maze (EPM) task, we only found a nonsignificant trend versus increased anxiety in JB1-treated mice, as assessed by the decreased number of entries and the decreased time spent in the open arms of the maze (fig. S1E). Furthermore, JB1-treated mice showed no significant alteration in locomotor activity in the open field test (fig. S1F).

These data indicate that the inhibition of IGF-1 signaling in mice during the time window of prematurity leads to short- and long-term behavioral alterations, which recapitulate those described in the literature for people who were born preterm.

### Early IGF-1R inhibition leads to brain microstructural and functional changes in adolescent mice

Because preterm newborns nowadays may show long-term brain microstructural alterations, including decreased white matter myelination [a clinical hallmark of preterm brain disorder (*1*)] and a reduced number of cortical interneurons (*27*), we investigated whether interfering with IGF-1 signaling early in development leads to similar alterations in JB1-treated mice when they are adolescents.

**Fig. 3.**
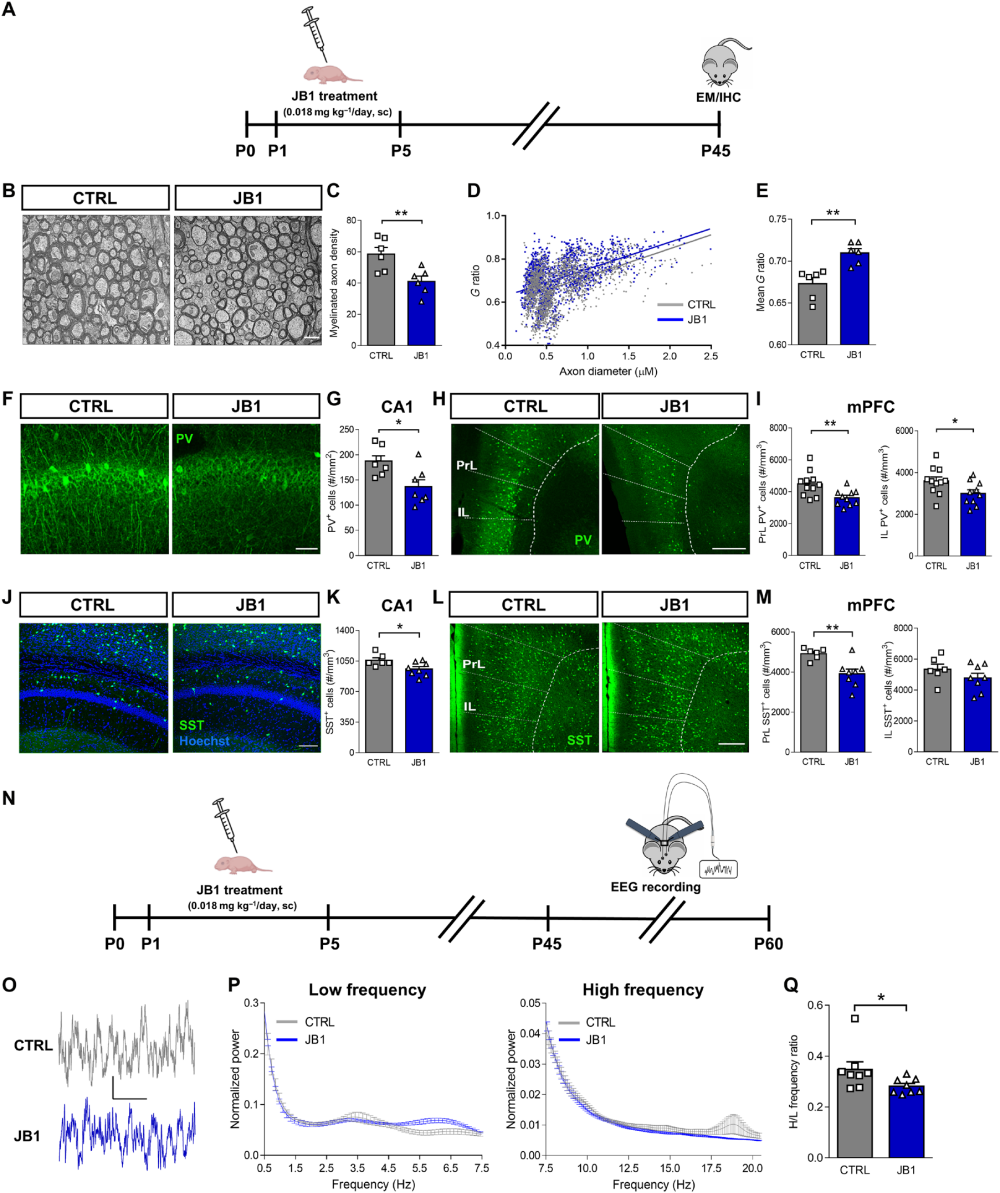
Early postnatal IGF-1R inhibition leads to brain anatomical microstructural and functional changes. (**A**) Experimental protocol. (**B**) Corpus-callosum TEM images from P45 mice. Scale bar, 1 μm. (**C**) Average myelinated-axon density in the view field. Two-tailed Student’s *t* test, *t* = 3.210, **P < 0.01. (**D**) Scatte plot of the *G* ratio versus axon diameter. Fitted lines: linear regressions, *R*^2^: CTRL = 0.2080, JB1 = 0.2409. (**E**) Average *G* ratio for each animal. Two-tailed Student’s *t* test, *t* = 4.121, **P < 0.01. Three independent experiments. (**F**) Images of PV fluorescence in CA1-hippocampal slices from P45 mice. Scale bar, 60 μm. (**G**) Average density of PV interneurons in brain slices. (two to three slices per animal, four litters). Two-tailed Student’s *t* test, *t* = 2.918, *P < 0.05. (**H**) Images of PV fluorescence in mPFC slices, with indicated subregions (PrL, prelimbic; IL, infralimbic; white dotted lines) from P45 mice. Scale bar, 100 μm. (**I**) Average density of PV interneurons in brain slices (two to three slices per animal, five litters). Two-tailed Student’s *t* test; left, *t* = 3.027, **P < 0.01; right, *t* = 2.243, *P < 0.05. (**J**) Images of SST and Hoechst fluorescence in CA1-hippocampal slices from P45 mice. Scale bar, 60 μm. (**K**) Average density of SST interneurons in brain slices (two to three slices per animal, three litters). Two-tailed Student’s *t* test, *t* = 2.499, *P < 0.05. (**L**) Images of SST fluorescence in mPFC slices, with indicated subregions, from P45 mice. Scale bar, 60 μm. (**M**) Average density of SST interneurons in brain slices (two to three slices per animal, three litters). Left, two-tailed Student’s *t* test, *t* = 3.465, **P < 0.01. (**N**) Experimental protocol. (**O**) EEG traces from P>45 mice. Scale bars, 0.1 mV, 5000 ms. (**P**) Average normalized EEG power spectra ± SEM for low and high frequencies of mice recorded at P45–60 (*N* = 8 JB1, *N* = 8 CTRL). (**Q**) High-/low-frequency (H/L) ratio. Five litters. Mann-Whitney test, *U* = 10.00, *P < 0.05. For (C), (E), (G), (I), (K), (M), and (Q), bars represent the average ± SEM, and symbols represent single data points for each animal. Schematic cartoons by BioRender.com.

To assess the presence of myelin defects, we first quantified the expression of proteolipid protein-1 (PLP-1) and myelin basic protein (MBP), two major myelin proteins, by semiquantitative immunofluorescence analysis in the corpus callosum (white matter) at P45. We found that JB1-treated mice showed reduced expression of PLP-1 and MBP compared to controls (fig. S3, A to C). Furthermore, ultrastructural analysis by transmission electron microscopy (TEM) revealed a marked decrease in myelinated axon density and a thinner myelin sheath of the axons (quantified by *G* ratio analysis; *G* ratio = inner axon diameter/total axon diameter with myelin), with no differences in the axon diameter caliber (mean CTRL = 0.66 ± 0.02, *N* = 6; mean JB1 = 0.67 ± 0.024, *N* = 6; two-tailed Student’s *t* test, *t* = 0.4643; *P* value = 0.6524) in the corpus callosum (Fig. 3, A to E). In agreement with myelination defects, we found that JB1-treated mice showed a reduced number of oligodendrocytes in the corpus callosum, which we assessed by immunofluorescence analysis of OLIG2 (marker of oligodendrocyte lineage)–positive cells at P45 (fig. S3, D and E).

Then, we assessed the number of interneurons in JB1-treated mice at P45 in the CA1 subregion of the hippocampus and in both the prelimbic (PrL) and infralimbic (IL) subregions of the medial prefrontal cortex (mPFC), a brain region implicated in both social and anxiety behaviors (*28*). We found that JB1-treated mice showed a reduced number of parvalbumin-positive (PV^+^) interneurons in these hippocampal and prefrontal cortex subregions, as assessed by immunohistochemistry (Fig. 3, F to I). Moreover, JB1-treated mice showed a reduced number of somatostatin-positive (SST^+^) interneurons in both the CA1 hippocampal region and the PrL region of the mPFC (Fig. 3, J to M), as assessed in mice expressing TdTomato upon Cre recombination specifically in somatostatin interneurons.

Finally, we assessed whether early IGF-1R inhibition leads to an electroencephalographic (EEG) signature similar to that reported in young adults born preterm (*29*). In particular, we recorded frontoparietal EEG in head-fixed awake mice previously habituated to the setup (Fig. 3, N and O) to mimic resting-state EEG recordings in preterm humans. As previously described in human data (*29*), we found a lower high-frequency power–to–low-frequency power ratio in JB1-treated mice (Fig. 3, P and Q), indicating increased relative power in low-frequency bands and decreased power in high-frequency bands. Moreover, power spectral density analysis showed a significant change in theta power among all frequency bands analyzed (fig. S1, G to M).

These data suggest that the inhibition of IGF-1 signaling in mice during the time window of prematurity leads to long-term brain anatomical and functional alterations, similar to those observed in people who were born preterm.

### Early IGF-1R inhibition leads to increased intrinsic excitability and reduced GABAergic inhibition in CA1 pyramidal neurons

**Fig. 4.**
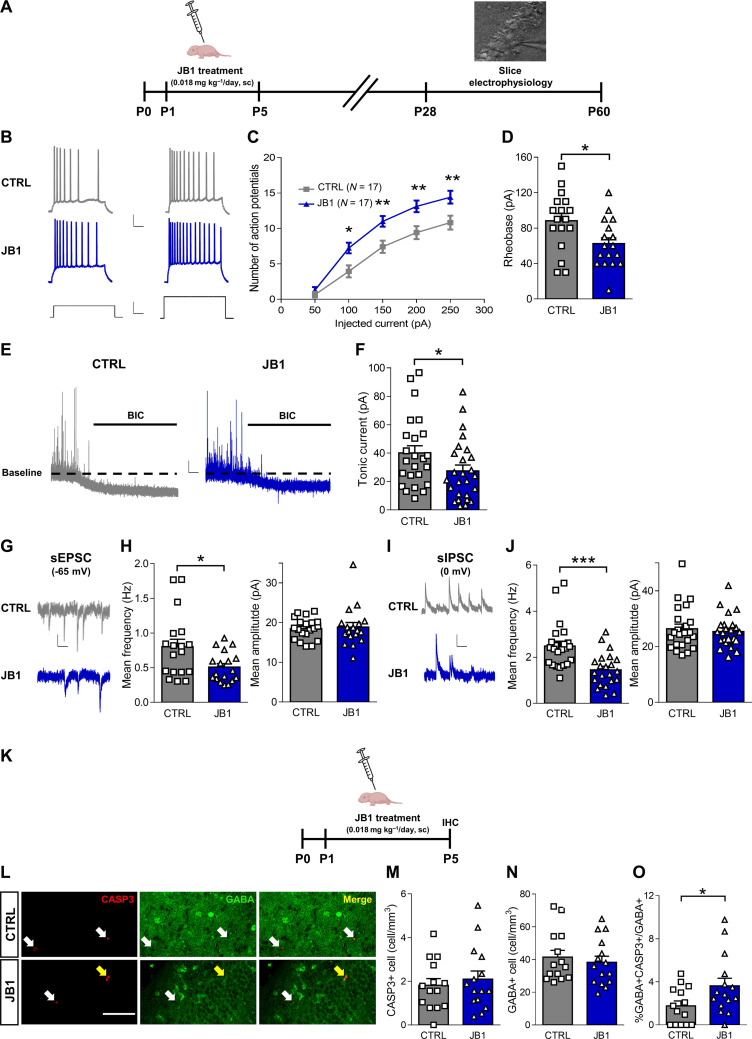
Early postnatal IGF-1R inhibition leads to increased intrinsic excitability, decreased synaptic transmission, reduced GABAergic-tonic inhibition in CA1 pyramidal neurons, and increased apoptosis of GABAergic interneurons. (**A**) Experimental protocol. (**B**) Current-clamp traces of membrane potential changes (top) in response to depolarizing current-steps (150 pA left, 250 pA right; bottom) in CA1-hippocampal pyramidal neurons (P>40 mice). Scale bars, traces: 20 mV, 100 ms; stimuli: 150 pA, 100 ms. (**C**) Average action-potential numbers (±SEM) elicited by current steps. In parentheses: number of recorded cells (eight CTRL–nine JB1 littermates, five litters). Two-way ANOVA RM, *F*_treatment_(1,32) = 9.590, *P* < 0.01, Sidak post hoc test, *P < 0.05, **P < 0.01. (**D**) Average Rheobase of recorded cells (eight CTRL–nine JB1 littermates, five litters). Two-tailed Student’s *t* test, *t* = 2.466, *P < 0.05. (**E**) Voltage-clamp traces of bicuculline-induced (bar) tonic currents in CA1-hippocampal pyramidal neurons (P>35 mice). Scale bars, 15 pA, 500 ms. (**F**) Amplitude averages of tonic currents for recorded cells (11 CTRL–11 JB1 littermates, four litters). Mann-Whitney test, *U* = 210.0, *P < 0.05. (**G**) Voltage-clamp traces of sEPSCs (−65 mV) in CA1-hippocampal pyramidal neurons (P>40 mice). Scale bars, 10 pA, 250 ms. (**H**) sEPSC frequency and amplitude averages of recorded cells (nine CTRL–eight JB1 littermates, four litters). Left, Mann-Whitney test, *U* = 98.0, *P < 0.05. (**I**) Voltage-clamp traces of sIPSCs (0 mV) in CA1-hippocampal pyramidal neurons (P>35 mice). Scale bar, 10 pA, 250 ms. (**J**) sIPSC frequency and amplitude averages for recorded neurons (12 CTRL–11 JB1 littermates, four litters). Left, Mann-Whitney test, *U* = 96.00, ***P < 0.001. (**K**) Experimental protocol. (**L**) Caspase-3 (CASP3) and GABA fluorescence in CA1-hippocampal slices from P5 littermates. Scale bar, 60 μm. White arrows: CASP3^+^ cells. Yellow arrows: CASP3^+^/GABA^+^ cells. (**M**) Average density of CASP3^+^ cells (two to three slices per animal, four litters). (**N**) Average density of GABA^+^ cells (two to three slices per animal, four litters). (**O**) Average density of CASP3^+^/GABA^+^ cells normalized on total GABA^+^ cells (two to three slices per animal, four litters). Two-tailed Student’s *t* test, *t* = 2.150, *P < 0.05. For (D), (F), (H), (J), (M), (N), and (O), bars represent average ± SEM, and symbols represent data for each recorded cell/animal. Schematic cartoons by BioRender.com.

To begin our investigation on possible cellular mechanisms underlying behavioral phenotypes in JB1-treated mice, we assessed whether a reduction in IGF-1R signaling could affect neuronal physiology by performing whole-cell, patch-clamp recordings in the CA1 region of acute hippocampal slices from adolescent mice (Fig. 4A). First, we evaluated changes in the intrinsic excitability of the CA1 pyramidal neurons. Pyramidal neurons from JB1-treated mice fired a higher number of action potentials (APs) in response to graded depolarizing current steps than those from controls (Fig. 4, B and C). Accordingly, adolescent JB1-treated mice showed a significantly lower rheobase current (the minimal current required to evoke an AP) than controls (Fig. 4D). The increased intrinsic excitability of JB1-treated mice was not due to alterations in passive (i.e., input resistance) or active (i.e., AP threshold) properties (table S3). Next, our results on the reduced number of PV^+^ and SST^+^ interneurons in JB1-treated mice suggested to investigate deficient neuronal (GABAergic) inhibitory tone as a possible mechanism underlying increased CA1 pyramidal neuron excitability (as assessed by the AP recordings). Thus, because GABA exerts potent control over neuronal excitability through the activation of extrasynaptic GABA_A_R-mediated tonic inhibition (*30*), we investigated GABAergic tonic currents in JB1-treated mice and control vehicle-treated littermates. To reveal tonic GABAergic inhibition, we bath-applied the GABA_A_R antagonist bicuculline (20 μM) to acute brain slices from adolescent mice. Bicuculline application reduced the holding current in the recorded neurons (and abolished spontaneous phasic GABAergic events), revealing a tonically active inhibitory tone in both experimental groups (Fig. 4E). Notably, adolescent JB1-treated mice showed a lower tonic current than controls (Fig. 4, E and F). When we evaluated the expression of the chloride transporters NKCC1 and KCC2, which are often dysregulated in neurodevelopmental disorders (*31*), we did not find any significant difference in the expression of the two cotransporters (fig. S3, F and G), suggesting that the increased excitability was not dependent on impaired chloride homeostasis.

We also recorded spontaneous excitatory and spontaneous inhibitory postsynaptic currents (sEPSC and sIPSC, respectively) in pyramidal neurons, and we found that JB1-treated mice showed decreased frequency, but not amplitude, of both sEPSCs and sIPSCs (Fig. 4, G to J).

Finally, we investigated how early IGF-1 blockade could eventually result in defective GABAergic tonic and phasic currents in adolescent mice. Because we reasoned that those impaired GABAergic currents most likely depended on the reduced number of interneurons we found in the JB1-treated mice, we focused our investigation on the possible mechanism linking early acute IGF-1 inhibition to the loss of GABAergic neurons. In particular, we considered programmed cell death (apoptosis), because we found apoptosis cleavage of cellular proteins among the altered pathways in our phospho-proteomic analysis (fig. S2C), and because IGF-1 is a known regulator of apoptosis (*32*). In rodents, large programmed cell death occurs during the first two postnatal weeks, a process crucial for the establishment of the final number of neurons within the brain (*33*). To assess whether acute IGF-1R inhibition lead to increased interneuron apoptosis in JB1-treated mice, we performed double staining for GABA [PV is not expressed until P14 (*34*)] and Caspase-3 [known marker of apoptosis (*35*)] on the last day of JB1 treatment (P5; Fig. 4K), and we counted double-positive cells in the CA1 hippocampal region (Fig. 4L). We found that acute JB1 treatment increased the percentage of GABA^+^ and Caspase-3^+^ cells over the total number of GABA^+^ neurons, without any significant changes in the number of total GABA^+^ cells or total Caspase-3^+^ cells (Fig. 4, M to O). We found a similar (but not significant) trend also in the mPFC at P7 (fig. S4, A to E), the peak of interneuron apoptosis during development of the cerebral cortex (*33*).

These data suggest that the inhibition of IGF-1 signaling in mice during the time window of prematurity increases GABAergic interneuron apoptosis and leads to a long-term decrease in tonic and phasic GABAergic currents, with a reduction also in the frequency of sEPSC.

### Early IGF-1R inhibition leads to sex-specific alterations in adolescent mice

Clinical literature evidence indicates better cognitive and behavioral outcomes in preterm females than in males (*36*), but the molecular mechanism behind these differences is poorly understood. We hypothesized that GABA-mediated tonic inhibition may be a possible mechanism underlying the sex-biased difference in behavioral outcomes in males and females born preterm. Tonic inhibition is modulated by neurosteroids (e.g., allopregnanolone), and neurosteroids are synthetized from sexual hormones (*37*) and are differently expressed in females and males (*37*). To evaluate the possibility that GABA-mediated tonic inhibition may be a possible mechanism underlying the sex-biased difference in behavioral outcomes in males and females born preterm, we first segregated our tonic current data according to the sex of the recorded animals. Notably, we found a significant reduction in tonic inhibition only in male mice, whereas females showed a nonsignificant trend (fig. S5, A and B). Rheobase current injection-neuronal firing input–output curves (and sIPSCs, but not sEPSCs) were also significantly impaired only in males (fig. S5, C to F), suggesting a link between the hippocampal increased excitability and the reduced tonic inhibition in male mice treated with JB1.

Next, to evaluate a possible link between electrophysiological, anatomical, molecular, and behavioral phenotypes, we also segregated phosphoproteomic, behavioral, and anatomical data according to mouse sex. When we segregated our phosphoproteomic data according to the sex of the animals, we found 58 (significant) differentially dephosphorylated and 197 (significant) hyperphosphorylated phosphorylation sites between the control and JB1-treated male mice (fig. S6, A to C). Those corresponded to 37 (significant) differentially dephosphorylated and 90 (significant) hyperphosphorylated proteins. As in the analysis we performed on the phosphoproteome of male and female pups together, KEA3 (*22*) in males only again revealed several kinases downstream of IGF-1R signaling pathway (e.g., AKT, MAPK, and GSK3β) among the phosphoproteomic changes that we observed, in both the dephosphorylated and hyperphosphorylated protein datasets (fig. S6D). When we performed a gene signature analysis of neuropsychiatric disorders using the SFARI tool, we found that the only significant associated disorder in males was ASD (fig. S6E). GO enrichment analyses (*23*) confirmed the prevalent involvement of neurons as cellular compartment while both GO biological process and molecular functions (found altered only in the hyperphosphorylated dataset) showed enrichment in general cellular processes and function, pointing out to general neurodevelopmental effects (fig. S6F). Conversely, we found that only five hyperphosphorylated phosphorylation sites (corresponding to four proteins, table S4) reached statistical significance when comparing control and JB1-treated female datasets. This low number of proteins did not allow us to perform any further omics analysis.

Nevertheless, when we performed a more permissive analysis [S0 = 0.1; *P* value < 0.01 versus False Discovery Rate (FDR) = 0.05], we did find 60 dephosphorylated proteins (table S5) and 46 hyperphosphorylated proteins (table S6) as dysregulated when compared to control and JB1-treated female mice. When we replicated the same analysis performed in males, we found dysregulated IGF-1 signaling (with KEA, table S7) and gene signature of neuropsychiatric disease (with SFARI tool, table S8) and neuronal and protein binding alterations (with GO analysis, table S9). These findings were all in line with the phosphoproteome results we found in males only and with the phosphoproteome analysis we performed independent of the sex of the animals. Moreover, these findings indicate only a nonsignificant trend of the effect of JB1 treatment on the phosphoproteome in females versus the significant effect in males, paralleling what we found for the tonic GABAergic current analysis.

In full parallel with the GABAeric current and phosphoproteome analyses, we found that only JB1-treated males showed a significant alteration in memory performance (T-maze and NOR tasks, but not the OL test; fig. S7, A to D), as well in social (three chamber task; fig. S7, E and F) and repetitive behaviors (self-grooming task; fig. S7G) at the behavioral level, with only nonsignificant trends in females. Conversely, we found that only JB1-treated females showed statistically significant increased anxiety (EPM test; fig. S8, A to C), in line with the literature in humans. Neither female nor male mice treated with JB1 showed significantly altered locomotor activity (open field test; fig. S8, D to G).

Regarding anatomical defects, both male and female mice showed reduced myelination, although females did not reach statistical significance (fig. S9). The reduction in the number of CA1 PV^+^ and PrL SST^+^ interneurons was significant only in male mice, whereas females showed a significant reduction in the number of PV^+^ interneurons only in the PrL (fig. S10). The reduction of interneurons in the hippocampus of adolescent male mice only was consistent with the increased apoptosis of GABAergic neurons, which we observed as significant in male mice only (but see Mpfc), after acute treatment with JB1 (fig. S4, F and G). These changes further agree with a statistically significant reduction of tonic inhibition and sIPSCs (but not sEPSCs) in the hippocampus of males only, further confirming the possible causality link between loss of inhibition and increased excitability observed in pyramidal neurons from JB1-treated male mice (fig. S5).

These results indicate a sex-biased alteration of inhibitory circuits and behavioral outcomes upon IGF-1R inhibition during development, suggesting a potential link between electrophysiological and behavioral phenotypes in JB1-treated mice.

### Pharmacological treatment with an extrasynaptic GABA_A_R PAM rescues pyramidal neuron excitability and behavioral alterations in adolescent JB1 mice

Next, we evaluated whether decreased GABAergic tonic inhibition could be one of the possible mechanisms underlying the behavioral phenotypes of JB1-treated mice. We treated JB1 male mice with the drug GNX GNX is a PAM of GABA_A_Rs [in particular those containing delta subunits, (*38*)] known to increase GABAergic tonic currents by acting at extrasynaptic sites (*38*), and it is also an FDA-approved drug currently in clinical trials for other brain diseases (*39*).

**Fig. 5.**
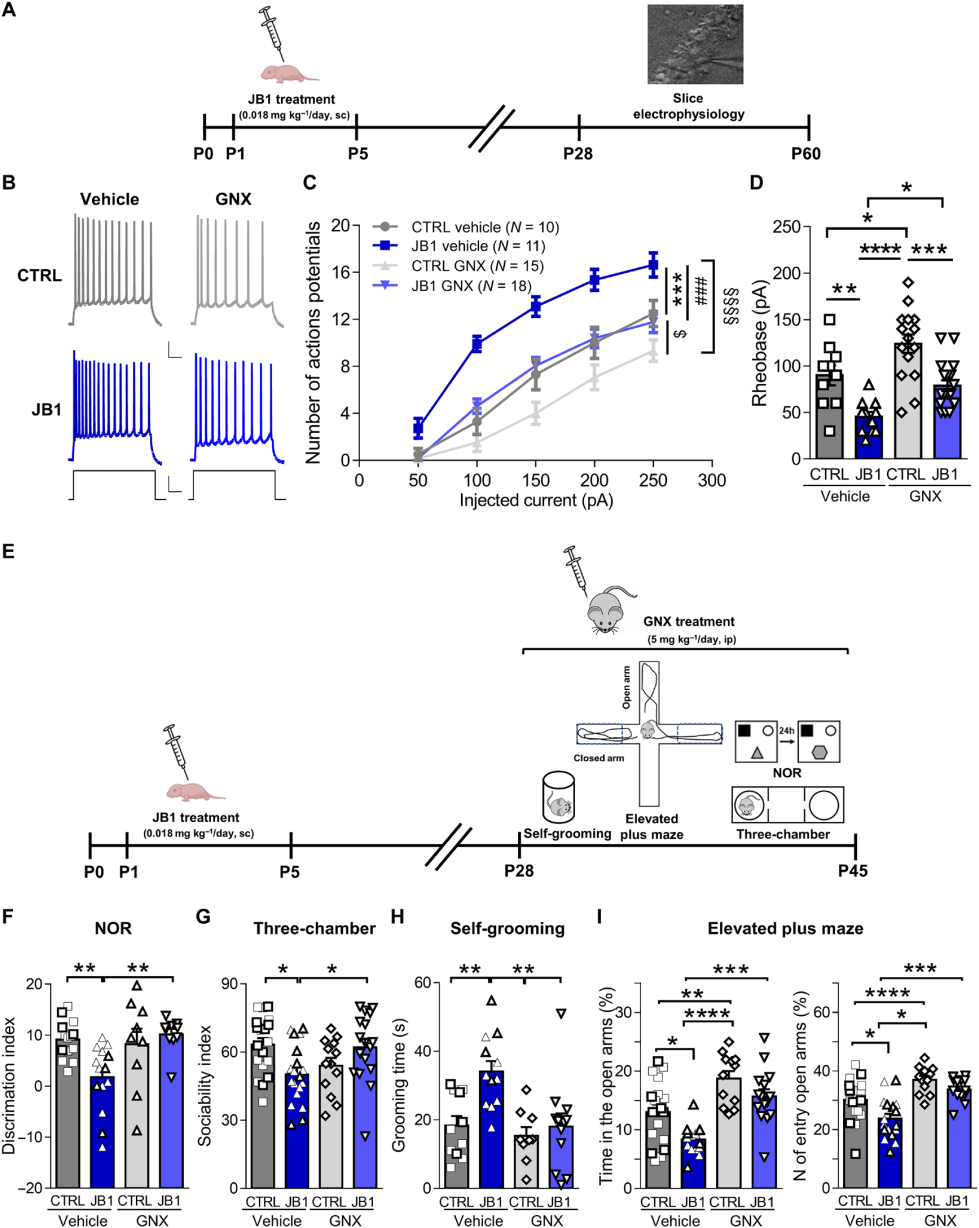
PAM of extrasynaptic GABA_A_Rs rescues pyramidal neuron excitability and behavioral alterations in adolescent JB1 mice. (**A**) Experimental protocol. (**B**) Current-clamp recordings of membrane potential changes in response to a 250-pA depolarizing current step in CA1-hippocampal pyramidal neurons in the presence of ganaxolone (GNX, 1 μm) or vehicle (DMSO) in the recording solution in brain slices from P>35 male mice. Scale bars, traces: 20 mV, 100 ms; stimuli: 150 pA, 100 ms. (**C**) Average action potential numbers (±SEM) elicited by depolarizing current steps. In parentheses: number of recorded cells from five CTRL and seven JB1 littermates (five litters). Two-way ANOVA RM, *F*_treatment_(3,50) = 16.10, *P* < 0.0001, Tukey’s post hoc test. CTRL-vehicle versus JB1-vehicle: ***P < 0.001; JB1-vehicle versus JB1-GNX: ###*P* < 0.001; CTRL-GNX versus JB1-vehicle: §§§§*P* < 0.0001; CTRL-GNX versus JB1-GNX: $*P* < 0.05. (**D**) Average Rheobase of all recorded cells from five CTRL and seven JB1-treated mice (five litters). Two-way ANOVA, *F*_treatment_(1,52) = 17.82, *P* < 0.0001, Tukey’s post hoc test, *P < 0.05, **P < 0.01, ***P < 0.001, ****P < 0.0001. (**E**) Experimental protocol. (**F** to **I**) Mean ± SEM and single-animal cases of the discrimination and sociability indexes in the NOR and three-chamber tests, the self-grooming time, the percentage of time spent in the open arms, and the percentage number of entries in the EPM in male mice previously treated with JB1 or vehicle (CTRL) as pups and treated as adolescents with ganaxolone (GNX) or vehicle. For all the panels, two-way ANOVA, Tukey’s post hoc test, *P < 0.05, **P < 0.01 ***P < 0.001, ****P < 0.0001. NOR: *F*_interaction_(1,43) = 7.059, *P* < 0.05. Three-chamber: *F*_interaction_(1,63) = 10.30, *P* < 0.01. Self-grooming: *F*_interaction_(1,40) = 8,774, *P* < 0.01. EPM: time, *F*_treatment_(1,56) = 31.72, *P* < 0.0001; entries, *F*_treatment_(1,57) = 28.97, *P* < 0.0001. Single data points with thinner borders represent data from the experiment in fig. S7C (F), fig. S7E (G), fig. S7G (H), and fig. S8, B and C (I). Schematic cartoons by BioRender.com.

First, we evaluated the ability of GNX to normalize the increased excitability of pyramidal neurons in acute hippocampal slices from JB1-treated males during adolescence/young adulthood (Fig. 5A). We found that bath application of GNX (1 μm) reduced the excitability of neurons from JB1-treated male mice to the levels of control littermates (Fig. 5B), as assessed by the number of fired APs (Fig. 5C) and rheobase values (Fig. 5D). Next, we evaluated the efficacy of in vivo GNX treatment [5 mg kg^−1^, intraperitoneally (ip), daily (*40*)] in ameliorating the poor behavioral outcomes of JB1-treated male mice. Three days after the first treatment with GNX at P28, we started performing all the behavioral tasks in which JB1-treated male mice performed significantly worse than controls (Fig. 5E). We found that GNX treatment rescued the poor novel discrimination ability of JB1-treated mice in the NOR test (Fig. 5F). Moreover, GNX improved the sociability of JB1-treated mice, occurring when exposed to a never-met intruder versus an object in the three-chamber test (Fig. 5G), and completely rescued their increased repetitive behavior in the grooming task (Fig. 5H). Notably, GNX treatment also ameliorated the anxiety phenotype observed in female mice (Fig. 5I). On the other hand, GNX treatment decreased anxiety in CTRL females as well (Fig. 5I), indicating that increasing tonic inhibition may be a generally effective approach to reduce anxiety behavior. Because some GABA_A_R PAM (i.e., benzodiazepines) could have negative effects on cognition with prolonged uses (*41*), we performed a NOR test in adult (P65 to P70) mice, which previously underwent a GNX treatment analogous to the one we performed in JB1 animals for the rescue experiments (5 mg kg^−1^, ip, daily from P28 to P45). We found no significant difference between GNX-treated and vehicle-treated mice (NOR discrimination index: mean CTRL vehicle: 13.42 ± 1.81, *N* = 7; mean GNX: 10.65 ± 2.418, *N* = 7; two-tailed Student’s *t* test, *t* = 0.92; *P* value = 0.38).

Finally, we evaluated the possibility that GNX rescue occurred by (also) improving myelination, as observed with other neurosteroids (*42*). However, we did not find any improvement of JB1-treated mice after GNX treatment (table S10).

These results demonstrate that restoring tonic current with a PAM of extrasynaptic GABA_A_R in adolescence rescues behavioral alterations caused by early IGF-1R inhibition in mice and suggest modulation of GABA_A_ tonic currents by GNX as a possible therapeutic intervention to ameliorate brain disorders in people who were born preterm.

### Preterm children with no diagnosis of neonatal brain lesion by magnetic resonance display cognitive deficits and autistic traits with a sex-dependent bias

Finally, we validated our mouse model of brain disorders of prematurity devoid of any type of brain lesion (very common in the past), with a parallel pilot neuropsychological evaluation in children born preterm nowadays (fig. S11A). In particular, we assessed the clinical profile of a cohort of severely preterm children born before 32 weeks of gestation carefully selected (fig. S11B) for being devoid of any brain lesion related to prematurity [even the minor that escape routine brain ultrasound and anatomical brain magnetic resonance diagnosis (*7*–*9*)], as we evaluated at their term-corrected age (see Materials and Methods for imaging details). The ad hoc neuropsychological evaluation, which we assembled based on existing tests, involved both cognitive and autistic trait assessments of the preterm children at their standard follow-up visit of 5 years of age (defined below as ex-preterm children; fig. S11A). As controls, we used data from the normative sample of the Wechsler Primary and Preschool Scale of Intelligence (WPPSI-IV) (*43*), the NEPSY-II (*44*), and the Autism Quotient for Children (AQ-C) (*45*), which report standardized scores derived from a large sample of typically developing children (TDC), as already performed in other independent studies (*46*). In our cohort of ex-preterm children, we observed a wide range of deficits distributed across the entire sample. In particular, we found that ex-preterm children showed, on average, global impairment in all neuropsychological domains evaluated by the WPPSI-IV, including the visuospatial index (VSI, an assessment of the ability to organize visual information), processing speed index (PSI, an assessment of the capacity to achieve and retrieve information due to selective attention and short reaction time), and working memory index (WMI; fig. S12, A to D).

Because we aimed to closely parallel our results in mice to the clinical data in ex-preterm children, we reasoned that some behavioral subtests included in the WPPSI-IV and NEPSY-II were fairly analogous to those we conducted in mice. In particular, we specifically analyzed the results of the zoo locations test (subtest of the WMI), which assesses short-term memory, as a correlation of the T-maze test in mice; the memory design delayed test (a subtest of the NEPSY-II), which assesses long-term visuospatial memory, as a correlation of the NOR (content score) and OL (spatial score) tests in mice; and the AQ-C, a parent questionnaire used to evaluate social and repetitive behaviors, as a correlation of the three-chamber and self-grooming tests (fig. S11C). We found that ex-preterm children performed significantly worse than standard norms in the zoo locations test (fig. S11D), similar to what we observed in the T-maze task in our mouse model. Moreover, ex-preterm children showed a significantly lower score compared to the standard norms in the memory design delayed task (fig. S11E), as we observed in the NOR/OL tests in mice. Furthermore, ex-preterm children showed a significantly higher score than TDC children on the AQ-C questionnaire (fig. S11F), similar to what we observed in our mouse model in the three-chamber and self-grooming tests. Finally, we segregated our results in ex-preterm children by sex, and we found that both males and females were significantly affected in all the performed tests (fig. S12, E to I).

Although we found an average decrease in cognitive and behavioral performance in our cohort of ex-preterm children, this per se does not indicate the need for a clinical intervention. The behavioral performances for each child could still fall within a physiological range. Thus, we specifically identified the percentage of children who would need clinical interventions by assessing the number of those showing a pathological score, as defined by WPPSI-IV and NEPSY-II normal ranges (standard norm) or the AQ-C cutoff for ASD (*43*–*45*, *47*). We found that there was a significantly higher percentage of ex-preterm males (but not females) performing in the pathological range [score < 70 (*43*, *47*)] compared to the expected percentage in the TDC population (second percentile rank), when we considered the general index of the WMI. This is in agreement with the sex bias we described in our mouse study. The higher percentage of ex-preterm children with a pathological score in the VSI and PSI did not reach statistical significance (fig. S12, J to L). However, when we analyzed the zoo locations subtest of the WMI, we found a significantly higher number of both male and female ex-preterm children with pathological scores [score ≤ 6 (*43*, *47*)] in comparison to the percentage expected from the standard population (10th percentile rank; fig. S11G). Notably, males were significantly more affected than females, in agreement with our mouse data in the T-maze. Moreover, we found a significantly higher percentage of ex-preterm children performing in the pathological range in the memory design delayed task (score ≤ fifth percentile rank) compared to the expected percentage in the TDC population [fifth percentile rank (*44*)], with similar results in males and females (fig. S11H). Finally, we found a significantly higher number of ex-preterm children with a score above the autism diagnosis cutoff [score ≥ 76 (*45*)] assessed by the AQ-C parent questionnaire compared to the expected percentage in the TDC population [4% (*45*); fig. S11I]. Again, when we segregated the ex-preterm population according to sex, we found that males (but not females) had a significantly higher percentage above the autism spectrum cutoff than the TDC population [7% in males and 2% in females (*45*)]. This is in agreement with the results obtained in our mouse model with the three-chamber and self-grooming tests.

Although our pilot study has a small sample-size and needs to be complemented with larger future clinical studies, these results demonstrate that ex-preterm children, even with no magnetic resonance–diagnosed brain lesion, exhibit impairment in cognitive and social skills, with males representing a higher percentage of children with pathological scoring in short-term memory assessment and autistic traits, in agreement with what we found in our mouse model.

## DISCUSSION

One to five percent of all babies are born severely preterm (*1*). In the last three decades, major therapeutic advances in neonatal care units have exponentially raised the survival rate of preterm newborns at increasingly younger ages. Nowadays, prenatal corticosteroid and surfactant administration, and upgraded protocols for ventilation have tremendously decreased the occurrence of hypoxic damage and therefore the incidence of severe neurological diseases, such as cerebral palsy, and blindness in babies born severely premature (*48*). However, the concomitant increased incidence of diagnosed neuropsychiatric diseases (25 of 50% of preterm babies born at <32 weeks of gestational age) in the preterm population in recent decades [even when considering the significant reduction of necrotic lesions and neuroinflammation in preterm newborns (*48*)] suggests that people born preterm are vulnerable per se [and independent of severe birth complications (*49*)] to developing neurodevelopmental disorders.

Current mouse models of preterm brain disorders [hypoxic or inflammatory challenges in neonatal rodents (*50*)] have tremendously advanced our knowledge about the effect of hypoxia or inflammation on motor and cognitive deficits in the developing brain, which were common birth complications in the past. However, no experimental mouse model was designed to mimic the brain and behavioral abnormalities of the population of ex-preterm babies born currently with fewer and fewer birth complications and no brain lesions, who also display social deficits and repetitive behaviors, together with cognitive impairment (*4*). Here, we report the development of a mouse model of preterm brain disorders that recapitulates behavioral, functional, and anatomical microstructural alterations in preterm children born with no brain lesion. We used our mouse model to understand the neuronal basis of its brain pathology and design a possible therapeutic approach to rescue its behavioral abnormalities, which closely paralleled those of the modern cohort of ex-preterm children described here.

To create the mouse model, we switched the focus of investigation from the birth complications of preterm newborns to the biology of the developmental challenges that the brains of preterm newborns have to face. In particular, we focused on the possibility that the behavioral abnormalities in the preterm population may derive from the lack of exposure to IGF-1, a placental growth factor present in the maternal uterus environment (*12*). Although our mouse model was designed to adhere to the need to find a model for the new population of preterm babies with no brain lesion, it is also inclusive (at least in part) of the population of preterm newborns with brain lesions. All preterm babies are prematurely separated from their mother placenta, and they do not receive the same hormonal influence as their born-at-term counterparts during development, independent of the cause of their preterm delivery. Among the many placental factors possibly involved in the poor brain development of preterm babies, we focused on IGF-1 for several reasons: (i) IGF-1 is a main regulator of brain development and plasticity (*10*, *21*); (ii) IGF-1 serum levels are reduced in preterm newborns, and low IGF-1 levels correlate with prematurity-related complications (*15*–*17*); (iii) based on the latter findings, a recent clinical trial (NCT01096784) investigated recombinant human IGF-1 supplementation in preterm newborns. This study did not reach the primary end point on prevention of ROP, but it showed promising effects in protecting preterm newborns from bronchopulmonary dysplasia and intraventricular hemorrhage, becoming a starting point for future trials (NCT02386839 and NCT03253263). Of note, although the mechanism behind IGF-1 reduction in preterm newborns are not fully understood (e.g., no placental contribution, poor nutrition, reduced fetal insulin levels, and exposure to inflammation), our mouse model mimics IGF-1 signaling reduction of premature infants independently of its cause.

The transient inhibition of IGF-1R during the period of mouse development corresponding to the third trimester of pregnancy in humans was sufficient to recapitulate impaired (sex-biased) anatomical, functional, and behavioral deficits present in the current preterm population, indicating the strong face validity of our model. The choice of our treatment period reflected the reported IGF-1 deficiency of preterm newborns (*12*) and was based on the corresponding mouse developmental stage (*18*). Acute IGF-1R blockade influenced the phospho-proteome of P5 mice by modifying the phosphorylation state of proteins downstream of the IGF-1 kinase signaling pathway. In particular, it led to changes associated with neuronal and RNA processes, and apoptosis, in line with the long-term effect that we observed on neurons later in life (i.e., electrophysiological parameters and reduced number of interneurons). Key proteins associated with neuropsychiatric disorders (i.e., ID and ASD) were also changed after early, acute JB1 treatment, consistent with behavioral deficits that we detected in JB1-treated mice when adolescents and with the frequent cognitive and social sequelae of preterm newborns (*4*). Although with a lower *P* value, genes associated with neurodevelopmental disorder, ADHD, epilepsy, schizophrenia, and bipolar disorder were also enriched in our dataset, in line with the possible comorbid neuropsychiatric disorders at times reported in people born preterm. During adolescence, the brains of our mouse model also showed hypomyelination, a condition observed in preterm newborns (*1*) and suggested to be among the causes of several other neurological and behavioral disabilities (*1*). Our mouse model similarly reproduced a reduced number of interneurons in the cortex, which is also observed in preterm newborns (*27*, *51*) and frequently associated with psychiatric disorders (*52*). Furthermore, EEG recordings in awake head-fixed mice (similar to the resting state in humans) uncovered a decreased high-frequency power–to–low-frequency power ratio, previously associated with immature neuronal circuits (*53*). This is in agreement with previous reports in nonlesioned young adults born preterm (*29*). Moreover, the long-lasting effects on behavior in our early IGF-1R inhibited mice led to deficits in social communication, reduced perception of thermal stimuli, and insecure mother-attachment behavior, typical of children born severely preterm (*54*–*56*). Furthermore, our mouse model showed cognitive impairment, reduced sociability and increased repetitive behaviors during adolescence, again in agreement with what was found in ex-preterm adolescents (*4*, *57*).

The data highlighted above provide strong evidence and corroborate the existing literature on the key role of IGF-1 signaling in sculpting neuronal circuit and behavior during development. Several laboratories reported already successful rescue—upon IGF-1 treatment—of cellular and behavioral alterations in mouse models of autism (*58*) and neurons derived from induced pluripotent stem cells from people with neurodevelopmental disorders (*59*, *60*). On the other hand, we here performed an extensive cellular, electrophysiological (ex vivo and in vivo), and behavioral characterization also of *wild type* mice upon reduction of IGF-1 signaling. This kind of approach had been precluded in IGF-1 KO mice, mostly due to their high perinatal mortality (only <5% of pups survive). Anyhow, the survivors of IGF-1-KO mice do share immunopathological features (e.g., reduced interneuron number and hypomyelination) in agreement with our mouse model (*61*). Furthermore, our data are in line with the mild cognitive and possibly social impairment reported in people with heterozygous mutations of IGF-1R (*62*), and recent data in preterm born piglets (which have lower serum IGF-1 levels, similarly to humans) treated with IGF-1 supplementation, which leads to rescue of structural/motor impairment (*63*, *64*).

Finally, our mouse model allowed us to dig deep into the molecular mechanisms underlying its behavioral abnormalities by electrophysiological recordings. These results indicated excessive excitability of neurons due to decreased GABAergic tonic inhibition in JB1-treated mice. Accordingly, when we segregated our results by sex, we found that male mice were more affected than females at the cellular, molecular, and behavioral levels. This is a feature also described in other neurodevelopmental disorders (*65*), especially in the autism spectrum (both clinical data and animal models) and including the severe preterm population [only clinical data (*36*)]. The sex bias that we found in GABAergic inhibition, phosphoproteome, and behavioral alterations indicated a possible neuroprotective mechanism in females, conferring them protection against interneuron loss and a higher threshold in developing both cognitive and social difficulties compared to males.

As a possible mechanism involved in the differences between male and female JB1-treated mice in terms of interneuron number, tonic inhibition, and cognitive impairment, both the Reactome pathway analysis and the immunostaining experiments indicate apoptosis of inhibitory neurons, which we found significantly increased in males only. These data, together with the known role of female neurosteroids in increasing tonic GABAergic signaling (*66*), support our hypothesis on the critical role of tonic inhibition in the sex bias that we found in our mouse model and possibly in the sex-specific behavioral outcomes in ex-preterm people (*4*). Finally, the sex bias that we found is also in line with results from a recent study showing that a reduction in allopregnanolone during pregnancy leads to core symptoms related to autism only in male offspring (*67*), with females mostly unaffected at the behavioral level (*67*). Female mice were not totally immune to the effect of IGF-1R blockade during development under our experimental conditions. They displayed increased anxiety behavior, consistent with literature data indicating that females are generally protected against preterm brain-related complications but with some emotional/anxiety difficulties (*57*).

We also probed the face validity of our mouse model by investigating the clinical profile of a small cohort of ex-preterm children completely devoid of even minor brain lesions. The data that we collected in our pilot study indicated a global decrease in cognitive performance as well as an increased tendency toward autistic traits, in agreement with the literature (*4*). Furthermore, we selected for further analysis in our small cohort of patients an ad hoc set of behavioral subtests guided by our mouse data. This also demonstrated the predictive validity of our mouse model. In particular, we selected tests that we considered as close as possible to those we used in mice. Moreover, in our human study, we tried to stay as close as possible to clinical practice, and we set up a data analysis approach based on pathological threshold values (indicated in the literature), which allowed us to compute the number of children who would potentially need clinical intervention. As predicted by our mouse model, we found short-term memory to be the general brain function most often altered in ex-preterm newborns, with males more affected than females. Moreover, our translational approach allowed us to identify the zoo locations test as a quick and effective task to detect ex-preterm children with altered spatial memory abilities. Similar to the NOR and OL tests in mice, we also found impaired long-term visuospatial memory in the memory design delayed task in our small cohort of preterm children. In this case, we did not find any difference between males and females, as we also observed in the OL test in mice (but also see the NOR results). Last, as predicted by our mouse data on the three-chamber and self-grooming tasks, we found that a higher number of ex-preterm male children scored above the autism diagnosis cutoff. Although this was a pilot study with a small sample size, the parallel behavioral batteries and data analysis that we assembled here to closely compare mouse and clinical data will possibly aid other laboratories in investigating the biological processes underlying brain disorders of prematurity or other neurodevelopmental disorders characterized by similar core symptoms.

The lack of updated animal models and the consequent knowledge on the molecular underpinnings of brain disorders of prematurity has hindered the development of pharmacological treatments specifically designed to ameliorate behavioral outcomes in severely preterm babies. Through our mouse model, we identified decreased tonic inhibition as a potential therapeutic target for preterm brain disorders and indicated treatment with the neurosteroid derivative GNX (*38*) as a potential therapeutic approach able to rescue neuronal excitability, cognitive, social, and repetitive behavior in male adolescents. This evidence supports the growing concept in the field of neurodevelopmental disorders, indicating that (at least some) clinical symptoms can still be rescued by pharmacological treatments at late stages of development and adulthood (*68*). GNX treatment was sufficient to reduce the anxiety levels in adolescent female mice, suggesting that the slight reduction in GABAergic inhibition observed in females may be sufficient to trigger anxiety in the female brain. This is in agreement with the literature indicating an association between GABA tonic inhibition and anxiety/depression in females (*39*, *66*). Of note, GNX has been recently proposed as a hormone-replacement therapy for hyperactivity by an unknown mechanism of action in a guinea pig model of premature birth with brain injury (*69*, *70*). The treatment was proposed at very early stages of the neurodevelopment (corresponding to fetal stages in humans), which lead to increased pup mortality and uncertain outcomes on brain development. We propose here a well-grounded rationale for a safe usage of GNX in the adolescent population of ex-premature babies to treat diverse core behavioral abnormalities. The usage of GNX in children/adolescents has been recently approved for cyclin-dependent kinase-like 5 (CDKL5) deficiency (*39*), a disease characterized by epilepsy and intellectual and motor disabilities. Moreover, GNX is in clinical trials for the treatment of the pediatric/adolescent population with neurodevelopmental disorders such as Fragile X syndrome and Protocadherin 19 syndrome, but also of adolescents/adults with postpartum depression (*39*). Thus, GNX could be readily repurposed as a therapeutic option for adolescents with preterm brain disorders in phase 2 clinical trials in the near future. Finally, GNX has been also proposed to rescue core behaviors in a mouse model of ASD during adolescence (*71*).

In conclusion, although neurodevelopmental disorders characterizing preterm people are due to multiple factors, our mouse study indicates a causal relationship between reduced IGF-1 signaling during a critical period of brain development and aspects of preterm brain disorders similar to those we observed in a pilot study with a small cohort of ex-preterm children devoid of neonatal brain lesions, supporting the rationale for IGF-1 supplementation in preterm newborns. Moreover, our phospho-proteomic dataset also contained several key proteins involved in ASD, ID, and other brain disorders. Thus, our dataset holds promising potential to be used as a future resource by other laboratories worldwide for investigating the relationship between IGF-1 and neurodevelopmental disorders. This is even more relevant when considering the growing evidence linking IGF-1 to such conditions (*58*, *60*, *72*). Furthermore, our data suggest patient stratification (possibly based on IGF-1 levels together with absence of brain lesions) in future clinical trials with recombinant human IGF-1 (and analogous drugs) for the treatment of preterm-related brain disorders. Preterm newborns are a very heterogeneous population (*6*). Moreover, the successful story of trofinetide (*72*, *73*), an analogous IGF-1 small molecule that became the first drug to receive the FDA approval to improve sociability and cognition in a neurodevelopmental disorder (Rett syndrome), suggests that this drug could also be considered for the treatment of preterm newborns. Finally, our translational data in the easy-to-implement and reliable mouse model of preterm brain disorders that we developed here also indicate repurposing of GNX as a potential novel therapeutic approach to be tested for efficacy in future clinical trials on the treatment of brain disorders of prematurity during adolescence.

### Limitations of the study

Although our pilot clinical study shows that preterm children without any brain lesion at birth exhibited cognitive and social deficits, caution should be exercised when interpreting the data due to the small sample size. Although we carefully selected our clinical cohort based on stringent criteria (clinical flowchart, fig. S11B and Materials and Methods), preterm children without brain lesions remain a very heterogeneous population and our pilot study needs to be replicated in a larger cohort of patients and in multiple hospitals.

## MATERIALS AND METHODS

### Experimental design

We explored the possibility that reduced IGF-1 levels previously reported in preterm newborns could be mechanistically linked to the development of brain disorders. The developed mouse model that recapitulates reduced IGF-1 signaling during the third trimester of human pregnancy uncovered mechanisms behind associated behavioral and brain alterations. This enabled us to design a therapeutic strategy. Furthermore, we complemented our mouse study with human data from preterm children.

### Animals and treatments

All animal procedures were approved by Istituto Italiano di Tecnologia (IIT) licensing in compliance with the Italian Ministry of Health (D.Lgs 26/2014; authority granting ethics approval n° 339/2021-PR) and EU guidelines (Directive 2010/63/EU). A veterinarian was employed to maintain the health and comfort of the animals. Mice were housed in filtered cages in a temperature-controlled room with a 12-hour dark/12-hour light cycle and with ad libitum access to water and food. All efforts were made to minimize animal suffering and to use the lowest possible number of animals required to produce statistically relevant results, according to the “3Rs concept.” Here, we used C57BL/6J mice (Charles River). For the experiment aiming at the count of SST interneurons, we used SST cre homozygous mice (the Jackson Laboratory, #013044) crossed with TdTom homozygous mice (the Jackson Laboratory, #007908). Offspring aged between P6 and P60 were used for the experiments. Both males and females were used for all experiments, except for experiments related to Fig. 5 (A to H) (only males) and Fig. 5I (only females). C57BL/6J mice were randomly assigned to vehicle (saline, CTRL) or JB1 (0.018 mg kg^−1^ body weight) (*19*) groups, and treated daily from P1 to P5 by subcutaneous injection. For the rescue experiment, CTRL mice or JB1-treated mice were randomly assigned to GNX (Sigma, 5 mg kg^−1^, in 20% 2-hydroxypropoyl-β-cyclodextrin) or vehicle [20% 2-hydroxypropoyl-β-cyclodextrin, 3% dimethyl sulfoxide (DMSO)] and treated from P28 to P45 intraperitoneally. Animals were tested for the first time after 3 days of treatment. On the day of the behavioral testing, the injection was performed 1 hour before the task began. Polymerase chain reaction (PCR) was used to genotype SST-TdTom mice and for the establishment of sex in mouse pups, as previously described (*74*, *75*).

### Enzyme-linked immunosorbent assay

Hippocampal brain and blood samples were collected at P5 from JB1- and control (vehicle)–treated littermates 1 hour after the last treatment on the last day of treatment. Hippocampal samples were rapidly collected in ice-cold phosphate-buffered saline (PBS) and were lysed in ice-cold radioimmunoprecipitation assay (RIPA) buffer (1% NP-40, 0.5% deoxycholic acid, 0.1% SDS, 150 mM NaCl, 1 mM EDTA, and 50 mM tris, pH 7.4) containing 1 mM phenylmethylsulfonyl fluoride (PMSF), 10 mM NaF, 2 mM sodium orthovanadate, and 1% (v/v) protease and phosphatase inhibitor cocktails (#P5726 and #P0044, Sigma). Samples were clarified through centrifugation at 20,000*g* at 4°C, and the protein concentration was determined using the BCA kit (Pierce). Blood samples were collected in EDTA (500 mM)–coated Eppendorf and plasma was isolated by centrifuging at 10,000*g* at 4°C. Equal amounts of plasma and hippocampal lysates were loaded in the ELISA plate of the IGF-1 mouse ELISA kit (#RAB0229, Sigma), and the assay was performed following the manufacturer’s instruction.

### Plasma proteomic and hippocampal phospho-proteomic

Plasma and hippocampal brain samples were collected at P5 from JB1- and control (vehicle)–treated littermates 1 hour after the last treatment on the last day of treatment. Samples were processed as for the ELISA assay and/or phosphoproteome analysis. Plasma samples (5 μl) were lysed, reduced, and alkylated with 50 μl of Preomics lysis buffer. To obtain about 50 μg of protein material, 7 μl of lysed sample was diluted in 25 mM tris-HCl. For phosphoproteomics of the mouse brains, we used 200 μg of samples lysed in RIPA buffer. Protein digestion of plasma and brain samples was automated on a KingFisher Apex robot (Thermo Fisher Scientific) in 96-well format. The tip plate was stored in plate #1. Lysate samples were stored in plate #2, in a final concentration of 70% acetonitrile and with magnetic beads in a protein/bead ratio of 1:4 (1:1 SpeedBead Magnetic Carboxylate, 45152105050250 and 65152105050250). Washing solutions were in plate #3 to #5 (acetonitrile), plate #6 (70% ethanol), and plate #7 (isopropanol). Plate #8 contained 100 μl of digestion solution of 25 mM tris-HCl, pH 8, LysC (Wako) in an enzyme/protein ratio of 1:400 (w/w), and trypsin (Promega) in an enzyme:protein ratio of 1:200. The protein aggregation was carried out in two steps of 1 min mixing at medium mixing speed, followed by a 10-min pause each. The sequential washes were performed for 2.5 min at slow speed, without releasing the beads from the magnet. The digestion was set to 2.5 hours at 37°C and slow speed.

Phosphopeptide enrichment of brain samples was carried out on the same KingFisher Apex robot in 96-well format. Digested peptides were doubled in volume with phosphopeptide enrichment loading buffer [80% Acetonitrile (ACN), 5% trifluoroacetic acid (TFA), and 0.1 M Glycolic Acid (GA)] and stored in plate #4. The tip plate was stored in plate #1, 40 μl of Zr-IMAC HP beads (ReSyn Biosciences) in 100% ACN in plate #2, and loading buffer for beads wash in plate #3. Plate #5 to #7 were filled with 500 μl of washing solutions: loading buffer, 80% ACN, 1% TFA, and 10% ACN, 0.2% TFA, respectively. Plate #8 contained 200 μl of 1% ammonia for elution. The beads were washed in a loading buffer for 5 min at medium mixing speed, followed by binding of the phosphopeptides for 20 min at medium speed. The sequential washes were performed for 2 min at fast speed. Phosphopeptides were eluted for 10 min at medium mixing speed. The eluate was acidified with TFA and loaded directly on EvoTips.

Phosphopeptides were analyzed on the Evosep One system using an EASY spray PepMap Neo column (75 μm × 15 cm, Thermo Scientific) and the preprogrammed Whisper gradient of 20 samples per day, with a flow rate of 100 nl/min. The column temperature was maintained at 55°C and interfaced online with the Orbitrap Exploris 480 MS (Thermo Scientific) with FAIMS Pro Duo Interface (Thermo Scientific). MS analysis was performed in DIA mode. FAIMS CV was set to −50 at standard resolution. Full mass spectrography resolution was set to 120,000 in a range between 375 and 1500 mass/charge ratio (*m*/*z*) and with an AGC target of 300% with a maximum IT set to Auto. AGC target value for fragment spectra was set at 1000%. Forty windows of 15 Da were used with an overlap of 1 Da. Resolution was set to 30,000 and IT was set to Auto. Normalized collision energy was set at 30%. All data were acquired in profile mode using positive polarity.

Plasma peptides were analyzed on the Evosep One system using an EASY spray column (150 μm × 15 cm, Thermo Scientific) and the preprogrammed gradient of 30 samples per day, with a flow rate of 500 nl/min. The column temperature was maintained at 40°C and interfaced online with the Orbitrap Exploris 480 MS (Thermo Scientific) with FAIMS Pro Duo Interface (Thermo Scientific). MS analysis was performed in DIA mode. FAIMS CV was set to −45 at standard resolution. Full MS resolution was set to 120,000 in a range between 375 and 1500 *m*/*z* and with an AGC target of 300% with a maximum IT set to Auto. AGC target value for fragment spectra was set at 1000%. Forty windows of 15 Da were used with an overlap of 1 Da. Resolution was set to 30,000 and IT was set to Auto. Normalized collision energy was set at 30%. All data were acquired in profile mode using positive polarity.

All DIA raw files were processed with Spectronaut version 18 (*76*) using a library-free approach (directDIA) with Phospho PTM Workflow for phospho-proteomics of brain samples and default settings for proteomics of plasma samples. Enzymes/Cleavage Rules were set to Trypsin/P, LysC. Library was generated against the UniProt Mouse database (release UP000000589_ 10090 July 2021). Carbamidomethylation was selected as a fixed modification, methionine oxidation and N-terminal acetylation were selected as variable modifications for both proteomics and phospho-proteomics, while Deamidation (NQ) was selected as variable modification for proteomics and Phospho (STY) was selected as variable modification for phospho-proteomics. FDRs of PSMs and peptide/protein groups were set to 0.01. For quantification Precursor, Filtering was set to Identified (Qvalue) and MS2 was chosen as quantity MS-level.

### Behavioral testing

CTRL and JB1-treated mice (P6 to P45) were tested over a total period of 39 days. Mouse pups behaviorally assessed preweaning performed only the USV, hot plate, or mouse strange situation test. Mouse assessed after weaning performed a battery of tests over a total period of 5 to 17 days. The behavioral tests were performed from the least to the most stressful test, with order and modalities detailed in table S11. The tasks were video-recorded and then analyzed manually by a blind operator, unless otherwise indicated. After each trial or experiment, the diverse apparatuses and objects were cleaned with 40% (pups) or 70% ethanol (adolescent). Mice were habituated to the room 1 hour before the test.

#### Ultrasonic vocalizations

Each pup was separated at P6 from the mother and littermates and placed in an empty container (diameter, 5 cm; height, 3 cm), located in a sound-attenuating styrofoam box (diameter, 30 cm; height, 40 cm). Calls were recorded for 5 min by an ultrasound microphone sensitive to frequencies of 10 to 180 kHz (Avisoft UltraSoundGate condenser microphone capsule CM16, Avisoft Bioacoustics) and Avisoft Recorder software (Version 3.2). Data analysis was performed using Avisoft SASLab Pro (Version 4.40). The total number of calls during the 5-min recording session was quantified. Waveform patterns of calls were examined across 50 diverse sonograms and classified in 10 categories of call types (*77*). Only pups that emitted at least 50 calls were used for the evaluation, as previously described (*25*).

#### Hot plate

Response to an acute thermal stimulus was measured in pups at P9 using an adapted hot plate test from a previously described protocol (*78*). In particular, the experimenter held the pup between the thumb and forefinger in an upright position and gently placed the hind paws of the mouse on the surface of the hot plate kept at constant temperature of 55°C. The latency to withdraw the paws from the hot plate was measured. To prevent any heat injury to pups, a cutoff latency of 30 s was applied. Mice that did not show any response were removed after 30 s to prevent any thermal injury, and they were classified as nonresponsive mice.

#### Mouse strange situation

Assessment of the attachment behavior of each pup (18-day-old) to their mother was performed as previously described (*26*). First, in 15 min of pretest, the mother (primiparae) and a stranger (a virgin, sex-, age-, and strain-matched mouse) became familiar in an open field arena (44 × 44 cm) evenly illuminated by overhead red lighting (12 to 14 lux). The test involved three episodes of 3 min each. During episode 1, one randomly chosen pup (the littermates remained in the home cage) was placed in the open field arena with the mother and the stranger. In episode 2, the mother was removed from the arena (reunited with her litter) and the pup was left alone with the stranger. In the third and final episode, the mother was returned to the arena containing the pup and the stranger. To evaluate attachment behavior of the pup to the mother, the time spent by the pup actively (i.e., head orientation and sniffing) interacting with the mother or with the stranger was scored in the diverse test episodes.

The following indexes were utilized for data analysis:

(i) Stranger preference index (SPI): the time spent with the stranger during episode 1 (S1) over the time spent with the mother during episode 1 (M1); SPI = S1/M1.

(ii) Stranger effect index (SEI): the time spent with the stranger during episode 2 (S2) over the average time spent with the stranger during episode 1 (S1) and during episode 3 (S3); SEI = S2/[(S1 + S3)/S2].

(iii) Maternal preference index (MPI): the time spent with the mother during Episode 3 (M3) over the time spent with the stranger during Episode 3 (S3); MPI = M3/S3.

(iv) Reunion index (RI): the time spent with the mother during episode 3 (M3) over the time spent with the mother during episode 1 (M1). RI = M3/M1.

#### T-maze

The T-maze is a black opaque plastic apparatus with a starting arm and two perpendicular goal arms, each equipped with a sliding door and evenly illuminated by overhead red lighting (12 to 14 lux). The T-maze test (spontaneous alteration protocol, 11 trials) evaluates short-term memory by analyzing the correct choice of the unexplored arm. The test was performed similarly to that previously described (*31*). In each trial, a mouse was first placed in the starting chamber for 20 s. Then, the sliding door was removed, and the animal was free to explore the apparatus. When the mouse entered (with all four limbs) one of the two goal arms, the opposite arm was closed with the sliding door. When the mouse (free to explore the remaining part of the apparatus) returned to the starting area, the previously closed goal arm was opened. The trial was repeated 11 times. Entry into a goal arm opposite the one previously chosen was considered a correct choice, while entry into the previously explored arm was considered an incorrect choice. Alternation score was calculated as the percentage of correct choices (i.e., left-right or right-left) over the total number of the 10 possible alternations.

#### Novel object recognition

The test evaluates long-term object recognition memory by measuring the ability of mice to recognize a new object with respect to familiar objects. The test was performed in a gray acrylic arena (44 × 44 cm), evenly illuminated by overhead red lighting (12 to 14 lux). On day 1, mice were habituated to the arena by freely exploring the chamber for 15 min. On day 2, during the acquisition phase, mice were free to explore three different objects (different in color, size, shape, and material) for 15 min. After 24 hours, one object from the acquisition phase was replaced with a novel object, and the mice were tested for 15 min for their ability to recognize the new object. The time spent exploring each object was defined as the number of seconds during which mice showed investigative behavior (i.e., head orientation and sniffing occurring within <1.0 cm) or clear contact between the object and the nose. The time spent exploring each object, expressed as a percentage of the total exploration time, was measured for each trial. The discrimination index was calculated as the difference between the percentages of time spent investigating the novel object and investigating the familiar objects: discrimination index = (novel object exploration time/total exploration time × 100) − (familiar object exploration time/total exploration time × 100). As a control, we monitored object preference during the acquisition phase and exploration time in the acquisition phase and trial phase. No differences were observed among any of the experimental compared groups (tables S12 and S13; table S16 for the rescue experiment).

#### Object location test

The test evaluates the spatial memory by measuring the ability of mice to recognize the new location of a familiar object. The test was performed in a gray acrylic arena (44 × 44 cm), evenly illuminated by overhead red lighting (12 to 14 lux). Mice were first habituated to the chamber for 15 min on day 1. On day 2, during the acquisition phase, mice were exposed to two identical objects for 15 min. After 24 hours, one of the two objects was moved during the test session to a novel location, and the mice were tested for 15 min for their ability to recognize the new location of the object. The time spent exploring each object was defined as the number of seconds during which mice showed investigative behavior (i.e., head orientation and sniffing occurring within <1.0 cm) or clear contact between the mouse and the object. A discrimination index was calculated as the percentage of time spent investigating the object in the new location minus the percentage of time spent investigating the object in the old location [discrimination index = (new object location exploration time/total exploration time × 100) − (old object location exploration time/total exploration time × 100]. As a control, we monitored object preference during the acquisition phase and the exploration time in the acquisition phase and trial phase (tables S14 and S15). Of note, during the acquisition phase (table S14), we observed a statistically significant difference between CTRL and JB1-treated mice in object preference. Nevertheless, the objects were randomly moved in the new location for the test phase, thus not affecting the results of the discrimination index in the test.

#### Three-chamber test

The test evaluates the social approach of the tested mouse versus a never-met intruder in comparison to an object (sociability) or versus a novel never-met intruder in comparison to the already-met intruder (social novelty). It was performed in a similar way to that previously described for mouse models of ASD (*31*). The three-chamber apparatus comprises a rectangle, three-chambered box of gray acrylic, evenly illuminated by overhead red lighting (12 to 14 lux). The chambers are accessible by rectangle openings with sliding doors. In the first 10 min (habituation), the tested mouse was free to explore the apparatus of two inverted stainless-steel wire pencil cups (one in each of the two side chambers), with a weighted plastic cup on top to prevent the mouse climbing on the top. Then, the tested mouse was briefly confined in the center chamber, while a never-met intruder (previously habituated to the apparatus) was placed in one of the side chambers, under the pencil cup. For the following 10 min (sociability test), the tested mouse was allowed to explore all three chambers. Then, the tested mouse was again briefly confined in the center chamber, while a novel never-met intruder (previously habituated to the apparatus) was placed in the other side chamber under the pencil cup. For the following 10 min (social novelty test), the tested mouse was allowed to explore all the three chambers. The time spent exploring the object or the intruder was calculated by measuring the number of seconds when the mice showed investigative behavior (i.e., head orientation and sniffing occurring within <1.0 cm). The sociability index was calculated as the difference between the time spent investigating the never-met intruder and the time spent investigating the familiar object divided by the total exploration time: sociability index = (never-met intruder exploration time − object exploration time)/(never-met intruder exploration time + object exploration time). The social novelty index was calculated as the difference between the time spent investigating the never-met intruder and the time spent investigating the already-met intruder divided by the total exploration time: social novelty index = (never-met intruder exploration time − already-met intruder exploration time)/(never-met intruder exploration time + already-met intruder exploration time).

#### Self-grooming

The test evaluates repetitive behavior in mouse grooming of all body parts. It was performed as previously described in autistic mice (*31*). Self-grooming was defined as licking or scratching the head or body parts with any of the forelimbs. Briefly, mice were placed individually into a clear plexiglass cylinder (30 cm high, 10 cm wide). After 5 min of habituation, the mouse was scored for 5 min for the time spent self-grooming in all body regions.

#### Open field

Open field data were extracted from the first day (habituation session) of either the NOR or the OL tests. In particular, general locomotor parameters such as average speed, distance traveled, and time immobile were extracted with the AnyMaze software (Stoelting Co.) during the whole 15 min of the test.

#### Elevated plus maze

The EPM test evaluates anxiety behavior, and it was performed as previously described (*79*). The maze consists of an apparatus containing two open and two black, closed arms (30 × 5 cm) with a small platform (5 × 5) in the center, evenly illuminated by overhead red lighting (12 to 14 lux). The apparatus is elevated 40 cm from the ground. After 1 hour of habituation in the room, each mouse was placed in the central part of the maze, with the head oriented toward the open arm, and allowed to freely explore the apparatus for 5 min. The number of entries and the time spent in the open arms were scored offline and expressed as a percentage of the total number of arm entries (for number of entries) or test duration (for time spent in the open arms).

For behavioral experiments, we adopted the following exclusion criteria independent of treatment (before blind code was broken). In the T-maze test, we excluded mice that did not conclude the 10 trials within 20 min of the test. In the OL and NOR tests, we excluded animals showing very low explorative behavior. This was defined as less than 20 s of direct object exploration during the 15-min test. Following these criteria, a total of 5 mice among the NOR, NOL, and T-maze tests were excluded.

### Immunofluorescence and cell counting

C57BL/6J mice or SST^+^ mice were deeply anesthetized at P5, P7, or P45, and transcardially perfused with PBS and then with 4% paraformaldehyde (PFA) in 100 mM phosphate buffer solution (pH 7.4). Their brain was collected, postfixed for 24 hours (12 hours for P5 and P7 staining) in the same fixative solution, cryoprotected in 30% sucrose in PBS, and then coronally cut in 40-μm-thick (50 μm for P5 and P7 staining) slices with a microtome (Microm HM 450 Sliding Microtome equipped with Freezing Unit Microm KS34, Thermo Scientific). For immunostaining, free-floating slices were first permeabilized in PBS containing 0.3% Triton X-100, incubated for 1 hour in blocking buffer (5% normal goat serum in PBS 0.1% Triton X-100) and then incubated overnight with the following antibodies (in blocking buffer): PV (1:800, Swant #PV-27), MBP (1:400, Cell Signaling #78896), OLIG2 (1:200, abcam #ab109186), PLP-1 (1:1000, abcam #ab9311), CASP3 (1:500, R&D Systems #AF835), and GABA (1:2000, abcam #ab17413). Fluorophore-conjugated (Alexa Fluor 488, Alexa Fluor 568, and Alexa Fluor 647) goat secondary antibodies (1:500; Thermo Fisher Scientific) were used for detection. Samples were counterstained with the nuclear dye Hoechst-33342 (Sigma Pharmaceuticals). Fluorescent images of brain slices were acquired with a Leica SP5 confocal scanning microscope equipped with a 20× (NA 0.7) or 40× (NA 1.25) oil-immersion objective.

For cell counts, stack of images spanning the whole thickness of the slice (2 μm; 20 z-stacks) were captured. Images of PV and SST neurons and double-staining GABA/CASP3 were acquired from the dorsal CA1 hippocampal region or PFC. OLIG2, PLP-1, and MBP-positive cells were acquired at the level of the fornix of the corpus callosum. All immunolabeled cells were counted in each optical section using the ImageJ “Cell Counter” plugin and normalized to the total section volume, except for PV interneurons in the hippocampus that were normalized on area. The fluorescence density was estimated in maximal projection of 20 z-stacks and was calculated with the ImageJ function “Integrated Density” with background subtraction. All histological quantifications were performed by an operator blind to the experimental groups. Data were obtained from two to three randomly chosen slices from each animal and averaged. At least 5 animals were used for each experimental group.

### Biochemistry

Hippocampal samples were homogenized in ice-cold homogenization buffer (320 mM sucrose, 1 mM EDTA, and 10 mM tris, pH 7.4) supplemented with 1 mM PMSF, 10 mM NaF, 2 mM sodium orthovanadate, and 1% (v/v) of a protease and phosphatase inhibitor cocktail (Sigma # P0044). Homogenates were first centrifuged at 800*g* to remove nuclei and debris. The resulting supernatant was further centrifuged at 9200*g*. The protein concentration of samples was determined with the BCA kit (Pierce). To perform immunoblot, protein extracts were diluted in lithium-dodecyl-sulfate sample buffer (Thermo Fisher Scientific), with the addition of 50 mM dithiothreitol (Sigma). To avoid NKCC1 and KCC2 protein precipitation or aggregation, all samples were warmed at 40°C for 5 min (*31*). Equivalent amounts of proteins (20 μg) were loaded on the 4–12% Bis-tris NuPAGE precast gels (Invitrogen), and electrophoresis were performed with MOPS buffer (Life Technologies). Next, gels were transferred overnight at 4°C onto nitrocellulose membranes (GE Healthcare) with tris-glycine transfer buffer (25 mM tris-base, 192 mM glycine, and 20% methanol). Equal amounts of protein loading was verified by staining with 0.1% Ponceau. Membranes were blocked for 1 hour in 5% milk in tris-buffered saline (10 mM tris and 150 mM NaCl, pH 8.0) plus 0.1% Tween 20 and incubated overnight at 4°C with primary antibodies: rabbit anti-actin (Sigma, catalog no: A2066; 1:10,000), mouse anti-NKCC1 (clone T4c, Developmental Studies Hybridoma Bank; 1:4000), rabbit anti-KCC2 (Millipore catalog n° 07-432; 1:4000), rabbit anti-MBP (Cell Signaling, catalog n° 78896; 1:1000), and rabbit anti-PLP-1 (Abcam, catalog n° ab9311; 1:1000). Next, membranes were washed and incubated for 2 hours at room temperature (RT) with HRP-conjugated goat secondary antibodies (Thermo Fisher Scientific; 1:10,000). Membranes were developed with SuperSignal West Pico chemiluminescent substrate (Thermo Fisher Scientific). The chemiluminescent signals were acquired on the LAS 4000 Mini imaging system (GE Healthcare). Bands were later quantified by measuring the mean intensity of the band signal using ImageQuant software (GE Healthcare). Uncropped blots can be found in fig. S13. For KCC2 analysis, we considered the monomeric form.

### Electron microscopy

Postnatal day 45 C57BL/6J mice were deeply anesthetized and transcardially perfused with a solution containing 2% PFA + 2% glutaraldehyde in 0.1 M cacodylate buffer (CB), pH 7.4. Brains were collected and then postfixed in 2% PFA + 2% glutaraldehyde in 0.1 M CB overnight at 4°C. Then, brains were washed in CB buffer and 300-μm-thick coronal slices were made using a vibratome (VT 1000S, Leica Biosystems). After several washes in 0.1 M CB buffer, the slices were postfixed in 1% osmium tetroxide in the same buffer as before for 2 hours at RT, and stained overnight at 4°C in an aqueous 1% uranyl acetate solution. After several washes in milli-Q water, the samples were dehydrated in a graded ethanol series (70%-90%-96%-100%) and embedded in EPON resin. Sections of about 70 nm, corresponding to portions of the corpus callosum, were cut with a diamond knife on a Leica EM UC6 ultramicrotome. TEM images were collected with a Jeol JEM 1011 (Jeol, Japan) electron microscope with a thermionic source and a maximum acceleration voltage of 100 kV, and recorded with a Gatan Orius SC1000 series CCD (charge-coupled device) camera. For each sample, five to seven micrographs were collected from the corpus callosum and used for myelin analysis. Measurements and image processing were performed using ImageJ software. The longest and shortest axon diameters and the widest and the thickest myelin widths were calculated for each myelinated axon. The degree of myelination was quantitatively evaluated by determining the *G* ratios, which were calculated by dividing the diameter of the axon by the diameter of the entire myelinated axon. Mean axon diameters were obtained from measurement of the axon circumference. Analysis was performed blinded to experimental groups and at least 100 axons were measured for each sample.

### Electroencephalogram

Twenty-eight- to 35-day-old (P28-P35) C57BL/6J (CTRL and JB1-treated) male mice underwent chronic surgery before the experiments. Mice were anesthetized with 2% isoflurane/0.8% oxygen and placed into a stereotaxic apparatus (Stoelting, Wood Dale, IL, USA). A heating pad kept the animal body temperature constant at 37°C for the whole duration of the anesthesia. Response to tail pinching, respiration rate, and vibrissae movement were monitored throughout the surgery to assess the level of anesthesia. Before the beginning of the surgery, mice were administered dexamethasone (4 mg/kg Dexadreson, MSD Animal Health, Milan, Italy) via intramuscular injection. The head was first shaved and disinfected and then the scalp was infiltrated with lidocaine and then removed to expose the skull. Mice were implanted with two stainless steel screws, one positioned over prefrontal cortex (0.8 lateral and 1.7 anterior from bregma) and one over the parietal cortex (1.5 lateral and 2.2 posterior from bregma) after drilling small holes at the indicated positions. A custom stainless-steel headplate was glued to the skull using epoxy glue. The headplate was secured to the skull with dental cement. At the end of the surgery, the animals were administered antibiotic (BAYTRIL, Bayer, Leverkusen, Germany) intraperitoneally. Animals recovered for 1 week after surgery before starting the subsequent experimental procedures.

After recovery, mice were first habituated to the experimenter and then trained to remain still inside a custom 3D-printed tube, while being head-restrained for a progressively increasing amount of time (≤1 hour). Recordings were performed after this habituation period. On the day of the experiment, the mouse was positioned in the tube and head-fixed and stainless steel electrodes were tightly secured onto the implanted screws. EEG signals were recorded for >11 min (average recording duration: 19.4 ± 0.8 min). Signals were amplified with an AM amplifier (AM-system, Carlsborg, WA, USA), filtered in the bandwidth 0.1 to 500 Hz, and digitized at 10 kHz with a Digidata 1440 (Axon Instruments, Union City, CA, USA).

Recorded EEG signals were then analyzed with custom MATLAB scripts. The full EEG trace was segmented in 1-min-long bins. The signal was low-pass–filtered at 100 Hz and down sampled to 1 kHz. For each bin, we calculated a synchronization index, i.e., the ratio between the signal power in the frequency band (0.1 to 4 Hz) and the frequency band [4 to 100 Hz (*80*–*82*)]. Only bins with a synchronization index <1.5 were concatenated and considered for further analysis. The power spectrum of the resulting signal was calculated and the signal powers for each frequency band of interest were computed. As previously described in young adult born preterm (*29*), a high frequency–to–low frequency ratio was calculated on the concatenated trace, by computing the ratio between the signal power in a high-frequency band (7.5 to 20.5 Hz) and the low-frequency band (0.5 to 7.5 Hz).

### Electrophysiology in vitro

Patch-clamp experiments were performed in P28 to 60 mice. Animals were briefly anesthetized with isoflurane and transcardially perfused with ice-cold cutting solution with the following composition: 220 mM sucrose, 7 mM MgCl_2_, 2.5 mM KCl, 1.25 mM NaH_2_PO_4_, 0.5 mM CaCl_2_, 25 mM NaHCO_3_, and 25 mM d-glucose (∼320 mOsm, pH 7.4, oxygenated with 95% O_2_ and 5% CO_2_). Then, brains were removed and placed in the same cutting solution in a vibratome chamber. Coronal slices (270 μm thick, VT1000S Leica Microsystem vibratome) were prepared from the dorsal hippocampus of control and JB1-treated mice, and then allowed to recover for 45 min at 35°C in a solution containing 117 mM NaCl, 2.5 mM KCl, 1.25 mM NaH_2_PO_4_, 3 mM MgCl_2_, 0.5 mM CaCl_2_, 25 mM NaHCO_3_, and 25 mM glucose (∼310 mOsm, pH 7.4, oxygenated with 95% O_2_ and 5% CO_2_). All recordings were performed at RT in artificial cerebrospinal fluid (ACSF) composed of 117 mM NaCl, 2.5 mM KCl, 1.25 mM NaH_2_PO_4_, 1 mM MgCl_2_, 2 mM CaCl_2_, 25 mM NaHCO_3_, and 25 mM glucose (∼300 mOsm, pH 7.4, oxygenated with 95% O_2_ and 5% CO_2_). Patch pipettes (resistance, 3 to 5 megohms) were made from thick-wall borosilicate glass capillaries (Sutter Instrument, catalog #B150–86-7.5). Access resistance was monitored online with Clampex 10.2 (Axon Instruments), and only cells with less than 20 megohms and with a maximum of 20% variations were included in the analysis.

For spontaneous postsynaptic current recordings, glutamatergic conductances were recorded by clamping cells at −65 mV (sEPSCs), whereas GABAergic conductances were recorded by clamping the same cells at 0 mV (sIPSCs). For these experiments, patch pipettes were filled with the following solution: 130 mM Cs-methanesulfonate, 5 mM CsCl, 5 mM NaCl, 2 mM MgCl_2_, 0.1 mM EGTA, 10 mM Hepes, 0.05 mM CaCl_2_, 2 mM Na_2_ATP, and 0.44 mM NaGTP. pH was adjusted to 7.3 with CsOH. Signals were sampled at 20 kHz, and low-pass–filtered at 2 kHz with an Axon Multiclamp 700B (Molecular Devices). Frequency and amplitude of synaptic events were analyzed using MiniAnalysis (Synaptosoft).

For current clamp recording, patch pipettes were filled with the following solution: 130 mM K-Gluconate, 7 mM KCl, 10 mM Hepes, 4 mM MgATP, 0.6 mM EGTA, 0.3 mM NaGTP, and 10 mM phospho-creatine. pH was adjusted to 7.3 with KOH. Rheobase recordings were performed by injecting small depolarizing currents (10-pA increments, 500 ms for each sweep). To generate the “input–output curve,” cells were injected with a depolarizing step (from 50 pA to 250 pA, 50-pA increments, 500 ms for each sweep). Signals were sampled at 50 kHz, and low-pass–filtered at 10 kHz with an Axon Multiclamp 700B (Molecular Devices). For the experiment related to Fig. 5, rheobase and input–output curve were compared between vehicle (DMSO) or GNX bath application (1 μM).

Passive and active properties were recorded with the same internal solution used to record the rheobase and the input–output curve. The resting membrane potential (RMP) was measured immediately after reaching the whole-cell configuration, while injecting no current (*I* = 0). The input resistance was calculated from the linear voltage deflection in response to a small current step (from −40 to +30 pA, 10-pA increments, 500 ms for each sweep). All active electrical properties were calculated from the first generated AP during the rheobase recording. The AP threshold was calculated through phase plane plot (Clampfit 10.7). The AP amplitude was calculated as the difference between the AP threshold and the AP peak. The fast after-hyperpolarization was calculated as the difference between the AP threshold and the most negative potential after the AP firing. The AP half-width parameter was measured at the half of the AP amplitude.

Tonic GABA_A_R-mediated currents were evaluated by voltage clamping the recorded neurons at 0 mV in whole-cell, patch-clamp configuration with the same internal solution used to record sEPSCs and sIPSCs. Tonic current values were determined as the shift of baseline holding currents before and after the application of the GABA_A_R antagonist bicuculline (20 μM). All-point histograms were generated from the recorded currents for both pre- and post-bicuculline epochs. The histograms were then fitted with a Gaussian distribution (Clampfit 10.7) to the negative side of the histograms to avoid IPSC contaminations. Then, the difference between the peaks of the pre- and post-bicuculline Gaussians distributions was considered the value of the tonic current, as previously described in published methods (*83*). Signals were sampled at 20 kHz, and low-pass–filtered at 2 kHz with an Axon Multiclamp 700B (Molecular Devices).

### Human studies

#### Participants

Participants were all very preterm (born below 32 weeks of gestation) surviving infants weighing less than 1500 g at birth admitted between January 2015 and December 2016 to the Neonatal Intensive Care Unit of Gaslini Children’s Hospital (Genova, Italy). Of 176 very preterm infants, 155 survived (mortality rate of 11.9%, with a 3:2 male-to-female ratio). Of them, 146 underwent, in addition to serial cranial ultrasounds, a brain MRI scan at term-corrected age. In particular, our routine standard protocol (which includes at least T1, T2, diffusion weighted imaging, and susceptibility weighted imaging sequences) involved brain magnetic resonance scan performed at term equivalent age conducted on a 3-T scanner (Ingenia 3 T; Philips Healthcare, Best, the Netherlands) using a 32-channel dedicated neonatal head array coil during spontaneous sleep. Ex-premature children carrying major brain lesions (15 preterm newborns, either intraventricular hemorrhage > grade 2, or cerebellar hemorrhage, or periventricular leukomalacia), mild brain lesions (32 preterm newborns, either germinal matrix-intraventricular hemorrhage, or punctate cerebellar hemorrhage, or punctate white matter lesions), combined types of brain lesions (10 preterm newborns), or diagnosed with either a genetic or a malformative disorder (4 preterm newborns) were excluded, to prevent confounding factors on the neuropsychological evaluated outcome. Thirty-four were lost at follow-up. Fifty-one of them (30 females and 21 males) presented a brain MRI at Term Equivalent Age (TEA) free from brain lesions and completed the full 5-year follow-up assessment. Prenatal, perinatal, and postnatal features of the selected cohort are listed in table S17 while general neurological assessment is reported in table S18.

#### Neurological and neuropsychological evaluation

The standard follow-up care of the Gaslini Children Hospital consists of serial neurological and neuropsychological evaluations at fixed developmental stages during the first 36 months of life and a preschool assessment at 5 years. The local ethical committee (Comitato Etico Regione Liguria, Genoa, Italy) approved this prospective observational study (number 39354), which was conducted in accordance with the Declaration of Helsinki. Each infant’s parent provided informed written consent. The 5-year evaluation allows the assessment of complex mental skills, including cognition, language, higher orders of neuropsychological skills (e.g., executive functions), and behavioral processes, which develop after 2 years of age (*46*). All ex–very preterm children who were born in the defined time window and who survived without any grade of brain lesions and completed the follow-up assessments scheduled at 5 years of age were enrolled. Ex–very premature children were evaluated by professionals with more than 10 years of experience. For the purpose of this study, an ad hoc neuropsychological battery has been ideated, consisting of developmental assessment with Wechsler Preschool and Primary Scale of Intelligence—Fourth Edition (WPPSI-IV), NEPSY-II (“A Developmental NEuroPSYchological Assessment”—Second Edition) subscales of the Memory and Learning domain, and parent-proxy questionnaires to investigate subjects’ behavioral and social attitudes (see below).

#### WPPSI-IV general indexes

The VSI, PSI, and WMI from the WPPSI-IV (*43*, *47*) were used to evaluate different cognitive abilities in PT children. VSI evaluates the ability in organizing visual information, in understanding relationships between parts and the whole, in paying attention to detail, and in integrating visual and motor individual functions. It is composed of the Block Design and Object Assembly subtests.

PSI measures the child’s ability to discriminate simple visual information and to perform quickly and correctly visual search tasks. This index consists of the following subtests: Bug Search, Cancellation, and Animal Coding. WMI assesses child’s attention, concentration, and handling interference (control and inhibition) skills. It was composed of the picture memory and zoo locations subtests.

The minimum attributable index core is 40, while the maximum is 160.

Standard norms for quotients of WPPSI-IV indices report mean values of 100 ± 15 (*N* = 1200) (*43*, *47*). For gender analysis, male and female PT data were compared to data present in the follow-up study of the standard sample evaluating gender differences. Index scores <70 were considered pathological following standard norms (≤2 percentile rank in the standard norm) (*43*).

#### Zoo locations

The zoo locations WMI subtest has been separately analyzed to gather information about the child’s spatial memory. In this test, child views animal cards placed in specific locations on a grid for 3 to 5 s, depending on the administered item. Then, the child must place each animal card in the previous “zoo” locations. Each animal is repeated in multiple items, but is not in the same grid location across items. The participants continue to complete items until they make an error on two consecutive test items. A response is considered an error if the animal from the target stimulus is not recalled in the correct location. The minimum attributable score in the subtests is 1, while the maximum score is 19.

Standard norms for WPPSI-IV subtest indices report mean values of 10 ± 3 (*43*, *47*). For gender analysis, male and female PT data were compared to data present in the follow-up study of the standard sample (*84*), evaluating gender differences. A score ≤6 was considered pathological following standard norms (≤10 percentile rank in the standard norm) (*43*).

#### Memory for design delayed

The Memory for Design Delayed subtest from NEPSY-II was used to evaluate long-term visuospatial memory. In this test, the child is shown a grid containing 4 to 10 card pictures per page for 10 s. Then, the examiner switches the page. After 15 to 25 min, the child must choose the pictures previously seen from a set of pictures and distractors and place them in a new grid in the same location as previously shown. In 5-year-old children, the experiment is repeated for five pages. The test evaluates the abilities to remember the correct pictures (content score) and the correct position (spatial score) of each card. A response is considered correct if the child chooses the target card (content score) and places it in the correct position (spatial score). The minimum attributable score in the NEPSY-II subtests is 1, while the maximum score is 19. Standard scores for weighted scores of NEPSY-II domains report mean values of 10 ± 3 (*n* = 86) (*44*). A score ≤5 percentile rank was considered pathological following standard norms (≤5 percentile rank in the standard norms) (*44*).

#### Autism quotient

The autism quotient for the children-parent questionnaire was used to evaluate autistic traits (*45*). This is a parent-proxy questionnaire consisting of 50 items in a four-point Likert scale. The minimum attributable AQ-C score is 0, indicating no autistic traits while the maximum score is 150, being equal to full endorsement on an autistic condition. The mean value of AQ-C among TDC in the study sample was 41.7 ± 18.6 for total children (*N* = 1225), 45.7 ± 20 for males (*N* = 607), and 37.7 ± 16.1 for females (*N* = 618). The authors showed that in a large sample of children with ASD (*N* = 539), a score of 76 showed both high sensitivity (95%) and high specificity (95%) in detecting children with autism and were therefore used as cutoff for pathological score identification (*45*). In the study sample, 4.3% of total TDC, 7.1% of male TDC, and 1.6% of female TDC scored above this cutoff.

### Statistical analysis

All the statistical analysis except for proteomic and phospho-proteomic experiments were performed with GraphPad Prism software (GraphPad Software, USA). Before running statistical analysis, the normality of the data was assessed with the Shapiro-Wilk normality test, with 95% (mice) or 99% (human) confidence. For comparison between CTRL and JB1 mice, we performed Student’s *t* test (normal data distribution) or Mann-Whitney (nonnormal data distribution) tests or Fisher Exact test or two-way analysis of variance (ANOVA) RM followed by all pairwise Sidak post hoc test. For comparison between four groups, we performed two-way ANOVA or two-way ANOVA RM, followed by all pairwise Tukey post hoc test. *P* values <0.05 were considered significant. Outliers were excluded only from the final pool of data by the ROUT test (*Q* = 1%). For proteomic and phospho-proteomic analysis, the Peptide Quant Pivot Report and Protein Quant Pivot Report generated by Spectronaut were statistically evaluated using Perseus software version 1.6.15.0 (5). Kinase enrichment was obtained with the webserver Kinase Enrichment Analysis 3 (KEA3) (6). GO enrichment analysis and Reactome pathway analysis were performed using the Gene Ontology Resources (gene.ontology.org), which use Fisher’s exact test with FDR < 0.05. The GO analysis was performed for biological processes (BP), cellular components (CC), and molecular functions (MF). Enrichment analysis for psychiatric disorder was performed using the SFARI (Simons Foundation Autism Research Initiative) database (gene.sfari.org) and the MATLAB hypergeometric probability function hygepdf. For comparison between human preterm data and standard norms means, one-sample *t* test was performed and results are presented as the means ± SEM. For comparison between frequencies of pathological children in our preterm dataset and standard norms, Fisher’s exact test was performed between the % of preterm children and the percentile rank reported in standard norm. For the AQ-C parent questionnaire, Fisher’s exact test was performed between the % of preterm children scoring above the autism cutoff and the % of TDC children scoring above the cutoff in the study sample. Significance was set at *P* < 0.01 for comparison with standard norm and *P* < 0.05 for comparison between datasets (male versus female).
