# Supplementary material for: Early IGF-1 receptor inhibition in mice mimics preterm human brain disorders and reveals a therapeutic target
Source: Sci Adv. 2024 Mar 1;10(9):eadk8123. doi: 10.1126/sciadv.adk8123 (PMC10906931; doi:10.1126/sciadv.adk8123)
Supplement: Supplementary file 1 — Figs. S1 to S13 Tables S1 to S18 [file sciadv.adk8123_sm.pdf]

Science Advances
AAAS

Supplementary Materials for

Early IGF-1 receptor inhibition in mice mimics preterm human brain disorders and reveals a therapeutic target

Alberto Potenzieri *et al.*

Corresponding author: Laura Cancedda, laura.cancedda@iit.it

Sci. Adv. **10**, eadk8123 (2024)
DOI: 10.1126/sciadv.adk8123

This PDF file includes:

Figs. S1 to S13
Tables S1 to S18

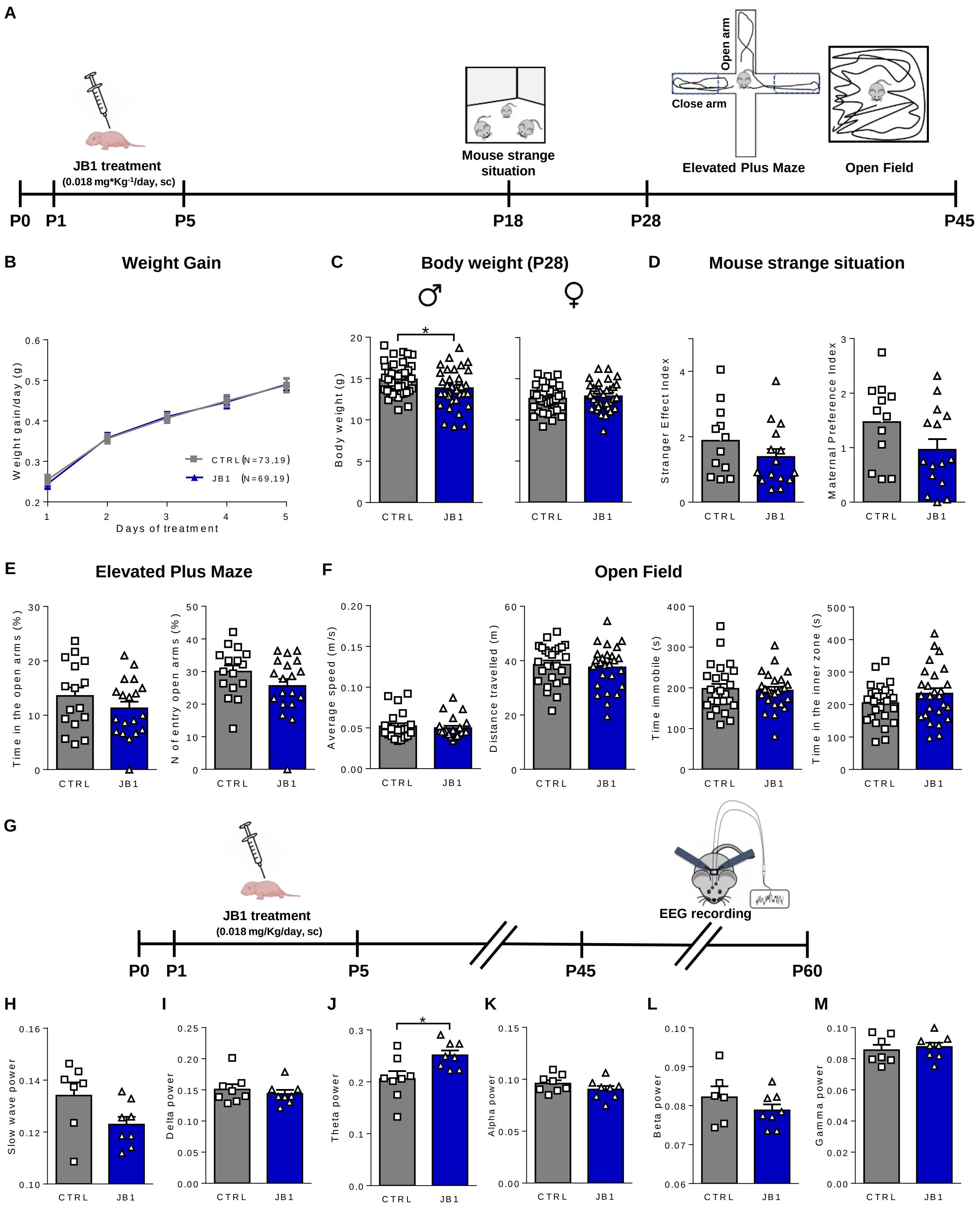

A
JB1 treatment
(0.018 mg*Kg-1/day, sc)
Mouse strange situation
Open arm
Close arm
Elevated Plus Maze
Open Field
P0
P1
P5
P18
P28
P45
B
Weight Gain
Weight gain/day (g)
Days of treatment
CTRL (N=73,19)
JB1 (N=69,19)
C
Body weight (P28)
Body weight (g)
CTRL
JB1
D
Mouse strange situation
Stranger Effect Index
Maternal Preference Index
E
Elevated Plus Maze
Time in the open arms (%)
N of entry open arms (%)
F
Open Field
Average speed (m/s)
Distance travelled (m)
Time immobile (s)
Time in the inner zone (s)
G
JB1 treatment
(0.018 mg/Kg/day, sc)
EEG recording
P60
H
Slow wave power
I
Delta power
J
Theta power
K
Alpha power
L
Beta power
M
Gamma power

Figure S1. Early postnatal IGF-1R inhibition leads to significant reduction of body weight in male adolescent mice, no significant effects in particular behavioural tasks, and increased theta power spectrum of EEG recordings. (**A**) Experimental protocol with pharmacological treatment and timing of specific behavioural testing. (**B**) Body-weight gain curve during the treatment of C57BL/6J pups (P1-P5) with JB1 or vehicle (CTRL) in our study. Numbers in parenthesis indicate the number of animals, litters for each experimental group. (**C**) Mean ± SEM and single animal cases of body weight from P28 male (left) and female (right) JB1-treated and CTRL mice in our study. Left, two-tailed Student's t-test, $t = 2.243$, $^*p < 0.05$. (**D**) Quantification of the mean ± SEM and single animal cases of stranger effect index (left) and maternal preference index (right) during mouse strange situation test for the same animals as in Figure 2E. (**E**) Quantification of the mean ± SEM and single animal cases of the percentage of time spent (left) and percentage of number of entry (right) in the open arms of the Elevated Plus Maze test in JB1-treated and CTRL mice. (**F**) Quantification of the mean ± SEM and single animal cases of the average speed (left), distance travelled (middle left), time immobile (middle right) and time in the inner zone (right) in the Open field test in JB1-treated and CTRL mice. (**G**) Experimental protocol with pharmacological treatment and timing of EEG recordings in awake head-fixed male mice. (**H**) Slow wave (0.5-1 Hz) power normalized to total power EEG power. Bars represent the normalized power for all recorded animals ± SEM, and symbols represent data points for each recorded mouse. $N = 8$ for JB1 and $N = 7$ for CTRL. (**I**). Delta wave (2-4 Hz) power normalized to total power EEG power Bars represent the normalized power for all recorded animals ± SEM, and symbols represent data points for each recorded mouse. $N = 8$ for JB1 and $N = 8$ for CTRL. (**J**)Theta wave (4-8 Hz) power normalized to total power EEG power. Bars represent the normalized power for all recorded animals ± SEM, and symbols represent data points for each recorded mouse. $N = 8$ for JB1 and $N = 8$ for CTRL. Two-tailed Student's t-test, $t = 2.638$, $^*p < 0.05$. (**K-M**) Alpha waves (8-14 Hz, $N = 8$ for JB1 and $N = 8$ for CTRL, k), beta waves (14-30 Hz, $N = 8$ for JB1 and $N = 6$ for CTRL, l), gamma waves (30-90 Hz, $N = 8$ for JB1 and $N = 7$ for CTRL, m) power normalized to total EEG power. Bars represent the normalized power for all recorded animals ± SEM, and symbols represent data points for each recorded mouse. Schematic cartoons by BioRender.com.

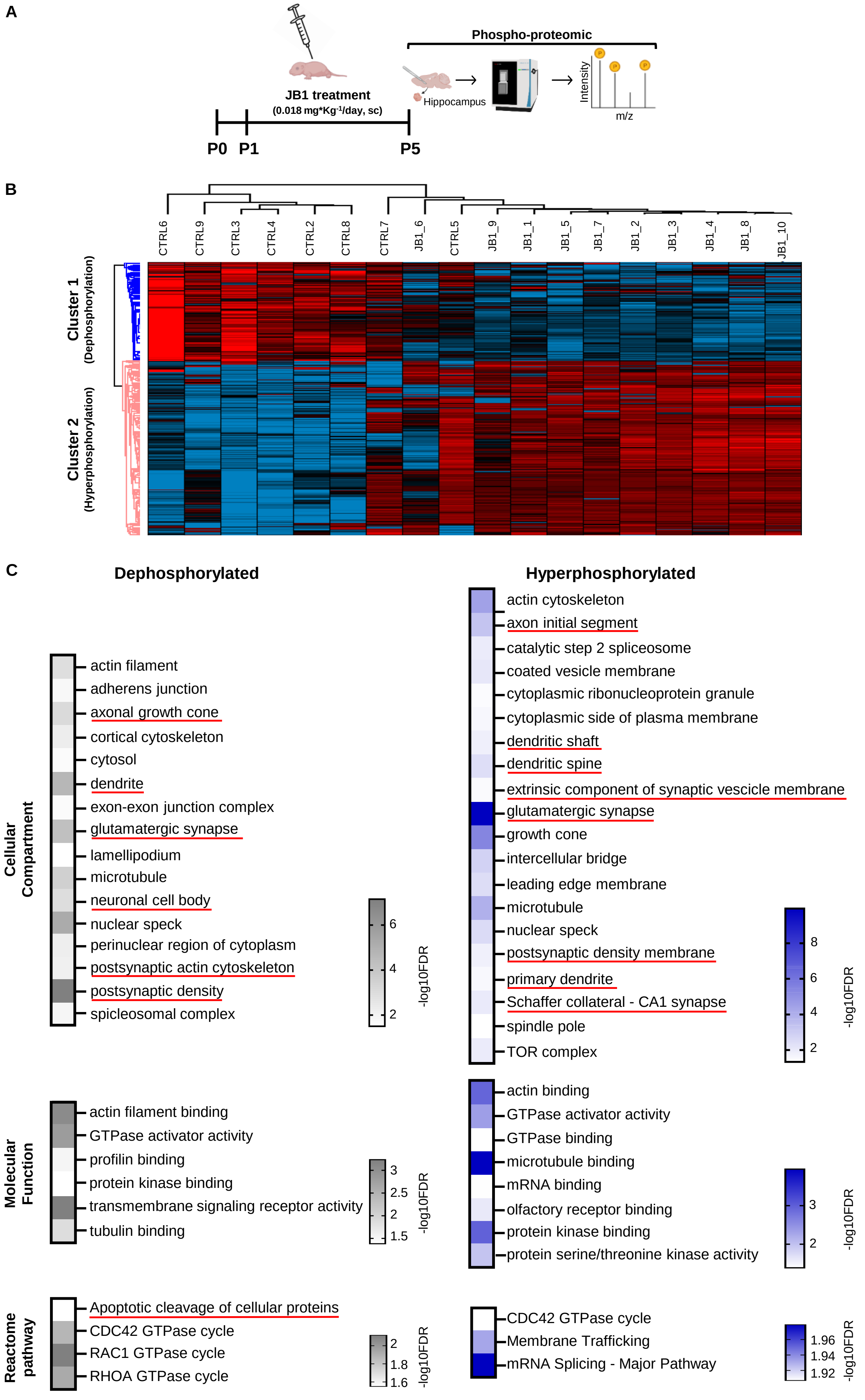

A
Phospho-proteomic
JB1 treatment
(0.018 mg*Kg-1/day, sc)
Hippocampus
Intensity
m/z
P0
P1
P5
B
CTRL6
CTRL9
CTRL3
CTRL4
CTRL2
CTRL8
CTRL7
JB1_6
CTRL5
JB1_9
JB1_1
JB1_5
JB1_7
JB1_2
JB1_3
JB1_4
JB1_8
JB1_10
Cluster 1 (Dephosphorylation)
Cluster 2 (Hyperphosphorylation)
C
Dephosphorylated
Hyperphosphorylated
Cellular Compartment
actin filament
adherens junction
axonal growth cone
cortical cytoskeleton
cytosol
dendrite
exon-exon junction complex
glutamatergic synapse
lamellipodium
microtubule
neuronal cell body
nuclear speck
perinuclear region of cytoplasm
postsynaptic actin cytoskeleton
postsynaptic density
spicleosomal complex
6
4
2
-log10FDR
actin cytoskeleton
axon initial segment
catalytic step 2 spliceosome
coated vesicle membrane
cytoplasmic ribonucleoprotein granule
cytoplasmic side of plasma membrane
dendritic shaft
dendritic spine
extrinsic component of synaptic vescicle membrane
glutamatergic synapse
growth cone
intercellular bridge
leading edge membrane
microtubule
nuclear speck
postsynaptic density membrane
primary dendrite
Schaffer collateral - CA1 synapse
spindle pole
TOR complex
8
6
4
2
-log10FDR
Molecular Function
actin filament binding
GTPase activator activity
profilin binding
protein kinase binding
transmembrane signaling receptor activity
tubulin binding
3
2.5
2
1.5
-log10FDR
actin binding
GTPase activator activity
GTPase binding
microtubule binding
mRNA binding
olfactory receptor binding
protein kinase binding
protein serine/threonine kinase activity
3
2
-log10FDR
Reactome pathway
Apoptotic cleavage of cellular proteins
CDC42 GTPase cycle
RAC1 GTPase cycle
RHOA GTPase cycle
2
1.8
1.6
-log10FDR
CDC42 GTPase cycle
Membrane Trafficking
mRNA Splicing - Major Pathway
1.96
1.94
1.92
-log10FDR

Figure S2. Systemic IGF-1R inhibition in WT mouse pups leads to acute phospho-proteomic changes associated with neuron and synapse development (**A**) Experimental protocol with pharmacological treatment and timing of the phospho-proteomic experiment. (**B**) Heatmap showing clustering of significant (FDR <0.05) dephosphorylated (cluster 1) or hyperphosphorylated (cluster 2) phosphosites in JB1-treated pups sacrificed at the end of the treatment. Values are normalized on z-score. (**C**) Gene Ontology (GO, cellular compartment, and molecular function) and Reactome pathway analysis for the significantly differentially dephosphorylated (left) or hyperphosphorylated (right) proteins (from the same analysis in Figure 1B). The color bar on the right indicates -log10FDR for the statistically significant (FDR <0.05) top 20 (hierarchy for fold enrichment) enriched cellular compartments, molecular functions or Reactome pathways. Terms related to neurons or synapses are underlined in red. Schematic cartoons by BioRender.com.

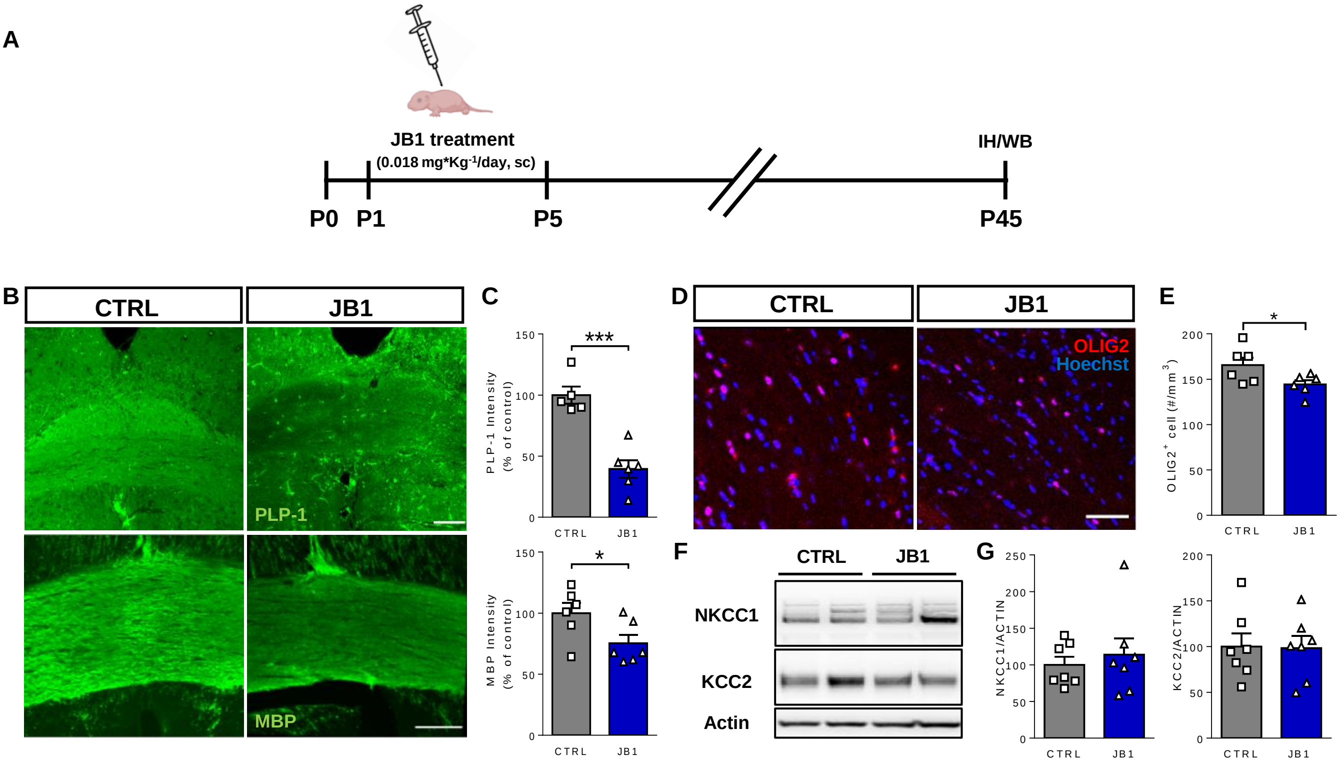

Figure S3. Early postnatal IGF-1R inhibition leads to myelination deficits and no alterations in the expression of Cl transporters. (A) Experimental protocol with pharmacological treatment and timing of histological/biochemical experiments. (**B**) Representative images of PLP-1 (top, green) and MBP (bottom, green) fluorescent staining from brain slices at the level of the corpus callosum from P45 littermates treated early in life with JB1 or vehicle (CTRL). Scale bars: 100 µm. (**C**) Quantification of PLP-1 (top) and MBP (bottom) protein fluorescence in experiments as in B. Bars represent the average intensity for all analysed animals ± SEM and symbols represent data points for each animal (2-3 slices per animal, 4 litters). Top, two-tailed Student's t-test, t = 5.941, *** $p < 0.001$. Bottom, two-tailed Student's t-test, t = 2.236, *$p < 0.05$. (**D**) Representative images of OLIG2 (red)/Hoechst (blue) fluorescent staining from brain slices at the level of the corpus callosum from P45 littermates treated early in life with JB1 or vehicle (CTRL). Scale bar: 100 µm. (**E**) Quantification of the density of OLIG2^{+} cells in experiments as in D. Bars represent the average density of OLIG2^{+} cells of all the analysed animals ± SEM, and symbols represent single data points for each animal (2-3 slices per animal, from 4 independent litters). Two-tailed Student's t-test, t = 2.270, *$p < 0.05$. (**F**) Representative immunoblots for NKCC1 and KCC2, proteins from hippocampal lysates obtained from P45 brains of littermates previously treated with JB1 or vehicle (CTRL) as pups. (**G**) Quantification of NKCC1 (left) and KCC2 (right) protein levels normalized to actin in experiments as in F. Bars represent the average percentage of NKCC1 or KCC2 over CTRL of all analysed animals ± SEM, and symbols represent data points for each animal. Results derived from 3 independent experiments. Schematic cartoons by BioRender.com.

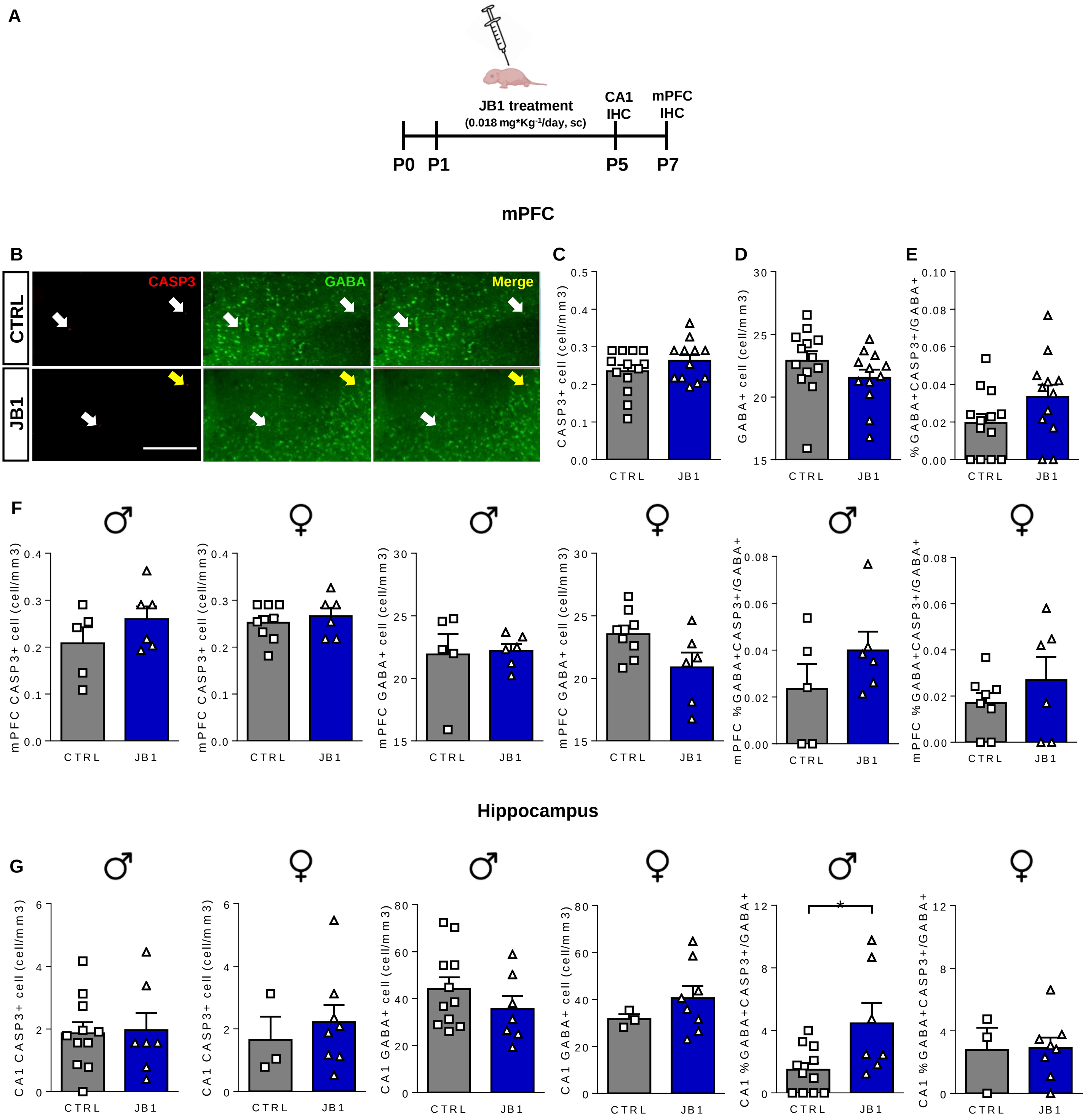
A
JB1 treatment
(0.018 mg*Kg-1/day, sc)
CA1 IHC
mPFC IHC
P0
P1
P5
P7
mPFC
B
CASP3
GABA
Merge
CTRL
JB1
C
D
E
CASP3+ cell (cell/mm3)
GABA+ cell (cell/mm3)
%GABA+CASP3+/GABA+
F
mPFC CASP3+ cell (cell/mm3)
mPFC GABA+ cell (cell/mm3)
mPFC %GABA+CASP3+/GABA+
Hippocampus
G
CA1 CASP3+ cell (cell/mm3)
CA1 GABA+ cell (cell/mm3)
CA1 %GABA+CASP3+/GABA+
CTRL
JB1
*

Figure S4. Systemic IGF-1R inhibition leads to significantly increased apoptosis of GABAergic interneurons in the hippocampus (but not in the mPFC) of WT male (but not female) mouse pups. (**A**) Experimental protocol with pharmacological treatment and timing of immunohistochemistry (IHC) experiments. (**B**) Representative images of caspase-3 (CASP3, red) and GABA (green) fluorescent staining from mPFC slices of P7 male pup littermates treated with JB1 or vehicle (CTRL). Scale bar: 60 μm; White arrows: CASP3-positive cells. Yellow arrow: CASP3 and GABA double-positive cell. (**C**) Quantification of the density of CASP3-positive cells in brain slices in the experiments as in B. Bars represent the average density of CASP3-positive cells of all the analysed animals ± SEM, and symbols represent single data points for each animal (2-3 slices per animal from 3 independent litters). (**D**) Quantification of the density of GABA-positive cells in brain slices in the experiments as in B. Bars represent the average density of GABA-positive cells of all the analysed animals ± SEM, and symbols represent single data points for each animal (2-3 slices per animal from 3 independent litters). (**E**) Quantification of the density of CASP3- and GABA-double positive cells normalized on the total GABA-positive cells in brain slices in the experiments as in B. Bars represent the average density of CASP3- and GABA- double positive cells expressed as a percentage of total GABA-positive cells of all the analysed animals ± SEM, and symbols represent single data points for each animal (2-3 slices per animal from 3 independent litters). (**F**) Quantification of the density of CASP3-positive, GABA-positive, or CASP3- and GABA-double positive cells normalized on the total GABA-positive cells in experiments as in B, with data segregated by animal's gender. Bars represent the average density of CASP3-positive (left), GABA-positve (middle), or CASP3- and GABA-double positive cells normalized on the total GABA-positive cells of all the analysed animals ± SEM, and symbols represent single data points for each animal. (**G**) Quantification of of the density of CASP3-positive, GABA-positive, or CASP3- and GABA-double positive cells normalized on the total GABA-positve cells in the same experiment as in Fig. 4L, with data segregated by animal's gender. Bars represent the average density of CASP3-positive (left), GABA-positive (middle), or CASP3- and GABA-double positive cells normalized on the total GABA-positive cells of all the analysed animals ± SEM, and symbols represent single data points for each animal. Two-tailed Student's t-test, $t = 2.541$, *$p < 0.05$. Schematic cartoons by BioRender.com.

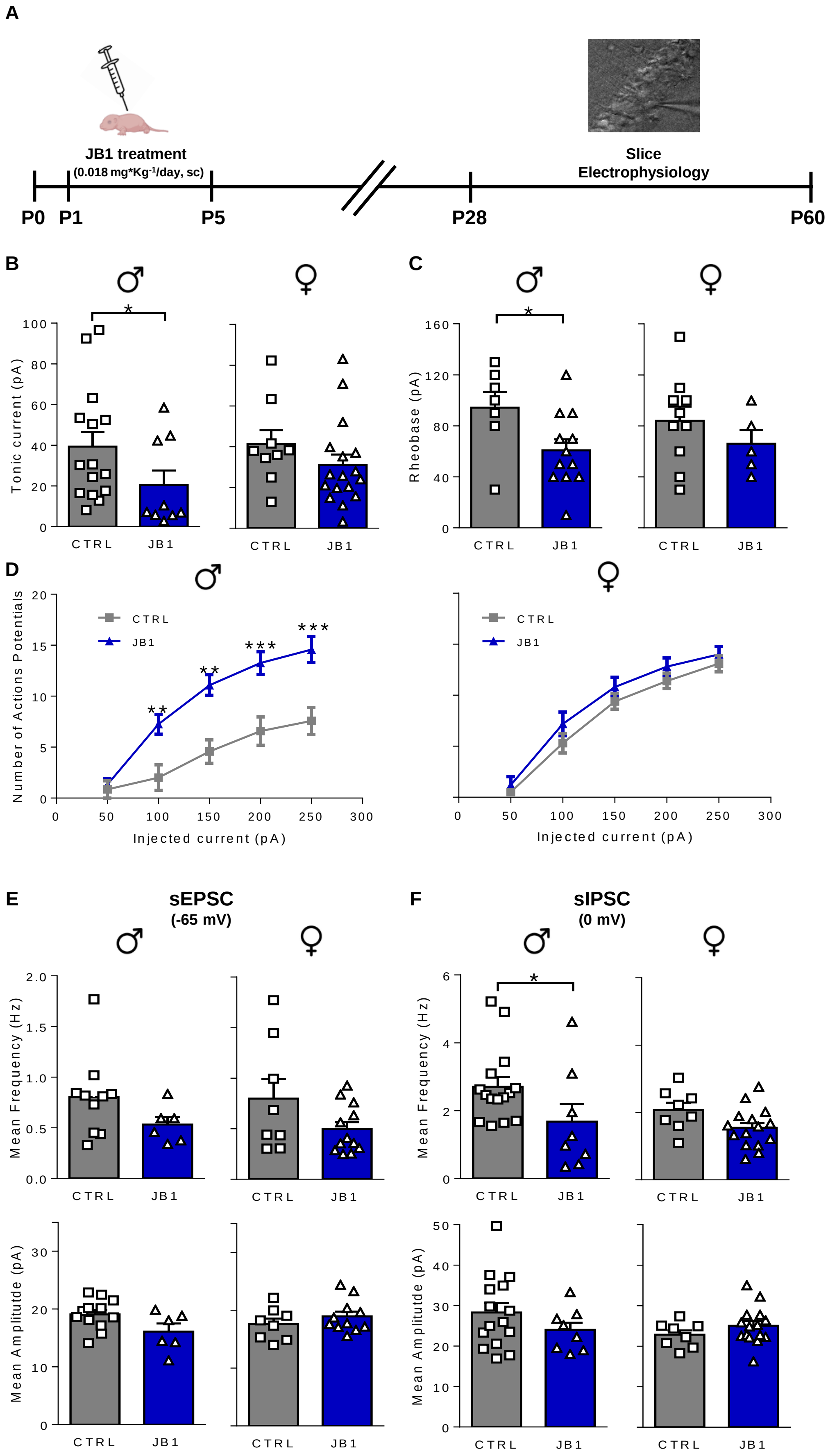

A
JB1 treatment
(0.018 mg*Kg-1/day, sc)
Slice
Electrophysiology
P0
P1
P5
P28
P60
B
Tonic current (pA)
CTRL
JB1
C
Rheobase (pA)
CTRL
JB1
D
Number of Actions Potentials
CTRL
JB1
Injected current (pA)
E
sEPSC
(-65 mV)
Mean Frequency (Hz)
Mean Amplitude (pA)
F
sIPSC
(0 mV)
Mean Frequency (Hz)
Mean Amplitude (pA)

Figure S5. Early postnatal IGF-1R inhibition leads to significantly reduced GABAergic inhibition and increased intrinsic excitability only in male adolescent mice. (**A**) Experimental protocol with pharmacological treatment and timing of electrophysiological recordings on acute hippocampal brain slices. (**B**) Quantification of the amplitude of tonic bicuculline-induced currents in the same experiments as in Fig. 4F, with data segregated by animal's gender. Bars represent the average ± SEM and symbol represent data points for each recorded cell (6 CTRL male mice *vs* 4 JB1 male mice; 5 CTRL female mice *vs* 7 JB1 female mice). Left, Mann-Whitney test, $U = 31.00$, $*p < 0.05$. (**C**) Quantification of rheobase. Bars represent the average ± SEM and symbols represent data point for each recorded cell as in Fig. 4D, with data segregated by animal's gender (3 CTRL male mice *vs* 6 JB1-treated male mice; 5 CTRL female mice *vs* 3 JB1-treated female mice). Left, two-tailed Student's t-test, $t = 2.288$, $*p < 0.05$. (**D**) Input–output curves depicting the average number of action potentials (± SEM) elicited by graded depolarizing currents in the same experiments as in Fig. 4C, with data segregated by animal's gender. Left, two-way ANOVA, $F_{treatment\ (1,\ 17)} = 14.29$, $*\ p < 0.01$, Sidak multiple comparison *post hoc* test, $**p < 0.01$, $***p < 0.001$. (**E**) Quantification of the sEPSCs frequency (top) and amplitude (bottom) in the same experiments as in Fig. 4H, with data segregated by animal's gender. Bars represent the average data for all analysed cells ± SEM, and symbols represent data points for each recorded cell (5 CTRL male mice *vs* 3 JB1-treated male mice; 4 CTRL female mice *vs* 5 JB1-treated female mice). (**F**) Quantification of the sIPSCs frequency (top) and amplitude (bottom) in same experiments as Fig.4J with data segregated by animal's gender. Bars represent the average data for all analysed cells ± SEM, and symbols represent data points for each recorded cell (7 CTRL male mice *vs* 4 JB1-treated male mice; 5 CTRL female mice *vs* 7 JB1-treated female mice). Mann-Whitney test, $U = 29.00$, $*p < 0.05$. Schematic cartoons by BioRender.com.

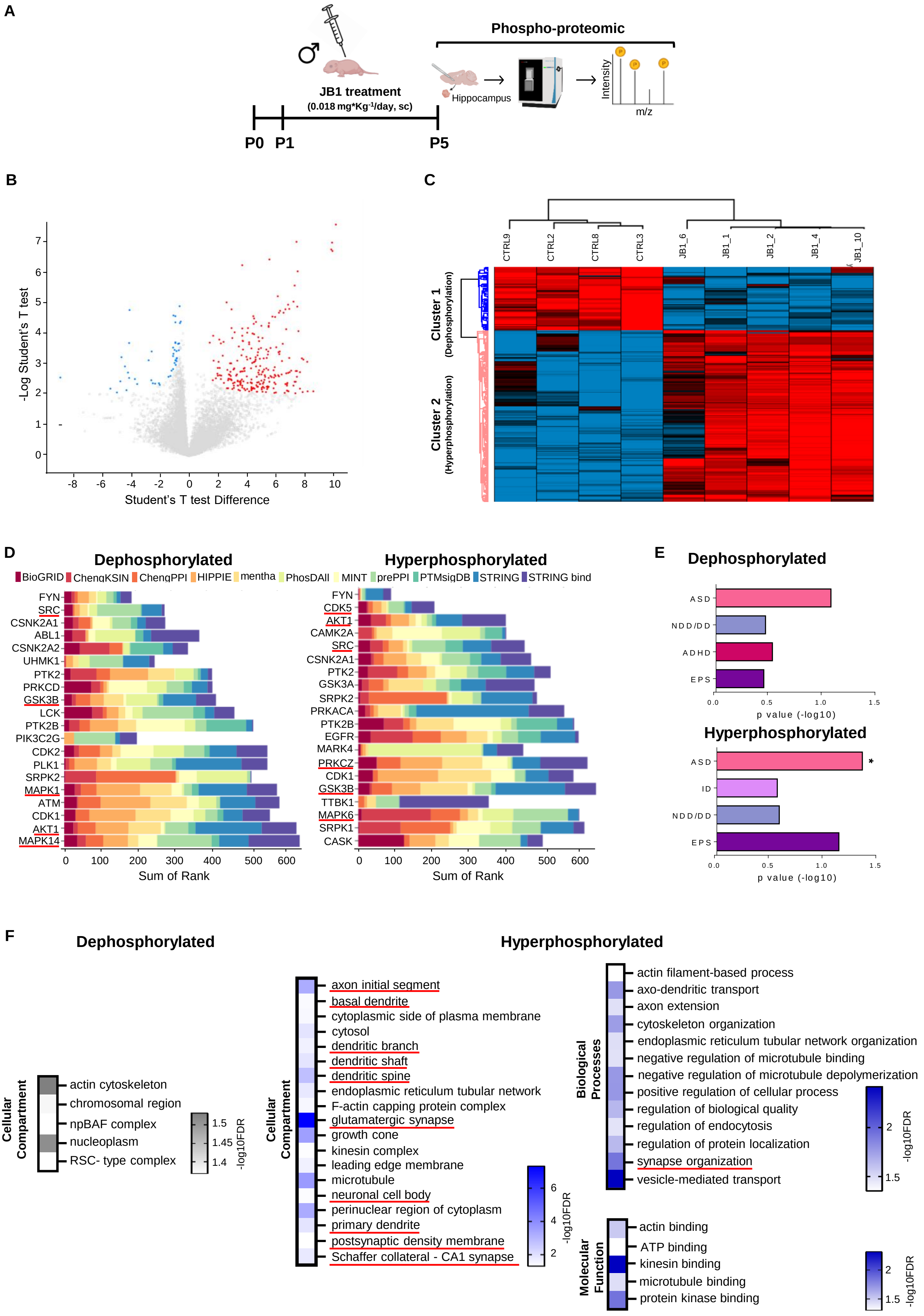
A
JB1 treatment
(0.018 mg*Kg-1/day, sc)
P0 P1 P5
Phospho-proteomic
Hippocampus
Intensity
m/z
B
-Log Student's T test
Student's T test Difference
C
CTRL9 CTRL2 CTRL8 CTRL3 JB1_6 JB1_1 JB1_2 JB1_4 JB1_10
Cluster 1 (Dephosphorylation)
Cluster 2 (Hyperphosphorylation)
D
Dephosphorylated
Hyperphosphorylated
BioGRID ChengKSIN ChengPPI HIPPIE mentha PhosDAll MINT prePPI PTMsigDB STRING STRING bind
FYN SRC CSNK2A1 ABL1 CSNK2A2 UHMK1 PTK2 PRKCD GSK3B LCK PTK2B PIK3C2G CDK2 PLK1 SRPK2 MAPK1 ATM CDK1 AKT1 MAPK14
FYN CDK5 AKT1 CAMK2A SRC CSNK2A1 PTK2 GSK3A SRPK2 PRKACA PTK2B EGFR MARK4 PRKCZ CDK1 GSK3B TTBK1 MAPK6 SRPK1 CASK
Sum of Rank
E
Dephosphorylated
ASD NDD/DD ADHD EPS
p value (-log10)
Hyperphosphorylated
ASD ID NDD/DD EPS
p value (-log10)
F
Dephosphorylated
Cellular Compartment
actin cytoskeleton
chromosomal region
npBAF complex
nucleoplasm
RSC- type complex
-log10FDR
Hyperphosphorylated
Cellular Compartment
axon initial segment
basal dendrite
cytoplasmic side of plasma membrane
cytosol
dendritic branch
dendritic shaft
dendritic spine
endoplasmic reticulum tubular network
F-actin capping protein complex
glutamatergic synapse
growth cone
kinesin complex
leading edge membrane
microtubule
neuronal cell body
perinuclear region of cytoplasm
primary dendrite
postsynaptic density membrane
Schaffer collateral - CA1 synapse
Biological Processes
actin filament-based process
axo-dendritic transport
axon extension
cytoskeleton organization
endoplasmic reticulum tubular network organization
negative regulation of microtubule binding
negative regulation of microtubule depolymerization
positive regulation of cellular process
regulation of biological quality
regulation of endocytosis
regulation of protein localization
synapse organization
vesicle-mediated transport
Molecular Function
actin binding
ATP binding
kinesin binding
microtubule binding
protein kinase binding

Figure S6. Systemic IGF-1R inhibition in male mouse pups leads to acute phospho-proteomic changes associated with ASD. (**A**) Experimental protocol with pharmacological treatment and timing of the phospho-proteomic experiment utilizing mass spectrometry. (**B**) Vulcano plot showing the phosphorylated/dephosphorylated phosphorylation sites differentially expressed between JB1-treated (N = 5 animals) and control (vehicle-treated; N = 4 animals) pup littermates sacrificed 1 hour after the last treatment at P5. Blue and red dots represent significantly dephosphorylated and hyperphosphorylated phosphorylation sites, respectively. s0 = 0.1 and FDR = 0.05. (**C**) Heatmap showing clustering of significant (FDR <0.05) dephosphorylated (cluster 1) or hyperphosphorylated (cluster 2) phosphosites in JB1-treated pups sacrificed at the end of the treatment. Values are normalized on z-score. (**D**) Kinase Enrichment Analysis of significantly dephosphorylated (left) and hyperphosphorylated (right) phospho-proteins, corresponding to differentially expressed phosphorylation sites showed in B. Bars represent the Mean Rank of the top 20 phospho-proteins based on multiple library databases (color-coded above). The kinases related to IGF-1 signalling are underlined in red. (**E**) Enrichment for neuropsychiatric disorder risk-genes (identified with SFARI gene archive) in the dephosphorylated (top) and hyperphosphorylated (bottom protein datasets shown in B). Hypergeometric test, *p < 0.05. ADHD: Attention Deficit and Hyperactivity Disorder; ASD: Autism Spectrum Disorder; EPS: Epilepsy; ID: Intellectual Disability; NDD/DD: NeuroDevelopmental Disorder/Developmental Disorder. (**F**) Gene Ontology (GO) analysis for the differentially dephosphorylated (left) or hyperphosphorylated (right) significantly expressed proteins corresponding to differentially expressed phosphorylation sites showed in B. The color bar on the right indicates -log10FDR for the statistically significant (FDR <0.05) enriched cellular compartments, biological processes, and molecular functions. Terms related to neurons or synapses are underlined in red. Schematic cartoons by BioRender.com.

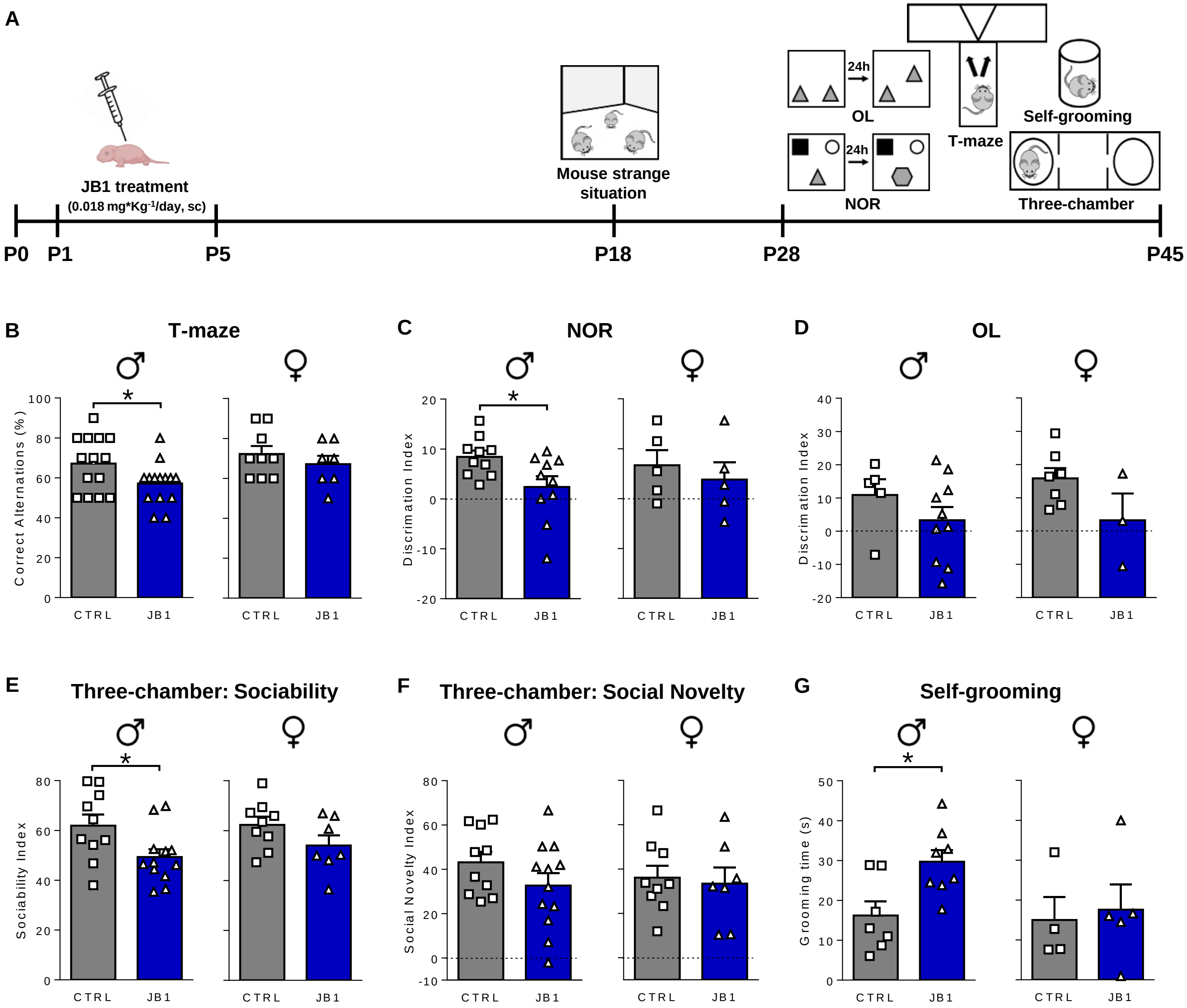

Figure S7. Early postnatal IGF-1R inhibition leads to significant cognitive and social deficits, and repetitive behaviours only in male adolescent mice. (**A**) Experimental protocol with pharmacological treatment and timing of specific behavioural testing. (**B**) Quantification of the mean ± SEM and single animal cases of the correct alternations in the T-maze test in the same experiments as in Fig. 2F, with data segregated by animal's gender. Two-tailed Student's t-test, $t = 2.141$, *$p < 0.05$. (**C**) Quantification of the mean ± SEM and single animal cases of the discrimination index in the NOR test in the same experiments as in Fig. 2G, with data segregated by animal's gender. Two-tailed Student's t-test, $t = 2.446$, *$p < 0.05$. (**D**) Quantification of the mean ± SEM and single animal cases of the discrimination index in the OL test in the same experiments as in Fig. 2H, with data segregated by animal's gender. (**E**) Quantification of the mean ± SEM and single animal cases of the sociability index in the three-chamber test in the same experiments as in Fig. 2I left, with data segregated by animal's gender. Left, two-tailed Student's t-test, $t = 2.389$, *$p < 0.05$. (**F**) Quantification of the mean ± SEM and single animal cases of the social novelty index in the three-chamber test in the same experiments as in Fig. 2I right, with data segregated by animal's gender. (**G**) Quantification of the mean ± SEM and single animal cases of the grooming time in the self-grooming test in the same experiments as in Fig. 2J with data segregated by animal's gender. Left, two-tailed Student's t-test, $t = 2.944$, *$p < 0.05$. Schematic cartoons by BioRender.com.

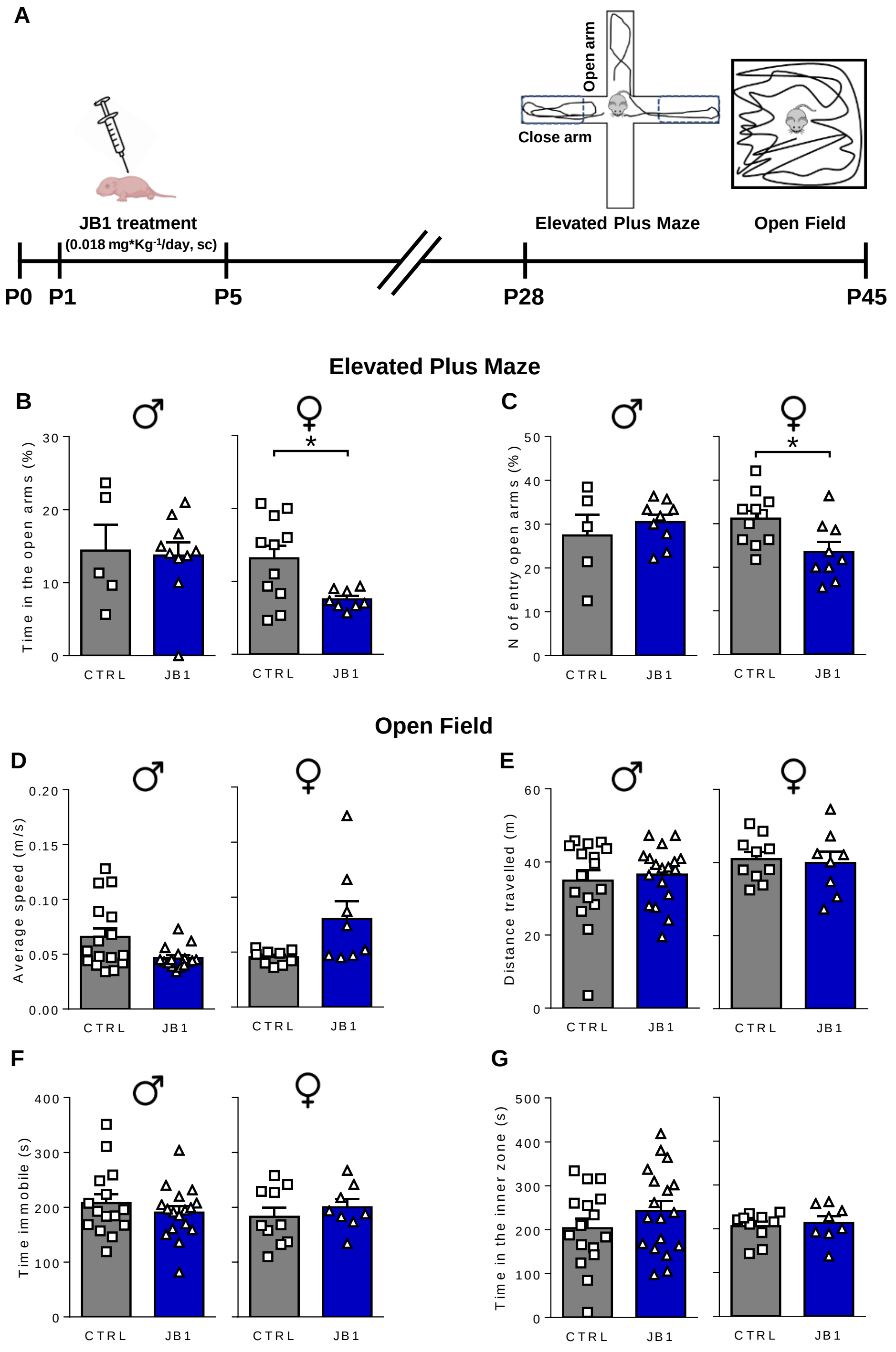
A
JB1 treatment
(0.018 mg*Kg-1/day, sc)
Open arm
Close arm
Elevated Plus Maze
Open Field
P0
P1
P5
P28
P45
Elevated Plus Maze
B
Time in the open arms (%)
C
N of entry open arms (%)
Open Field
D
Average speed (m/s)
E
Distance travelled (m)
F
Time immobile (s)
G
Time in the inner zone (s)
CTRL
JB1
♂
♀
*

Figure S8. Early postnatal IGF-1R inhibition leads to significantly increased anxiety only in female adolescent mice. (**A**) Experimental protocol with pharmacological treatment and timing of specific behavioural testing for anxiety. (**B**) Quantification of the mean ± SEM and single animal cases of the percentage of time spent in the open arms of the Elevated Plus Maze test in the same experiments as in Fig. S1E left, with data segregated by animal's gender. Right, two-tailed Student's t-test, $t = 2.694$, $^*p < 0.05$. (**C**) Quantification of the mean ± SEM and single animal cases of the percentage of number of entries in the open arms of the Elevated Plus Maze test in the same experiments as in Fig. S1E right, with data segregated by animal's gender. Right, two-tailed Student's t-test, $t = 2.677$, $^*p < 0.05$. (**D**) Quantification of the mean ± SEM and single animal cases of the average speed in the Open field test in the same experiments as in Fig. S1F left, with data segregated by animal's gender. (**E**) Quantification of the mean ± SEM and single animal cases of the distance travelled in the Open field test in the same experiments as in Fig. S1F middle left, with data segregated by animal's gender. (**F**) Quantification of the mean ± SEM and single animal cases of the time immobile in the Open field test in the same experiments as in Fig. S1F middle right, with data segregated by animal's gender. (**G**) Quantification of the mean ± SEM and single animal cases of the time in the inner zone in the Open field test in the same experiments as in Fig. S1F right, with data segregated by animal's gender. Schematic cartoons by BioRender.com.

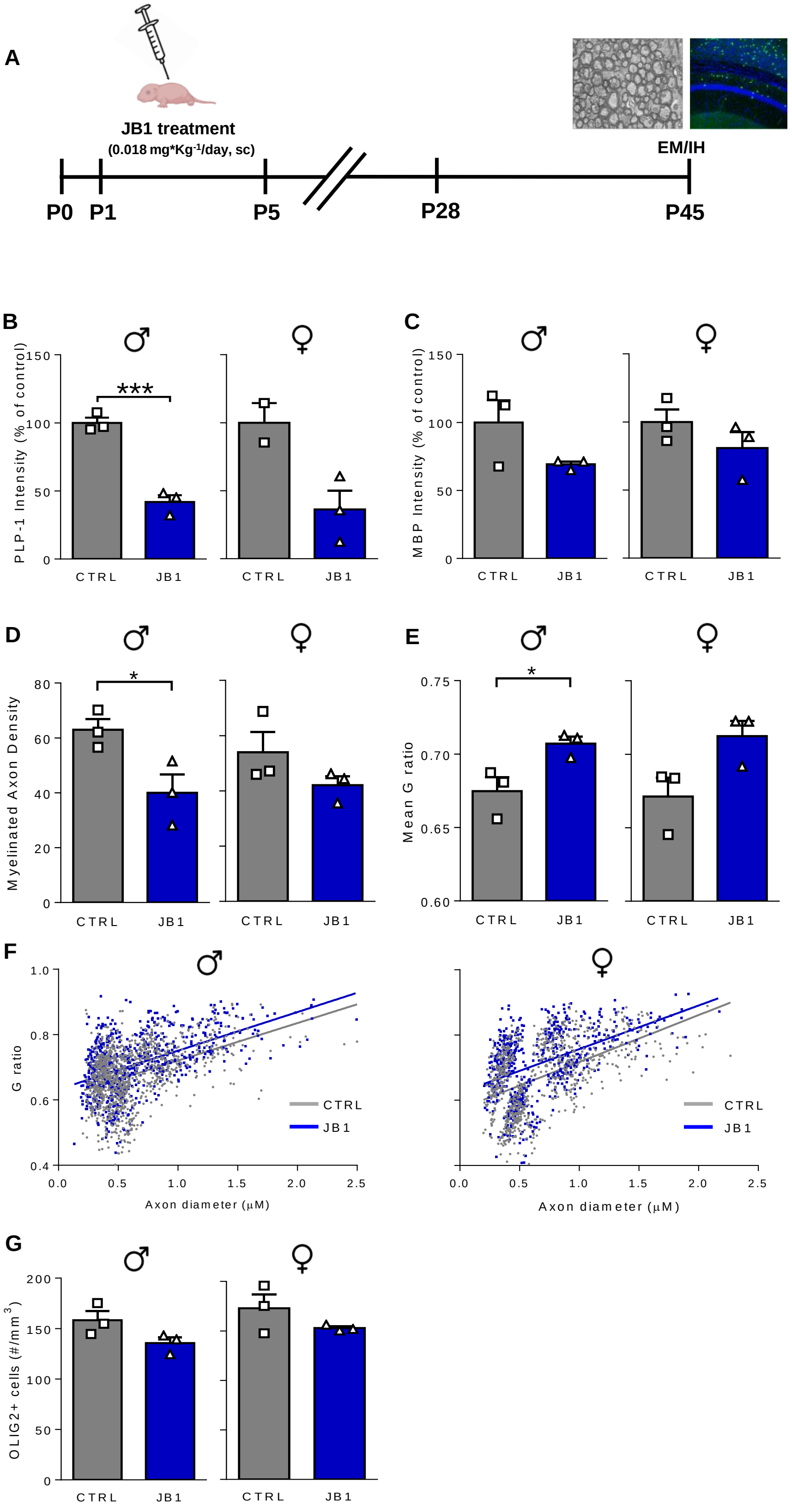
A
JB1 treatment
(0.018 mg*Kg-1/day, sc)
EM/IH
P0
P1
P5
P28
P45
B
PLP-1 Intensity (% of control)
***
CTRL
JB1
C
MBP Intensity (% of control)
D
Myelinated Axon Density
*
E
Mean G ratio
*
F
G ratio
CTRL
JB1
Axon diameter (μM)
G
OLIG2+ cells (#/mm3)

Figure S9. Early postnatal IGF-1R inhibition leads to myelination deficits in male adolescent mice. (**A**) Experimental protocol with pharmacological treatment and timing of histological/biochemical experiments. (**B**) Quantification of PLP-1 protein fluorescence in the same experiments of Fig. S3C top, with data segregated by animal's gender. Left, Bars represent the average intensity for all analysed animals ± SEM and symbols represent data points for each animal. Two-tailed Student's t-test, $t = 9.165$, ***$p < 0.001$. (**C**) Quantification of MBP protein fluorescence in the same experiments of Fig. S3C bottom, with data segregated by animal's gender. Bars represent the average intensity for all analysed animals ± SEM and symbols represent data points for each animal. (**D**) Quantification of myelinated axon density from the same experiments as in Fig. 3C, with data segregated by animal's gender. Bars represent the average ± SEM and symbols represent single data points for each animal. Left, two-tailed Student's t-test, $t = 2.940$, *$p < 0.05$. (**E**) Quantification of the mean G ratio from the same experiments as in Fig. 3E, with data segregated by animal's gender. Left, two-tailed Student's t-test, $t = 3.023$, *$p < 0.05$. (**F**) Scatter plot of the G ratio calculated for each individual axon *vs* its axon diameter from the same experiments as in Fig. 3D, with data segregated by animal's gender. Fitted lines are linear regressions. R square male: CTRL= 0.1641, JB1= 0.2592; R square female: CTRL= 0.2648, JB1= 0.2253 (**G**) Quantification of the mean density of $OLIG2^+$ fluorescent cells from the same experiments as in Fig S3E, with data segregated by animal's gender. Bars represent the average density of $OLIG2^+$ cells ± SEM, and symbols represent single data points for each animal. Schematic cartoons by BioRender.com.

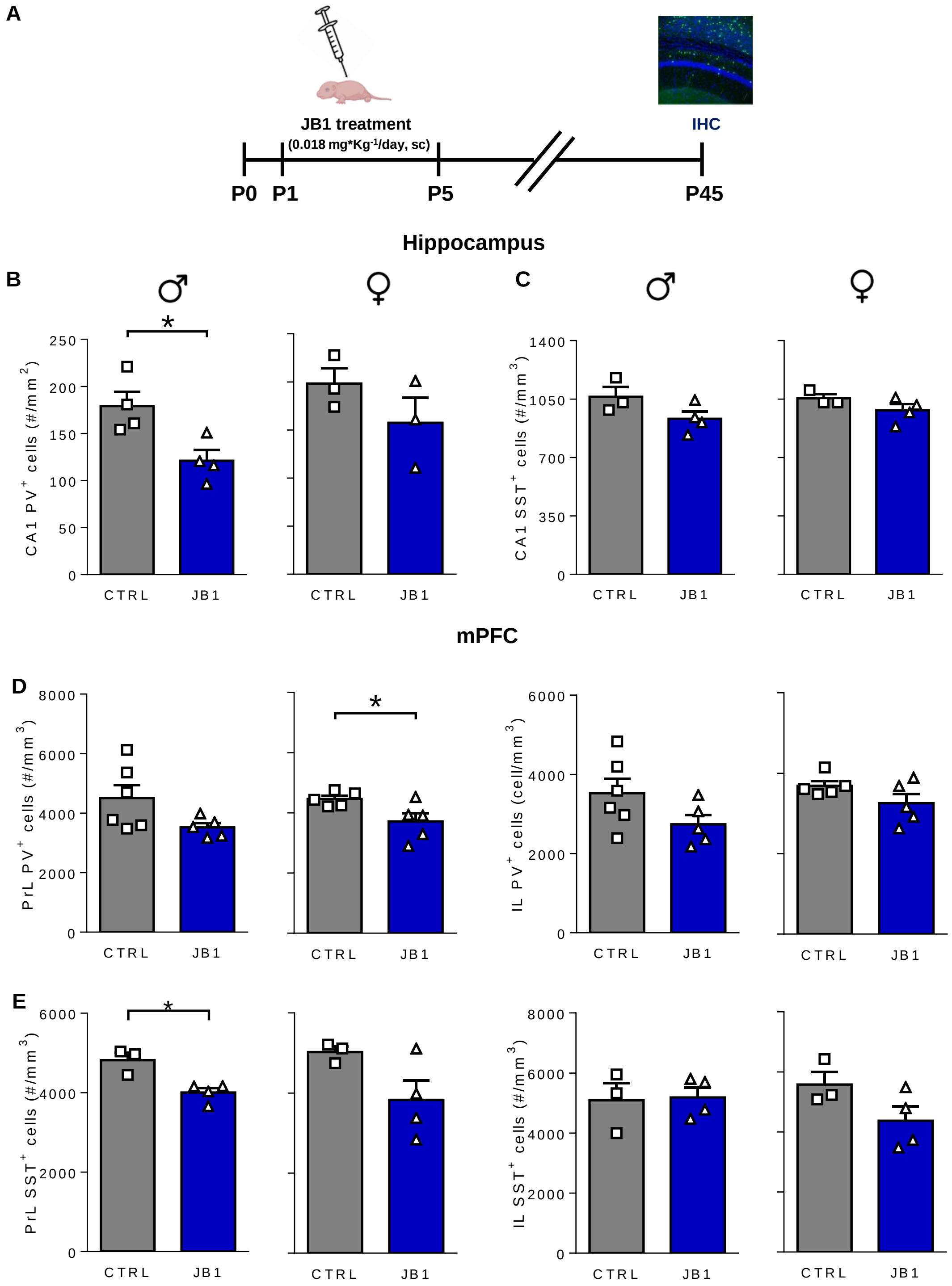

Figure S10. Early postnatal IGF-1R inhibition leads to a significant regional- and sex-biased loss of interneurons in adolescent mice. (A) Experimental protocol with pharmacological treatment and timing of histological experiments. **(B)** Quantification of the density of PV interneurons in the same experiments as in Fig. 3G, with data segregated by animal's gender. Bars represent the average density of PV-positive cells of all the analysed animals' ± SEM, and symbols represent single data points for each animal. Left, two-tailed Student's t-test, t = 3.099, *p < 0.05. (**C**) Quantification of the density of SST interneurons in the same experiments as in Fig. 3K, with data segregated by animal's gender. Bars represent the average density of SST-positive cells of all the analysed animals ± SEM, and symbols represent single data points for each animal. **(D)** Quantification of the density of PV interneurons in the same experiments as in Fig. 3I, with data segregated by animal's gender. Middle left, two-tailed Student's t-test, t = 2.494, *p < 0.05. Bars represent the average density of PV-positive cells of all the analysed animals ± SEM, and symbols represent single data points for each animal. *p<0.05, Student's t test. **(E)** Quantification of the density of SST interneurons in the same experiments as in Fig. 3M, with data segregated by animal's gender. Bars represent the average density of SST-positive cells of all the analysed animals ± SEM, and symbols represent single data points for each animal. Left, two-tailed Student's t-test, t = 3.906, *p<0.05. Schematic cartoons by BioRender.com.

A

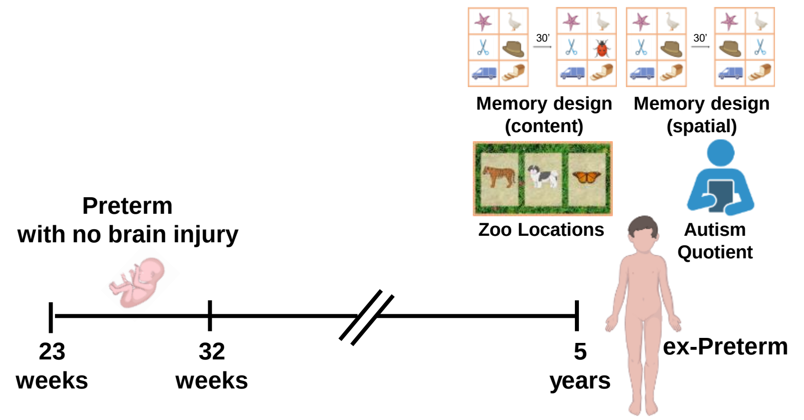

B

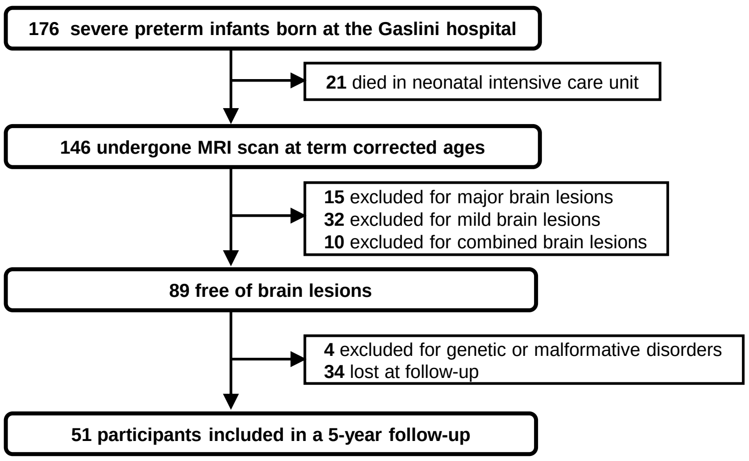

C

Brain function	Mice	Humans
Short-term memory	T-maze	Zoo Locations
Recognition memory	NOR	Memory Design Delayed (Content score)
Spatial memory	OL	Memory Design Delayed (Spatial score)
Sociabiility/ Repetitive	TC+SG	Autism Quotient (AQ-C)

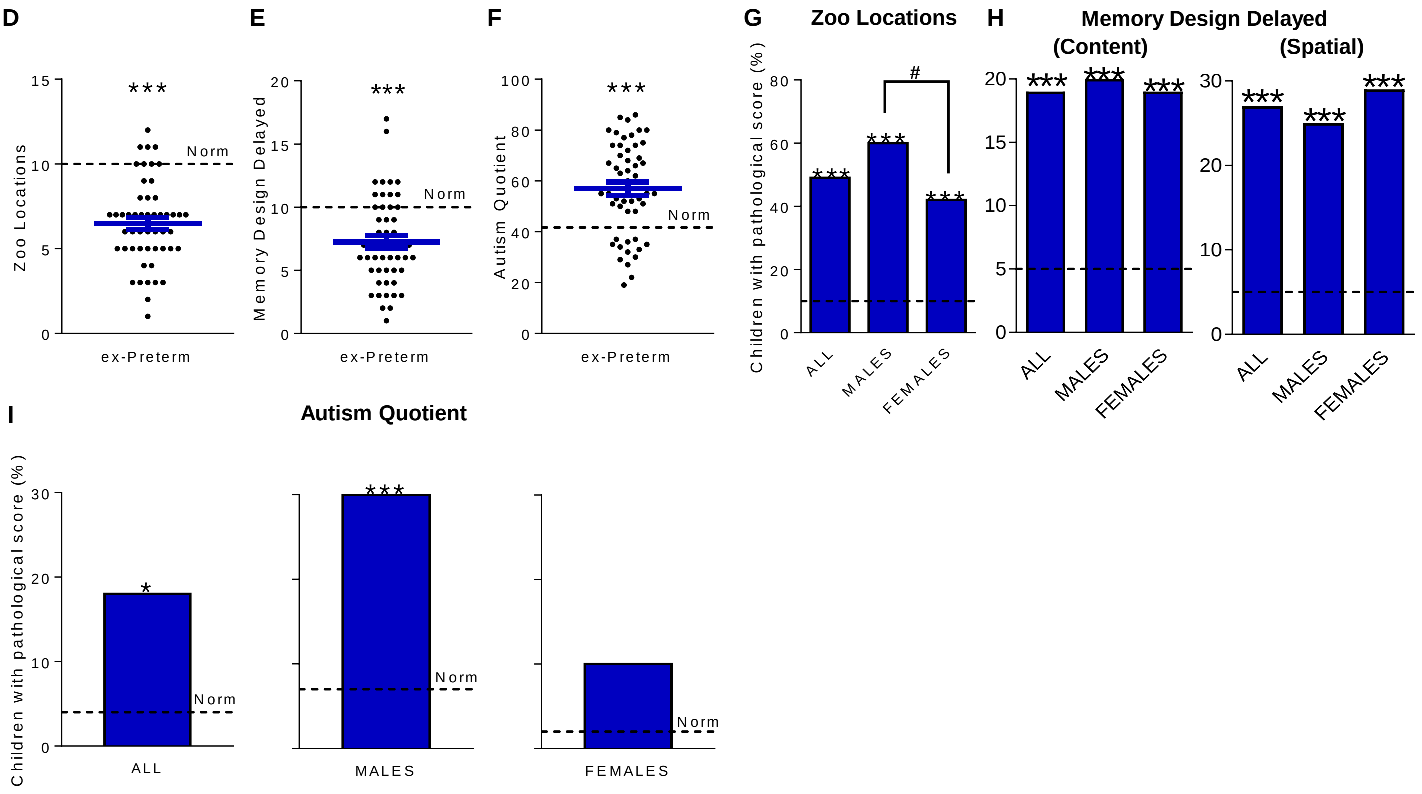

Figure S11. Ex-preterm children without diagnosed neonatal brain injury display cognitive deficits and autistic traits with a sex-dependent bias. (**A**) Timeline for the behavioural evaluation of ex-preterm children. (**B**) Clinical flowchart used to classify/exclude patients in the clinical study. (**C**) Table showing the parallelism between the behavioural tests performed in mice and in humans. (**D**) Quantification of the mean ± SEM (overlying line) and single ex-preterm child scoring (symbols) of the zoo locations test. The dashed line represents the standard norm of typically developing children from the literature. One-sample t-test, $t = 10.09$, ***$p < 0.0001$. (**E**) Quantification of the mean ± SEM (overlying line) and single ex-preterm child scoring (symbols) of the memory design delayed test. The dashed line represents the standard norm of typically developing children from the literature. One-sample t-test, $t = 5.596$, ***$p < 0.0001$. (**F**) Quantification of the mean ± SEM (overlying line) and single ex-preterm child scoring (symbols) of the Autism Quotient parent questionnaire. The dashed line represents the standard norm of typically developing children from the literature. One-sample t-test, $t = 5.898$, ***$p < 0.0001$. (**G**) Quantification of the percentage number of ex-preterm children with pathological scoring in the zoo locations test presented in D (males: 20 children; females: 31 children). The dashed line represents the percentile ranks of the standard norm of typically developing children from the literature with a cut off for scoring ≤ 6. Fisher's exact test, ***$p < 0.0001$. Male vs. female comparison, Fisher's exact test, #$p < 0.05$. (**H**) Quantification of the percentage number of ex-preterm children with pathological scoring in the memory design delayed (content score, left; spatial score, right) test presented in E (males: 20 children; females: 31 children). The dashed line represents percentile ranks of the standard norm of typically developing children from the literature with a cut off for scoring ≤ 5th percentile rank. Fisher's exact test, ***$p < 0.0001$. (**I**) Quantification of the percentage number of ex-preterm children scoring above the autism cut off in the Autism Quotient parent questionnaire presented in F (males: 20 children; females: 31 children). The dashed line represents the percentage of children in the standard norm of typically developing children from the literature with a cut off score ≥76. Fisher's exact test, *$p<0.01$, *** $p<0.0001$. Schematic cartoons by BioRender.com.

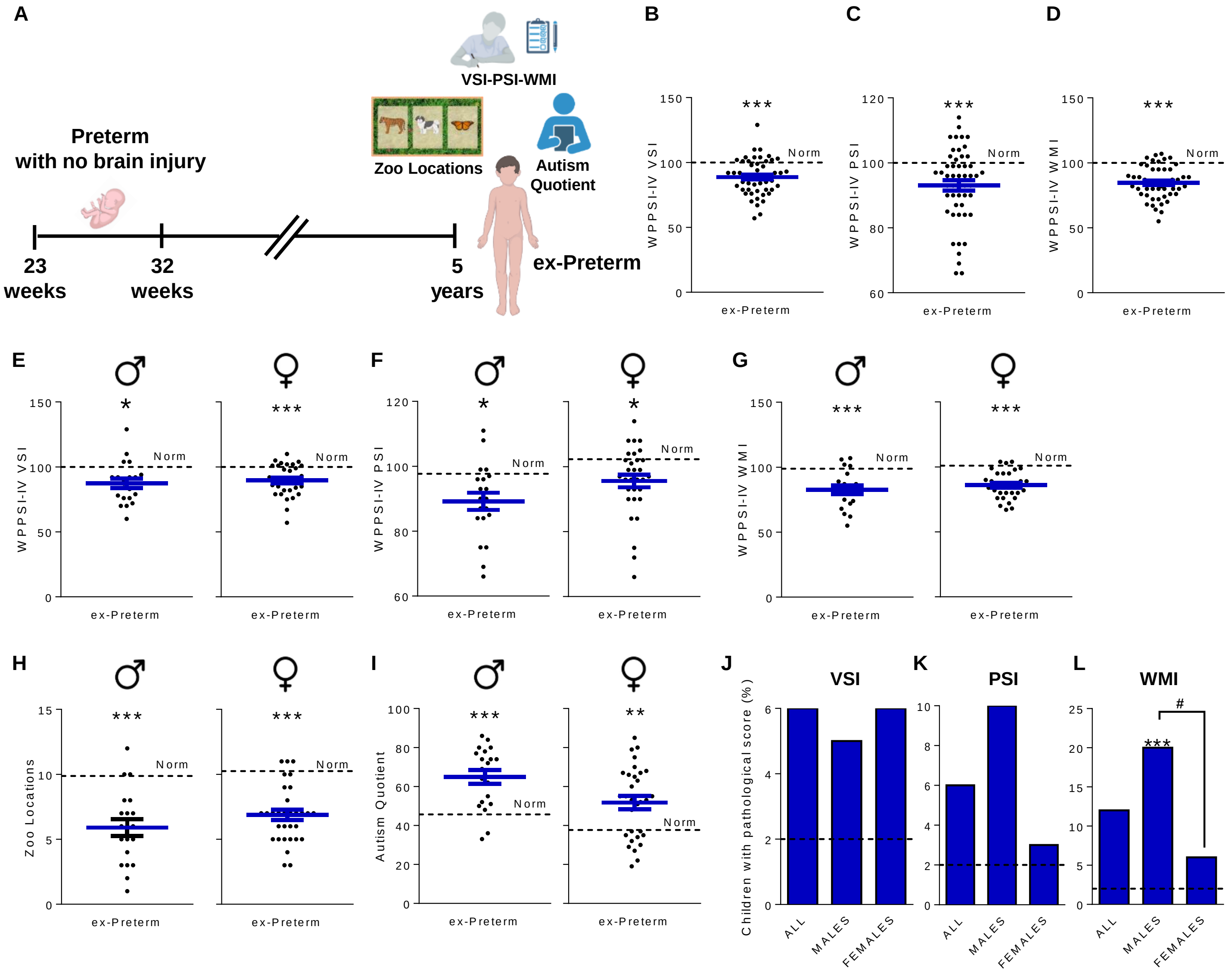
A
VSI-PSI-WMI
Zoo Locations
Autism
Quotient
Preterm
with no brain injury
23
weeks
32
weeks
5
years
ex-Preterm
B
C
D
E
F
G
H
I
J
K
L
WPPSI-IV VSI
WPPSI-IV PSI
WPPSI-IV WMI
Zoo Locations
Autism Quotient
Norm
ex-Preterm
***
**
*
Children with pathological score (%)
VSI
PSI
WMI
ALL
MALES
FEMALES
#

Figure S12. Preterm children without diagnosed neonatal brain injury perform below standard norms in cognitive tests and show increased autistic traits. (A) Timeline for behavioural evaluation of ex-preterm children of the same cohort presented in Fig.S11. (**B**) Quantification of the mean ± SEM (overlying line) and single ex-preterm child scoring (symbols) of the VSI test. The dashed line represents standard norm mean of typically developing children from the literature. One-sample t-test, $t = 5.805$, ***$p < 0.0001$. (**C**) Quantification of the mean ± SEM (overlying line) and single ex-preterm child scoring (symbols) of the PSI test. The dashed line represents standard norm mean of typically developing children from the literature. One-sample t-test, $t = 4.305$, ***$p < 0.0001$. (**D**) Quantification of the mean ± SEM (overlying line) and single ex-preterm child scoring (symbols) of the WMI test. The dashed line represents standard norm mean of typically developing children from the literature. One-sample t-test, $t = 8.883$, ***$p < 0.0001$. (**E**) Quantification of the mean ± SEM (overlying line) and single ex-preterm child scoring (symbols) of the VSI test presented in B, with data segregated for gender. The dashed line represents standard norm mean of typically developing children from the literature. Left, one-sample t-test, $t = 3.570$, *$p < 0.01$. Right, one-sample t-test, $t = 4.994$, ***$p < 0.0001$. (**F**) Quantification of the mean ± SEM (overlying line) and single ex-preterm child scoring (symbols) of the PSI test presented in C, with data segregated for gender. The dashed line represents standard norm mean of typically developing children from the literature. Left, one-sample t-test, $t = 3.220$, *$p < 0.01$. Right, one-sample t-test, $t = 3.478$, *$p < 0.01$. (**G**) Quantification of the mean ± SEM (overlying line) and single ex-preterm child scoring (symbols) of the WMI test presented in D, with data segregated for gender. The dashed line represents standard norm mean of typically developing children from the literature. Left, one-sample t-test, $t = 4.946$, ***$p < 0.0001$. Right, one-sample t-test, $t = 8.114$, ***$p < 0.0001$. (**H**) Quantification of the mean ± SEM (overlying line) and single ex-preterm child scoring (symbols) of the Zoo Locations test presented in Fig. S11D, with data segregated for gender. The dashed line represents standard norm mean. Left, one-sample t-test, $t = 6.240$, ***$p < 0.0001$. Right, one-sample t-test, $t = 8.603$, ***$p < 0.0001$. (**I**) Quantification of the mean ± SEM (overlying line) and single child ex-preterm scoring (symbols) of the Autism Quotient parent questionnaire presented in Fig. S11F, with data segregated for sex. The dashed line represents standard norm mean of typically developing children from the literature. Left, one-sample t-test, $t = 5.458$, ***$p < 0.0001$. Right, one-sample t-test, $t = 4.254$, **$p < 0.001$. (**J**) Quantification of the percentage number of ex-PT children with pathological scoring in the VSI test presented in B. The dashed line represents Percentile Ranks of standard norm of typically developing children from the literature with a cut off for scoring < 70. (**K**) Quantification of the percentage number of ex-preterm children with pathological scoring in the PSI test presented in C. The dashed line represent Percentile Ranks of standard norm of typically developing children from the literature with a cut off for scoring < 70. (**L**) Quantification of the percentage number of ex-preterm children with pathological scoring in the WMI test presented in D. The dashed line represents Percentile Ranks of standard norm of typically developing children from the literature with a cut off for scoring < 70. Fisher's exact test, ***$p < 0.01$; male *vs* female comparison, Fisher's exact test, # $p < 0.05$. Schematic cartoons by BioRender.com.

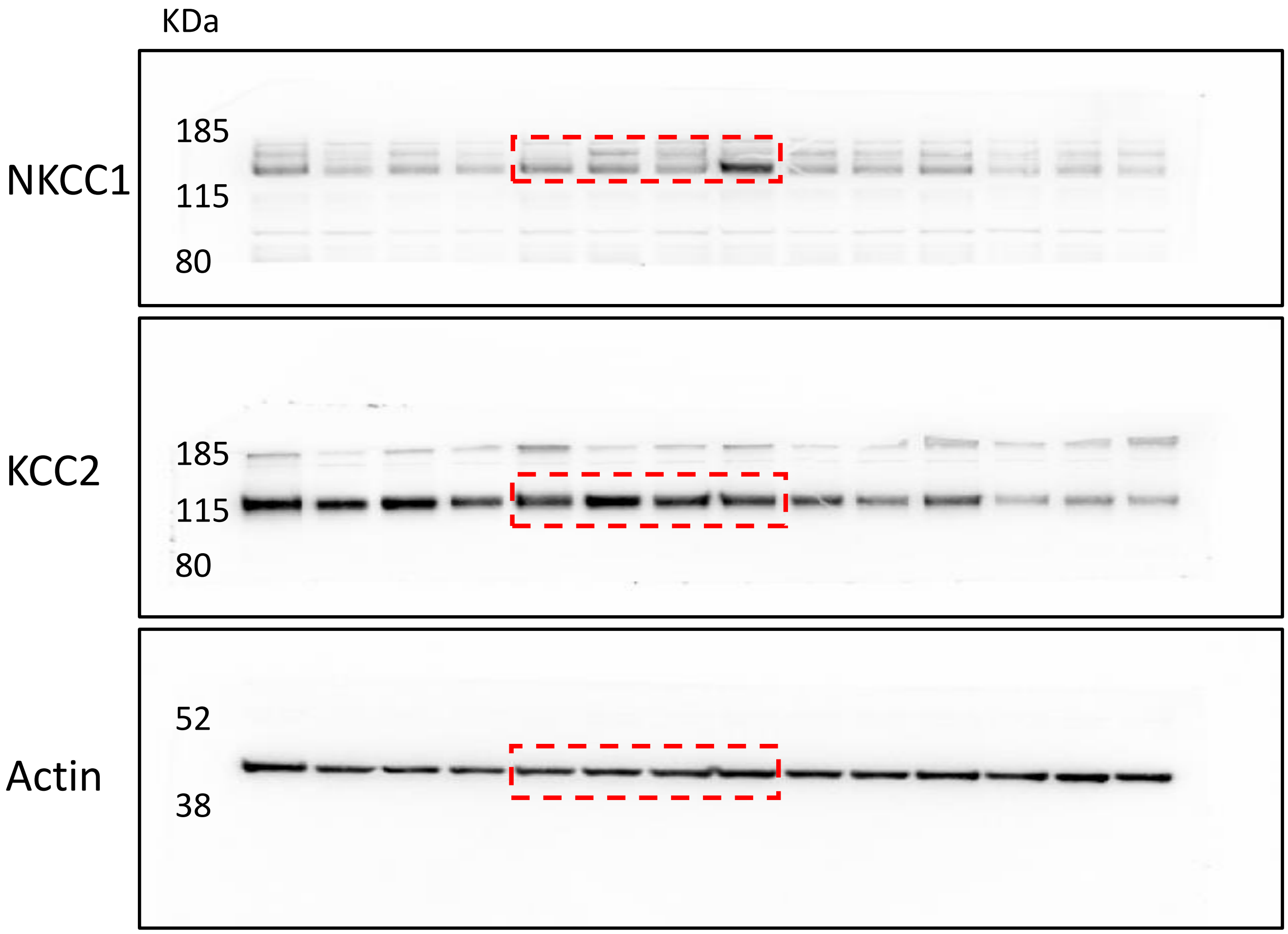

Figure S13. Uncropped blots images. Full-length blot images corresponding to the cropped western blot bands (red boxes) presented in Fig. S3F.

Protein name	CTRL P5	JB1 P5	Detection method (unit)	Matrix	Statistic
**IGF-1**	0.085 ± 0.02	0.082 ± 0.02	ELISA (pg/g)	Hippo campus	T test<br>P= 0.79
**IGF-1**	0.64 ± 0.42	0.91 ± 0.37	ELISA (pg/mL)	Plasma	Mann Whitney<br>P= 0.24
**IGFALS**	11.65 ± 0.78	12.59 ± 0.87	LCMS	Plasma	T-test<br>-log P value = 1.61<br>Fold change = 0.94
**IGFBP2**	7.78 ± 1.55	7.97 ± 2.22	LCMS	Plasma	T-test<br>-log P value = 0.08<br>Fold change = 0.19
**IGFBP3**	9.2 ± 2.34	10.10 ± 1.86	LCMS	Plasma	T-test<br>-log P value = 0.44<br>Fold change = 0.9
**IGFBP4**	7.72 ± 1.11	7.23 ± 1.56	LCMS	Plasma	T-test<br>-log P value = 0.35<br>Fold change = -0.49
**IGF-2**	7.3 ± 3.56	7.33 ± 2.17	LCMS	Plasma	T-test<br>-log P value = 0.01<br>Fold change = 0.03

Table S1. Systemic IGF-1R inhibition in WT mouse pups does not induce any significant acute change in IGF-1 hippocampal and plasma levels and in IGF-1-related proteins plasma levels. LCMS: Liquid Chromatography-Mass Spectrometry

	♂ & ♀				
	CTRL		JB1		T-test
	Mean	SEM	Mean	SEM	*P* value
Complex	1.2	0.44	2.5	0.51	0.06
Composite	0.1	0.07	0.0	0	0.17
2 component	18.5	1.37	16.9	1.96	0.50
Upward	0.3	0.18	0.6	0.21	0.31
Downward	12.3	1.55	11.4	1.15	0.67
Chevron*	1	0.53	3.1	0.74	0.024*
I-chevron	0.3	0.17	0.6	0.37	0.49
Short	5.3	1.14	2.8	0.71	0.07
Single harmonic	0.0	0	0.1	0.11	0.31
Frequency step	0.1	0.051	0.1	0.05	0.97
Flat	11.0	1.19	10.7	1.77	0.88

Table S2. USV call classification for data presented in the experiments reported in Fig. 2B,C. Early IGF-1R inhibition leads to increased chevron-type calls in P6 pups. Two-tailed Student's t-test, t = 2.345, *p<0.05.

♂ & ♀	CTRL		JB1		T-test
	Mean	SEM	Mean	SEM	P value
RMP (mV)	-68.25	0.81	-67.82	1.06	0.75
Input Resistance (MΩ)	207.40	14.28	234.20	13.62	0,18
AP threshold (mV)	-40.35	0.60	-40.59	0.77	0.59
AP amplitude (mV)	90.76	1.55	88.82	1.26	0.34
Half width (ms)	1,53	0,04	1,54	0,04	0,92
fAHP (mV)	11.94	0.56	11.29	0.67	0.46

♂	CTRL		JB1		T-test
	Mean	SEM	Mean	SEM	P value
RMP (mV)	-67.86	0.98	-67.83	1,36	0.99
Input Resistance (MΩ)	194.00	16.09	224.70	16.90	0.24
AP threshold (mV)	-39.86	0.97	-39.75	0.91	0.94
AP amplitude (mV)	90.14	2.39	89.08	0.97	0.64
Half width (ms)	1.49	0.03	1.58	0.04	0.14
fAHP (mV)	12.61	0.97	11.67	0,87	0.45

♀	CTRL		JB1		T-test
	Mean	SEM	Mean	SEM	*P* value
RMP (mV)	-68.78	1.31	-68.00	1.87	0.73
Input Resistance (MΩ)	224.20	20.83	257.20	21.3	0.34
AP threshold (mV)	-40.2	0.84	-42.60	0.60	0.08
AP amplitude (mV)	90.20	2.25	85.20	3.38	0.23
Half width (ms)	1.58	0.07	1.66	0.081	0.46
fAHP (mV)	11.40	0.65	10.40	0.87	0.38

Table S3. Passive and active electrophysiological properties of neurons recorded in Fig. 4. Early IGF-1R inhibition does not alter passive and active electrophysiological properties of CA1 hippocampal pyramidal neurons.

**Protein gene-name**	**Phosphorylation site**	**Statistic test (Student's t-test)**
Acin1	373	10.64165
Rps6kc1	577	5.181278
Rps6kc1	546	4.385646
Sptbn1	2127	5.424491
Srsf1	201	6.941797

Table S4. Hyperphosphorylated-phosphorylation sites differentially expressed between JB1-treated (N = 5 animals) and vehicle-treated (control, N = 4 animals) female pup littermates sacrificed 1 hour after the last treatment at P5. S0=0.1 and FDR=0.05.

Protein gene-name (A-I)	Phosphorylation site	Statistic test (Student's t-test)	Protein gene-name (I-Z)	Phosphorylation site	Statistic test (Student's t-test)
Agmo	415	-2.75485	*Iqsec1*	939	-2.76907
Ank2	3123	-2.99502	*Lrrfip2*	18	-2.76144
Ank2	3878	-2.72436	*Lrrfip2*	18	-3.04566
Arfgef1	234	-2.97643	*Lrrfip2*	18	-3.18555
Arfgef1	243	-2.97643	*Mapk1*	185	-2.38958
Arhgap32	1823	-2.07005	*Mapt*	112	-4.07528
Arhgef12	637	-3.35561	*Mapt*	112	-4.07528
Atad1	322	-2.09078	*Mical3*	685	-3.11605
Bptf	1249	-1.98586	*Mical3*	685	-3.11605
Bptf	1312	-1.98586	*Mical3*	685	-3.11605
Ccar2	626	-3.41	*Mical3*	685	-3.11605
Ccdc28b	46	-2.38911	*Myo9a*	1302	-3.24955
Ccdc28b	46	-2.38911	*Myo9a*	1302	-3.4745
Ccdc28b	46	-2.38911	*Myo9a*	1302	-3.5232
Ccdc28b	46	-2.38911	*Ncam1*	783	-2.33846
Cdc42ep1	207	-5.28126	*Ncam1*	788	-2.33846
Clasp1	1220	-2.05695	*Ncoa3*	1309	-3.07449
Clasp1	1221	-2.05695	*Ncoa3*	1304	-3.07449
D430041D05Rik	785	-2.09288	*Ncoa3*	1308	-3.07449
D430041D05Rik	1584	-2.09288	*Ncoa5*	34	-3.12711
D430041D05Rik	900	-2.09288	*Ncoa5*	29	-3.12711
Ddx23	14	-2.81302	*Palm*	124	-2.66797
Dlgap1	362	-2.19385	*Palm*	122	-2.66797
Dpysl3	522	-2.71149	*Pcdh17*	1130	-4.04368
Dpysl3	514	-2.40379	*Pitpnm2*	365	-2.84366
Eif3b	120	-2.51055	*Pitpnm2*	365	-2.84366
Eif4g3	483	-1.57006	*Pitpnm2*	365	-2.84366
Eif4g3	472	-1.57006	*Ppig*	354	-2.52254
Eif4g3	472	-1.57006	*Ppig*	356	-2.52254
Eif4g3	655	-1.57006	*Prcc*	157	-3.69236
Eif5b	137	-1.96441	*Prpf38b*	321	-2.17541
Epb41l1	30	-2.31562	*Ptprn2*	185	-6.153
Epb41l1	33	-2.31562	*Ptprn2*	185	-5.11052
Epb41l1	648	-2.04463	*Raf1*	296	-2.24023
Farp1	427	-2.66626	*Raf1*	316	-2.24023
Farp1	433	-2.66626	*Retreg3*	320	-1.83372
Gbf1	1298	-1.83356	*Rpl6*	21	-2.41381
Gphn	188	-2.25451	*Rps28*	23	-2.98253
Gphn	188	-2.25451	*Shank1*	605	-3.91537
Gphn	194	-2.25243	*Snx29*	815	-2.05786
Gphn	194	-2.25243	*Ssh2*	1217	-3.33958
Gphn	352	-3.02605	*Syn3*	461	-3.06431
Gprin2	22	-3.65089	*Taok2*	9	-2.33337
Grip1	719	-2.2623	*Tmem169*	62	-1.82172
Grip1	720	-2.2623	*Vps13d*	1395	-2.3236
Grip1	719	-2.2623	*Zdhhc14*	447	-2.39328
Hcn3	558	-3.44067	*Zdhhc14*	441	-2.39328
Hcn3	561	-3.44067	*Zdhhc14*	342	-2.39328
Hcn4	118	-2.75413	*Zdhhc14*	336	-2.39328
Hdac4	410	-5.38315	*Znf608*	635	-2.68335
Hdac4	581	-5.4548	*Zranb2*	83	-2.48165
Hnrnpu	247	-2.91761	*Zranb2*	120	-2.48165
Hsph1	558	-3.36991	*Zranb2*	83	-2.48165
Hsph1	517	-3.31769	*Zranb2*	120	-2.48165
Impact	137	-2.80496	*Zranb2*	14	-2.48165

Table S5. Dephosphorylated-phosphorylation sites differentially expressed between JB1-treated (N = 5 animals) and vehicle-treated (control; N = 4 animals) female pup littermates sacrificed 1 hour after the last treatment at P5. Note that, for this analysis, we used S0=0.1 and P value<0.01.

Protein gene name (A-M)	Phosphorylation site	Statistic test (Student's t-test)	Protein gene name (M-Z)	Phosphorylation site	Statistic test (Student's t-test)
Aatk	1283	3.844966	*Mllt10*	613	4.161875
Acin1	413	4.622269	*Mtus2*	1035	3.207467
Acin1	377	5.405424	*Parp8*	278	2.811684
Acin1	373	10.64165	*Parp8*	317	2.567705
Acin1	417	6.223553	*Pbrm1*	504	3.033031
Add1	64	3.209854	*Pbrm1*	504	3.224068
Ankle2	535	4.257792	*Phka2*	1015	4.565132
Arhgap39	384	2.297769	*Phka2*	1015	3.143363
Arhgap39	386	2.154481	*Ppp1r13l*	394	3.703333
Arhgap39	384	2.367985	*Ppp1r1a*	6	2.572673
Arid1a	1609	3.318087	*Rapgef2*	501	2.742462
Arid1a	1605	3.165254	*Rapgef2*	499	3.141305
Bcl11b	381	3.664484	*Rps6kc1*	577	6.462464
Bcl11b	664	3.745353	*Rps6kc1*	495	7.941914
Bcl9	257	4.815209	*Rps6kc1*	546	11.39056
Clasp2	637	4.18129	*Rps6kc1*	577	8.888326
Clasp2	637	4.057798	*Saal1*	6	5.782729
Clcn6	25	3.22788	*Sgsm1*	678	3.016883
Clip2	18	6.576934	*Sptbn1*	2127	9.399923
Ctnnd2	687	3.040741	*Srgap2*	930	2.293836
Ctnnd2	687	3.202556	*Srrm2*	755	3.128545
Dlg5	1011	3.164489	*Srrm2*	227	3.31666
Dlg5	988	2.9936	*Srrm2*	851	5.113951
Epb41l1	652	3.779792	*Srsf1*	201	8.849436
Foxk2	419	3.544302	*Srsf1*	201	7.645734
Hdgfl2	644	3.567024	*Synrg*	706	2.548884
Kdm4c	475	2.966056	*Tbc1d10b*	673	2.69345
Kif1a	1309	3.812307	*Tbc1d16*	122	4.059304
Larp1	626	3.684962	*Tcf3*	374	4.655327
Larp1	626	3.684962	*Tiam1*	231	2.286886
Lsr	192	3.318118	*Tnik*	552	4.37771
Map7d1	315	6.11601	*Tnik*	552	4.37771
Map7d1	315	5.794463	*Tnik*	581	4.37771
Mfap1a	116	3.136872	*Tnik*	581	4.37771
Mier1	185	2.878476	*Ubxn6*	113	3.242615
Mllt10	691	3.770905	*Vac14*	11	3.545355

Table S6. Hyperphosphorylated-phosphorylation sites differentially expressed between JB1-treated (N = 5 animals) and vehicle-treated (control; N = 4 animals) female pup littermates sacrificed 1 hour after the last treatment at P5. Note that, for this analysis, we used S0 = 0.1 and P value<0.01.

Kinase Enrichment Analysis			
Dephosphorylated		Hyperphosphorylated	
**Kinase**	**Mean Rank**	**Kinase**	**Mean Rank**
FYN	24.55	SRPK2	4.5
CSNK2A1	28.91	**GSK3B**	20.55
**GSK3B**	42.18	SRPK3	29.2
**SRC**	45.55	ABL1	37.45
ABL1	45.82	SRPK1	39.7
CDK5	46.45	FYN	40.91
RPS6AK3	53.27	CSNK2A1	42.64
**MAPK3**	57.27	**AKT1**	44
KSR2	57.86	**PRKCZ**	55.09
SGK1	59.5	EGFR	55.36
MNK1	61.22	**PRKCA**	57
RAF1	61.27	**PRKCB**	58
MAP2K1	61.55	SRC	59.45
**MAPK14**	64.09	**PRKCI**	59.73
**MAPK1**	64.18	CLK1	66
NTRK1	64.56	CLK3	66
CAMK2A	65.1	PRKCG	66.89
RPS6K1	66.5	CDK1	68.1
MAP2K2	67.33	**MAPK1**	69.82
SRPK2	70.6	CASK	70.25

Table S7. Kinase Enrichment Analysis of significantly dephosphorylated (left) and hyperphosphorylated (right) phospho-proteins corresponding to differentially expressed phosphorylation sites showed in Table S5,6. The kinases related to IGF-1 signalling are highlighted in bold.

SFARI Tool Analysis			
Dephosphorylated		Hyperphosphorylated	
Neuropsychiatric disease	P value (-log10)	Neuropsychiatric disease	P value (-log10)
ASD	2.0239	ASD	1.9083
ID	2.1302	ID	0.4289
NDD/DD	1.1552	NDD/DD	0.4366
EPS	2.2313	ADHD	1.0163
SCZ	0.9178	EPS	0.7639

Table S8. Enrichment for neuropsychiatric disorder risk-genes (identified with SFARI gene archive) in the dephosphorylated and hyperphosphorylated dataset shown in Table S5,6.

OMICS Analysis					
**Dephosphorylated**			**Hyperphosphorylated**		
**Analysis**	**GO TERM/ Pathway**	**-log10 (FDR)**	**Analysis**	**GO TERM/ Pathway**	**-log10 (FDR)**
Cellular Compatment	A band	1.777283529	Cellular Compatment	beta-catenin-TCF complex	1.306273051
Cellular Compatment	axonal growth cone	1.614393726	Cellular Compatment	cytoplasm	1.701146924
Cellular Compatment	cell body	1.440093375	Cellular Compatment	cytoskeleton	1.787812396
Cellular Compatment	cytoskeleton	3.995678626	Cellular Compatment	postsynaptic density	3.752026734
Cellular Compatment	dendrite	2.935542011	Cellular Compatment	SWI/SNF complex	2.096910013
Cellular Compatment	glutamatergic synapse	2.772113295	Molecular function	GTPase activator activity	2.266802735
Cellular Compatment	HCN channel complex	1.974694135	Molecular function	protein binding	2.435333936
Cellular Compatment	postsynaptic density	6.440093375	Reactome pathway	CDC42 GTPase cycle	1.522878745
Cellular Compatment	postsynaptic membrane	2.987162775	Reactome pathway	RHOU GTPase cycle	1.41453927
Cellular Compatment	postsynaptic specialization, intracellular component	1.510041521			
Cellular Compatment	pseudopodium	1.381951903			
Cellular Compatment	Schaffer collateral - CA1 synapse	1.342944147			
Biological Processes	cellular component organization	3.480172006			
Biological Processes	regulation of actin filament-based process	1.455931956			
Biological Processes	regulation of cellular component biogenesis	1.978810701			
Biological Processes	regulation of cytoskeleton organization	2.098541679			
Biological Processes	regulation of SA node cell action potential	2.126098402			
Molecular function	cytoskeletal protein binding	2.4867824			

Table S9. Gene Ontology (GO) analysis for the differentially dephosphorylated or hyperphosphorylated significantly expressed proteins corresponding to differentially expressed phosphorylation sites showed in Table S5,6.

♂					
**Analysis**	**CTRL Vehicle**	**JB1 Vehicle**	**CTRL GNX**	**JB1 GNX**	**F and P**
Myelinated axon density	62.98 ± 3.95	39.98 ± 6.76	56.33 ± 9.58	43.47 ± 1.27	TWO-WAY ANOVA $F_{Interaction}$ (1, 8) = 0.6653 P = 0.4383
G Ratio	0.67 ±0.0096	0.71 ± 0.0048	0.69 ± 0.003	0.69 ± 0.011	TWO-WAY ANOVA $F_{interaction}$ (1, 8) = 3.346 P = 0.1048

Table S10. GNX treatment (see Fig. 5) does not improve myelination deficit in JB1-treated adolescent mice.

Behavioral test cohorts
(Fig. 2, Fig. S1, S7,S8)

♂ & ♀				
**COHORT #**	**TEST 1**	**TEST 2**	**TEST 3**	**TEST 4**
1	NOR	Three-chamber	T maze	
2	T maze	NOR	Self-grooming	Three-chamber
3	T maze	OL	Self-grooming	Three-chamber
4	OL	EPM	Self-grooming	
5	OL	EPM		
6	OL	EPM		
7	EPM (females only)	T maze	Three-chamber	

Behavioral test cohorts for rescue experiments
(Fig. 5)

♂			
**COHORT #**	**TEST 1**	**TEST 2**	**TEST 3**
1M	NOR	Three-chamber	Self-grooming
2M	NOR	Three-chamber	Self-grooming
3M	Three-chamber	Self-grooming	NOR
4M	Three-chamber		

♀	
**COHORT #**	**TEST 1**
1F	EPM
2F	EPM
3F	EPM
4F	EPM

Table S11. Mouse cohorts subjected to behavioural testing reported in Fig. 2,5 and Fig. S1, S7, S8.

♂ & ♀			
**Objects**	**CTRL**	**JB1**	**F and P**
A	32.98 ± 2.18	33.11± 1.04	TWO-WAY ANOVA $F_{interaction}$ (2, 84) = 0.1497 P = 0.8612
B	33.68 ± 1.83	33.80 ±1.04	
C	33.34 ± 1.18	33.10±1.21	

♂			
**Objects**	**CTRL**	**JB1**	**F and P**
A	32.80 ± 1.10	33.10 ± 1.04	TWO-WAY ANOVA $F_{interaction}$ (2, 51) = 2.230 P = 0.1179
B	33.60 ± 1.28	33.10 ± 1.09	
C	33.61 ± 0.80	32.90 ±1.27	

♀			
**Objects**	**CTRL**	**JB1**	**F and P**
A	33.36 ± 3.70	33.13 ± 1.17	TWO-WAY ANOVA $F_{interaction}$ (2, 24) = 0.1937 P = 0.8252
B	33.84 ± 2.82	33.39 ± 1.10	
C	32.79 ± 1.69	33.48 ± 1.11	

Table S12. % object preference in the NOR test for CTRL and JB1-treated mice in the experiments reported in Figure 2G and Fig. S7C.

♂ & ♀			
**Objects**	**CTRL**	**JB1**	**F and P**
Acquisition	37.77 ± 12.24	33.04 ± 12.85	Mann Whitney test P = 0.1592
Trial	35.47 ± 9.255	41.03 ± 7.192	T- test P = 0.0765

♂			
**Objects**	**CTRL**	**JB1**	**F and P**
Acquisition	41.75 ± 13.03	35.57 ± 14,96	T-test P = 0.3382
Trial	35.61 ± 8.622	41.69 ± 7.748	T-test P = 0.1141

♀			
**Objects**	**CTRL**	**JB1**	**F and P**
Acquisition	29.82 ± 4.836	27.99 ± 5.132	T-test P = 0.5761
Trial	35.18 ± 11.50	39.71 ± 6.533	T-test P = 0.4661

Table S13. Total Exploration time (s) in the NOR test for CTRL and JB1-treated mice in the experiments reported in Figure 2G and Fig. S7C.

♂ & ♀			
**Objects**	**CTRL**	**JB1**	**F and P**
A	50.25 ± 2.93	45.40 ± 4.79	TWO-WAY ANOVA<br>$F_{interaction}$ (1. 46) = 19.46<br>P < 0.0001<br>Sidak Post Hoc test<br>P<0.01
B	49.75 ± 2.93	54.60 ± 4.79	

♂			
**Objects**	**CTRL**	**JB1**	**F and P**
A	52.00 ± 2.48	45.15 ± 5.34	TWO-WAY ANOVA<br>$F_{interaction}$ (1, 26) = 16.10<br>P = 0.0005<br>Sidak Post Hoc test<br>P<0.05
B	48 ± 2.48	54.85 ± 5.34	

♀			
**Objects**	**CTRL**	**JB1**	**F and P**
A	49.00 ± 2.69	46.26 ± 3.27	TWO-WAY ANOVA<br>F (1, 16) = 3.899<br>P = 0.0658
B	51.00 ± 2.69	53.74 ± 3.27	

Table S14. % object preference in the OL test for CTRL and JB1-treated mice in the experiments reported in Figure 2H and Fig. S7D.

♂ & ♀			
**Objects**	**CTRL**	**JB1**	**F and P**
Acquisition	32.61 ± 4.637	32.16 ± 5.465	T-test<br>P = 0.8283
Trial	27.05 ± 7,162	27.93 ± 7.870	Mann Whitney test<br>P = 0.9624

♂			
**Objects**	**CTRL**	**JB1**	**F and P**
Acquisition	31.15 ± 5.757	31.31 ± 5.942	T-test<br>P = 0.9617
Trial	30.55 ± 9.559	30.10 ± 7.726	T-test<br>P = 0.9224

♀			
**Objects**	**CTRL**	**JB1**	**F and P**
Acquisition	33.49 ± 3,668	35.01 ± 2.136	T-test<br>P = 0.5296
Trial	24.54 ± 3,943	20.71 ± 1.086	T-test<br>P = 0.1465

Table S15. Total Exploration time (s) in the OL test for CTRL and JB1 treated mice in the experiments reported in Figure 2H and Fig. S7D.

% Object preference NOR

♂					
**Objects**	**CTRL Vehicle**	**JB1 Vehicle**	**CTRL GNX**	**JB1 GNX**	**F and P**
A	32.84 ± 3.07	33.20 ± 3.19	33.51 ± 7.37	36.47 ± 5.45	THREE-WAY ANOVA<br>$F_{interaction}$ (2, 132) = 1.574<br>P = 0.2111
B	33.15 ± 5.65	35.04 ± 2.61	33.61 ± 6.17	31.38 ± 5.08	
C	34.01 ± 3.08	31.76 ± 4.62	32.88 ± 5.70	32.15 ± 6.59	

Total Exploration Time (sec) NOR

♂					
**Objects**	**CTRL Vehicle**	**JB1 Vehicle**	**CTRL GNX**	**JB1 GNX**	**F and P**
Acquisition	42.56 ± 13.41	39.48 ± 16.21	55.57 ± 8.35	53.07 ± 12.83	TWO-WAY ANOVA<br>$F_{interaction}$ (1, 46) = 0.005612<br>P = 0.9406
Trial	34.22 ± 8.60	37.01 ± 10.26	26.96 ± 4.65	24.96 ± 4.93	TWO-WAY ANOVA<br>$F_{interaction}$ (1, 43) = 1.021<br>P = 0.3180

Table S16. % object preference and Total Exploration time for CTRL and JB1-treated mice upon vehicle or ganaxolone administration as adolescents in the NOR test in the experiments reported in Figure 5F.

	Females (n 31)	Males (n 20)	p§	Total (n 51)
Prenatal features				
Multiple gestation, *n (%)*	12 (38.7%)	6 (30.0%)	*.565*	18 (35.3%)
Monochorial twins, *n* (%)	2 (6.5%)	3 (15.0%)	*.369*	5 (9.8%)
IUGR, *n (%)*	8 (25.8%)	9 (45.0%)	*.225*	17 (33.3%)
Maternal hypertension	8 (25.8%)	4 (20.0%)	*.743*	12 (23.5%)
Gestational diabetes	3 (9.7%)	0 (0.0%)	*.271*	3 (5.9%)
Absent/incomplete antenatal steroid course, *n (%)*	25 (80.6%)	15 (78.9%)	*.732*	40 (80.0%)
pPROM, *n (%)*	8 (25.8%)	5 (25.0%)	*.999*	13 (25.5%)
Intrapartum features				
Birth by caesarean section, *n (%)*	27 (87.1%)	17 (85.0%)	*.999*	44 (86.3%)
Gestational age (weeks), *mean (sds)*	28.1 (2.1)	27.9 (2.0)	*.732*	28.0 (2.1)
Birth weight (grams), *mean (sds)*	1004.2 (280.0)	1028.5 (270.6)	*.714*	1013.7 (273.9)
Apgar at 1st minute, *mean (sds)*	5.4 (1.8)	5.6 (2.0)	*.566*	5.5 (1.8)
Apgar at 5th minute, *mean (sds)*	7.4 (1.1)	8.0 (1.4)	***.023***	7.6 (1.2)
Postnatal features				
Intubation during first 72 h, *n (%)*	7 (22.6%)	8 (40.0%)	*.219*	15 (29.4%)
HFO during first 72 h, *n (%)*	5 (16.1%)	3 (15.0%)	*.999*	8 (15.7%)
Pneumothorax during first 72 h, *n (%)*	0 (0%)	0 (0%)	-	0 (0%)
Inotrope use during first 72 h, *n (%)*	2 (6.5%)	3 (15.0%)	*.369*	5 (9.8%)
MV > 28 days, *n (%)*	4 (12.9%)	2 (10.0%)	*.999*	6 (11.8%)
Multiple surfactant doses, *n (%)*	10 (32.3%)	7 (35.0%)	*.999*	17 (33.3%)
EOS, *n (%)*	4 (12.9%)	1 (5.0%)	*.636*	5 (9.8%)
LOS, *n (%)*	15 (48.4%)	12 (60.0%)	*.567*	27 (52.9%)
NEC, *n (%)*	6 (19.4%)	2 (10.0%)	*.456*	8 (15.7%)
Surgery for NEC, *n (%)*	3 (9.7%)	1 (5.0%)	*.999*	4 (7.8%)
PDA, *n (%)*	16 (51.6%)	15 (75.0%)	*.083*	31 (60.8%)
Surgery for PDA, *n (%)*	5 (16.1%)	3 (15.0%)	*.619*	8 (15.7%)
BPD, *n (%)*	2 (6.4%)	1 (5%)	.999	3 (5.9%)
ROP, *n (%)*	15 (48.4%)	9 (45.0%)	*.999*	24 (47.1%)

Table S17. Prenatal, intrapartum and postnatal features of the investigated cohort of ex- premature children presented in Fig. S11 and Fig. S12. Legend. BPD, bronchopulmonary dysplasia; EOS, early-onset sepsis; GA, gestational age; IUGR, intrauterine growth restriction; HFO, high-frequency oscillations; MV, mechanical ventilation; NEC, necrotizing enterocolitis; EOS, early-onset sepsis; LOS, late-onset sepsis; PDA, patent ductus arteriosus; pPROM, premature prolonged rupture of membranes; ROP, retinopathy of prematurity**.**

	Females (n 31)	Males (n 20)	p§	Total (n 51)
Total Developmental Quotient at 1 year of corrected age *mean (sds) / median (min-max)*	99.25 (11.3) / 99.5 (81-128)	98.6 (11.6) / 100 (72-127)	*.814*	98.9 (11.4) / 100 (72-128)
Total Intelligence Quotient at 5 years *mean (sds) / median (min-max)*	95.7 (11.91) / 95 (63-121)	93.1 (12.11) / 93.5 (62-113)	*.589*	94.2 (12.1) / 95 (62-121)

Table S18. Neurodevelopmental and cognitive quotients of the investigated cohort of ex-premature children presented in Fig. S11 and Fig. S12.
